# Synthesis of Novel 2-(Het)arylpyrrolidine Derivatives and Evaluation of Their Anticancer and Anti-Biofilm Activity

**DOI:** 10.3390/molecules24173086

**Published:** 2019-08-25

**Authors:** Andrey Smolobochkin, Almir Gazizov, Marina Sazykina, Nurgali Akylbekov, Elena Chugunova, Ivan Sazykin, Anastasiya Gildebrant, Julia Voronina, Alexander Burilov, Shorena Karchava, Maria Klimova, Alexandra Voloshina, Anastasia Sapunova, Elena Klimanova, Tatyana Sashenkova, Ugulzhan Allayarova, Anastasiya Balakina, Denis Mishchenko

**Affiliations:** 1 Arbuzov Institute of Organic and Physical Chemistry, FRC Kazan Scientific Center of RAS, Arbuzov str., 8, Kazan 420088, Russia; 2 Southern Federal University, Stachki Avenue, 194/2, Rostov-on-Don 344090, Russia; 3 Institute of Chemical Research and Technology of Korkyt Ata Kyzylorda State University, Aiteke bie str., 29A, Kyzylorda 120014, Kazakhstan; 4 Kazan Federal University, Kremlyovskaya str., 18, Kazan 420008, Russia; 5 N. S. Kurnakov Institute of General and Inorganic Chemistry, RAS, 31 Leninsky Av., Moscow 119991, Russia; 6 Institute of Problems of Chemical Physics RAS, Chernogolovka 142432, Russia; 7 Scientific and Educational Center in Chernogolovka of Moscow Region State University, Mytishi 141014, Russia

**Keywords:** pyrrolidine, carboxamide, anti-tumor activity, anti-cancer activity, cytotoxicity, apoptosis, bacterial biofilm, anti-bacterial activity

## Abstract

A library of novel 2-(het)arylpyrrolidine-1-carboxamides were obtained via a modular approach based on the intramolecular cyclization/Mannich-type reaction of *N*-(4,4-diethoxybutyl)ureas. Their anti-cancer activities both in vitro and in vivo were tested. The in vitro activity of some compounds towards M-Hela tumor cell lines was twice that of the reference drug tamoxifen, whereas cytotoxicity towards normal Chang liver cell did not exceed the tamoxifen toxicity. In vivo studies showed that the number of surviving animals on day 60 of observation was up to 83% and increased life span (ILS) was up to 447%. Additionally, some pyrrolidine-1-carboxamides possessing a benzofuroxan moiety obtained were found to effectively suppress bacterial biofilm growth. Thus, these compounds are promising candidates for further development both as anti-cancer and anti-bacterial agents.

## 1. Introduction

**Figure 1:**
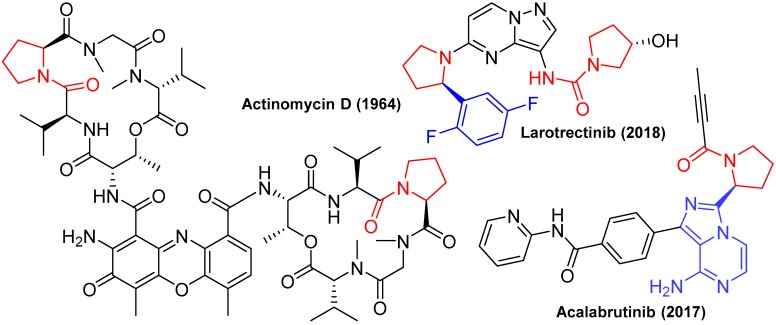
Approved anti-cancer drugs possessing 2-substituted *N*-carboxypyrrolidine scaffold.

The pyrrolidine moiety is an important structural part of many natural alkaloids [1,2,3,4] and one of the most frequently occurring heterocyclic scaffolds in approved drugs [5]. A number of anti-cancer drugs possess an *N*-carboxypyrrolidine scaffold. These include both fairly old ones (e.g., dactinomycin [6], approved in 1964) and those that have appeared recently. Acalabrutinib [7,8] (approved by FDA in 2017) and larotrectinib [9,10] (approved by the FDA in 2018) may serve as illustrative examples (Figure 1). Notably, larotrectinib is the first drug to be specifically developed and approved to treat any cancer containing certain mutations, as opposed to cancers of specific tissues. It should also be emphasized that both acalabrutinib and larotrectinib contain a 2-(het)aryl-substituted pyrrolidine fragment. Considering that in the past few decades, cancer has been one of major causes of death in most countries of the world [11], a search for novel anti-cancer drug candidates among 2-(het)aryl-*N*-carboxypyrrolidine derivatives is undoubtedly a promising field of research.

Despite the increasing number of research works aimed at obtaining and studying 2-(hetaryl)pyrrolidines, the synthesis of these compounds still meets certain difficulties. Approaches to these compounds can be divided into two main groups. The first one includes the modification of an existing pyrrolidine fragment. Various cross-coupling reactions of (hetero)aromatics with appropriately substituted pyrrolidine derivatives [12,13,14,15,16,17], including oxidative [18,19,20] and photooxidative ones [21,22,23,24], are the most often used within this pathway. In several cases, the synthesis of enantiomerically pure 2-(het)arylpyrrolidines has been also accomplished by decarboxylative (hetero)arylation of proline derivatives [25,26,27,28,29,30,31]. The second approach is based on the formation of a pyrrolidine ring from acyclic precursors. Within this approach, the intermolecular [3+2] dipolar cycloaddition of activated alkenes to azomethine ylides plays a significant role [32,33,34,35,36,37]. Essential drawbacks of the abovementioned approaches are the need of expensive metal catalysts and/or harsh reaction conditions, as well as the need of the preliminary synthesis of starting compounds with appropriate functional groups and desired fragments. Hence, methods employing inexpensive, readily available reagents and catalysts and allowing simultaneous pyrrolidine ring closure and C–C_hetaryl_ bond formation are of a special interest.

**Scheme 1:**
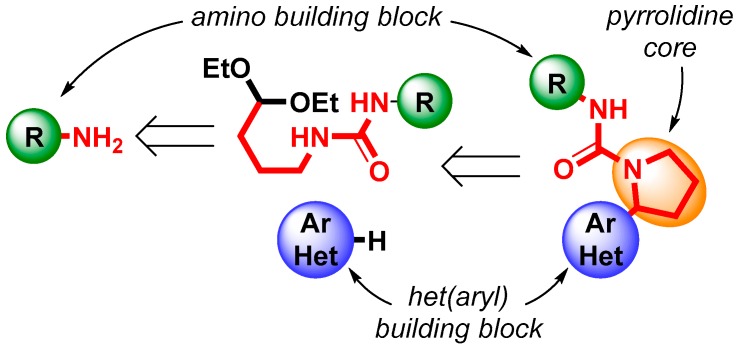
Modular synthetic approach to 2-(het)arylpyrrolidine-1-carboxamides.

Earlier, we developed a metal-free approach to 2-arylsubstitued pyrrolidine derivatives based on the acid-catalyzed intramolecular cyclization of *N*-(4,4-diethoxybutyl)ureas, leading to the formation of a pyrrolinium cation. Further Mannich-type reaction of this cyclic iminium ion with various electron-rich aromatic *C*-nucleophiles allowed us to obtain a range of 2-arylpyrrolidine-1-carboxamides [38,39,40,41,42,43,44,45]. The main benefits of this approach are the usage of easily accessible starting materials and its modularity, which allows wide variability in both the aromatic moiety and substituents at the nitrogen atom (Scheme 1).

Herein, we report the successful extension of this approach to the synthesis of novel 2-(het)aryl-substituted pyrrolidine-1-carboxamides, as well as the evaluation of their anti-cancer activities in vivo and in vitro and studies of the inhibition of bacterial biofilm growth.

## 2. Results and Discussion

### 2.1. Chemistry

We started our research from the synthesis of initial *N*-(4,4-diethoxybutyl)ureas **1** by the previously described procedure [38,46]. Both aliphatic amines and substituted anilines were employed as the amine building block (see Scheme 1). It is well known that the ionization of an amine group is widely used for drug solubility enhancement [47,48]. Thus, *N*-(4,4-diethoxybutyl)urea **1h** possessing a dimethylamino moiety was also obtained (Table 1).

**Scheme 2:**
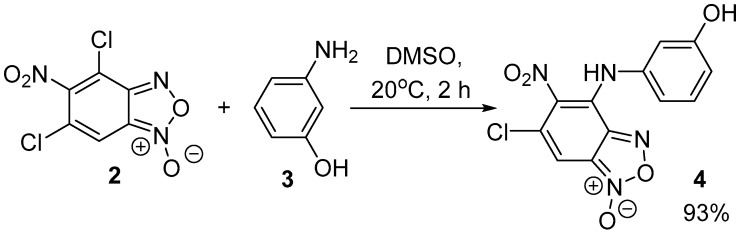
Synthesis of substituted phenol **4** possessing a benzofuroxan fragment.

The choice of aromatic (sesamol [49,50]) and heterocyclic (4-hydroxycoumarin [51], 4-hydroxy-6-methyl-2H-pyran-2-one [52,53]) *C*-nucleophiles was determined by both their well-known biological activity and high reactivity in electrophilic substitution reactions. One more heterocyclic scaffold, namely, the benzofuroxan moiety, was also of interest due to its biological properties [54,55,56] and ability to serve as NO donor [57,58]. However, it could not be introduced to the target pyrrolidines directly due to its extremely low nucleophilicity. Thus, the phenol derivative **4** was used instead, which was obtained by the reaction of 4,6-dichloro-5-nitrobenzofuroxan **2** with 3-aminophenol **3** (Scheme 2).

**Scheme 3:**
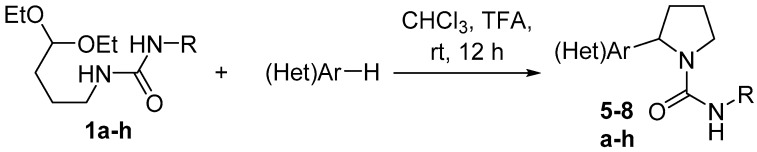
Synthesis of (het)arylpyrrolidine-1-carboxamides **5**–**8**.

Next, we carried out the reaction of ureas **1a**–**h** with these *C*-nucleophiles in the presence of trifluoroacetic acid as catalyst (Scheme 3). As a result, a library of 28 novel 2-(het)arylpyrrolidine-1-carboxamides **5b**–**h**, **6b**–**h**, **7b**–**h**, **8b**–**h** was obtained, which included all possible combinations of amine and (het)aryl building blocks. Additionally, the non-substituted pyrrolidine-1-carboxamide **5a** was also obtained. Yields of target compounds varied from moderate to excellent (Table 1). The substitution sites of the used *C*-nucleophiles were confirmed by NMR data. The structure of compound **8f** was additionally confirmed by X-ray analysis data.

**Figure 2:**
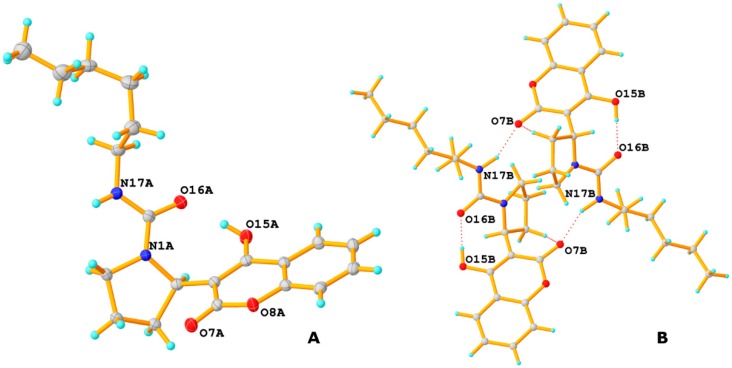
(**A**) Molecular structure of compound **8f** in crystal (on the example of molecule a). Ellipsoids are shown with 50% probability; (**B**) H-bonded dimer in crystal of **8f** (on the example of molecule b).

Compound **8f** crystallized with three independent molecules in the unit cell. Bond lengths, valence, and torsion angles were within the intervals typical for each bond type (Figure 2A). Molecules a, b, and c differed in the conformations of the pyrrolidine cycle (envelope in each case, however in molecules a and c atom C3 was out of the plane formed by other atoms, while in molecule b it was atom C4 and hexane substituent (Supplementary Materials, Table S1). Crystal packing consisted of centrosymmetric H-bonded dimers in which each independent molecule interacted only with its symmetric equivalent (Figure 2A, Supplementary Materials, Table S2). Dimers formed columns via π···π interactions (Supplementary Materials, Table S3). The three-dimensional system (Supplementary Materials, Figure S15) was formed by weaker CH···O (Supplementary Materials, Table S1) and CH···π interactions (Supplementary Materials, Table S4).

### 2.2. Biological Studies

#### 2.2.1. In Vitro Studies of Anti-Cancer Activity

Cytotoxic assay. The resulting compounds were tested for cytotoxicity against normal and cancerous human cell lines at concentrations of 1–100 µM. The pyrrolidines **6b**–**h** containing the benzofuroxane fragment were found to be the most active, while the others (**5a**–**5h**, **7b**–**7h**, **8b**–**8h**) did not show anti-cancer activity (Table 2, Supplementary Materials, Table S5). Table 2 shows the IC_50_ data for compounds **6b**–**h**. It can be seen that in relation to the M-Hela cancer line, IC_50_ values for substances **6d**, **6c**, and **6e** were comparable to the reference compound tamoxifen. Compound **6g** (IC_50_ −14.7 μM) was most active against cervical cancer. At the same time, with regard to the Chang’s liver cell line, all test compounds were less toxic than tamoxifen.

**Figure 3:**
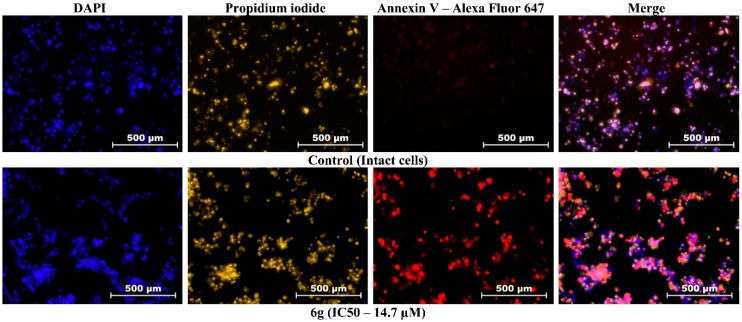
Images of control intact M-Hela cells (**top**) and M-Hela cells after treatment with **6g** at an IC_50_ concentration of 14.7 μM (**bottom**), obtained using the Cytell Cell Imaging multifunctional system using the BioApp Automated Imaging application. Annexin V-Alexa Fluor 647 (red fluorescence) was used to detect apoptotic cells; living cells—DAPI (blue fluorescence); dead cells—propidium iodide (yellow fluorescence).

Induction of apoptotic effects by test compounds. For compound **6g**, the ability to induce apoptosis in the human cancer cell line M-Hela was studied. As shown in Figure 3 (bottom), after 24 hours of treatment with compound **6g**, apoptotic effects were observed in M-Hela cells (red fluorescence). The data presented in Figure 3 show that at concentrations corresponding to the IC_50_ value, compound **6g** induced apoptosis in 25% of M-Hela cells. The top row of images in Figure 3 (control) presents images of intact M-Hela cells. It is seen that apoptotic effects were weakly expressed. The results show that the cytotoxicity of compound **6g** in M-Hela cancer cells was caused by an apoptotic pathway.

Multiplex analysis of early apoptosis markers. Next, using the MILLIPLEX^®^ MAP 7-plex Early Phase Apoptosis Signaling kit, seven markers of early apoptosis of JNK, Bad, Bcl-2, Akt, Caspase-9, p53, and Caspase-8 were detected in M-Hela cell lysates. The median fluorescence intensity (MFI) was measured using the Luminex^®^ system. This assay is a quick and convenient alternative to Western blot and immunoprecipitation.

**Figure 4:**
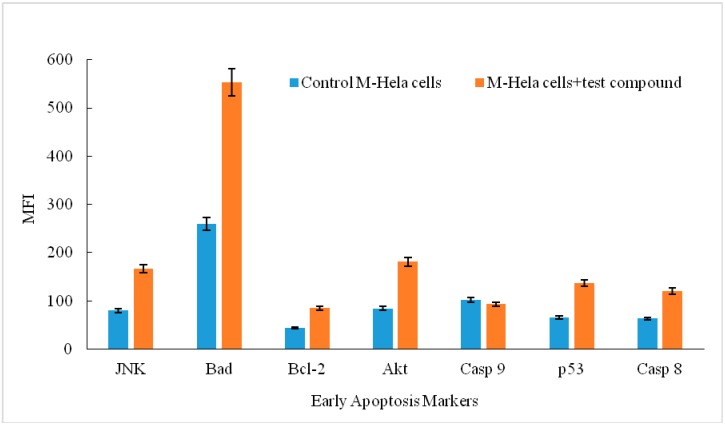
Multiplex analysis of early apoptosis markers in M-Hela cells treated with the test substance **6g** at an IC_50_ concentration of 14.7 µM as well as M-Hela cells untreated with the test substance (control). The median fluorescence intensity (MFI) was measured using the Luminex^®^ system. The graph shows the mean and standard deviation values for the wells in triplicate.

Figure 4 shows that the fluorescence intensity of Caspase-8 in the experimental sample (after exposure to the test substance **6g** was two times higher than that in the control. The results suggest that apoptosis proceeded along the extrinsic pathway of activation of Caspase-8 (death is initiated by activation of the surface cell receptor), and not along the intrinsic pathway associated with the activation of Caspase-9 (fluorescence intensity at the control level) in which death occurs due to mitochondrial dysfunction. This assumption was also confirmed by the predominance of pro-apoptotic Bad proteins over anti-apoptotic Bcl-2, which are responsible for irreversible cellular damage in mitochondrial processes. At the same time, apoptosis in M-Hela cells can be induced by activating the transcription of many pro-apoptotic genes by the transcription factors AP1 (signaling pathway activated by JNK stress) and p53 (response to DNA damage).

**Figure 5:**
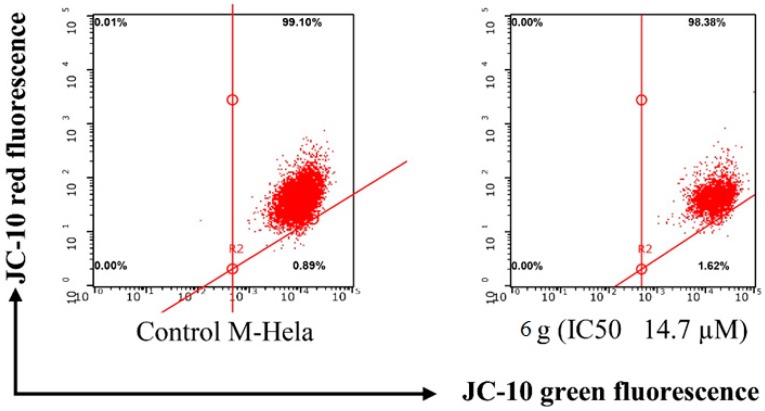
Flow cytometry analysis of M-Hela cells treated with compound **6g**, along with the quantification of % of cells with red aggregates. The values are presented as mean ± SD.

Effects on the mitochondrial membrane potential (Δψm) by lead compounds. In confirmation of data on the course of apoptosis in cells along an external pathway not associated with dysfunctional mitochondria, the ability of the tested compounds to reduce the potential of the mitochondrial membrane (Δψm) in M-Hela culture cells was examined using the example of compound **6g**. Studies were performed using flow cytometry methods using JC-10 reagent. In normal cells with a high potential of the mitochondrial membrane, the dye JC-10 forms aggregates (J-aggregate) near the mitochondrial membranes. When the membrane potential due to the stimulation of apoptosis falls, JC-10 is evenly distributed in the cell as a monomer (J-monomer). JC-10 units in normal cells have red fluorescence, and JC-10 monomers are green. The ratio between orange-red and green fluorescence can be used to assess the onset of apoptosis. No decrease in Δψm was demonstrated using flow cytometry analysis (Figure 5). The intensity of red fluorescence after treating cells with compound **6g** in an IC_50_ concentration of 14.7 μM did not actually change compared with the control. The results obtained indicate that the mechanism of action of the studied compounds is not associated with the induction of apoptosis, which proceeds along the mitochondrial pathway.

#### 2.2.2. In Vivo Studies of Anti-Cancer Activity

In vivo evaluation was performed on the syngeneic P388 murine leukemia. Murine leukemia models have been an essential component of the initial drug discovery programs since the 1970s. P388 leukemia played a major role in the screening of potential antitumor agents. Today, the majority of currently used clinical drugs were first detected by the murine leukemias. These models are suitable for the initial evaluation of the antitumor activity of new compounds [59]. The compounds were administered i.p. (thus, as an intra-tumor treatment), which was believed to maximize exposure and limit pharmacokinetic influences. Due to the fact that as a solvent for parenteral administration of the substance it is permissible to use any physiologically appropriate solvent that does not cause local irritating effects such as water or saline [60], we selected only water-soluble compounds **5b**, **5h**, **6h**, and **7h** for in vivo studies.

We found that the compounds **5b**, **5h**, and **8h** had pronounced anti-leukemic activity with the number of surviving animals on day 60 of observation from 17% to 83% and increased life span (ILS) from 80% to 447% (Table 3, Table 4, and Supplementary Materials, Figure S16). Compound **5h** had the greatest anti-leukemic activity. Indeed, in the group of mice that received intraperitoneal administration of compound **5h** at a dose of 40 mg/kg/day, 1, 5, and 9 days after tumor transplantation, 83% of the animals remained alive, and ILS was 447%. In general, the studied compounds can be arranged in the following order of reducing the antitumor activity during therapy of P388 leukemia: **5h** > **8h** > **5b**. However, compounds **6h** and **7h** in the dose range and the mode of administration used did not show antitumor properties. Thus, **5b**, **5h**, and **8h** can be recommended as promising compounds for the creation of new anticancer drugs.

#### 2.2.3. Anti-Biofilm Activity of Pyrrolidine-1-Carboxamides Possessing Benzofuroxan Moiety

Biofilms are communities of microorganisms attached to the surface or the interface in which cells are immersed in an exopolymer matrix consisting of polysaccharides, proteins, and DNA [61]. More than 90% of microorganisms occurring in nature exist in the form of biofilms [62]. Microbial biofilms are responsible for the etiology and pathogenesis of many acute and especially chronic bacterial infections in humans [63]. Among bacterial diseases in humans and animals, more than 80% are associated with the presence of stable bacterial communities enclosed in biofilms [64,65].

The possibility of interspecies community formation in biofilms along with high antibiotic resistance of many pathogenic and potentially pathogenic microorganisms make them almost invulnerable. The drug resistance of bacteria living in biofilms has increased manyfold in comparison to planktonically grown bacteria [66,67]. In this regard, the ability of pathogenic bacteria to form biofilms is a significant problem.

Moreover, in around 20% of all cases, microbial organisms are the causative agents of cancer-inducing inflammation. It is unclear if these microorganisms are causally involved in tumorigenesis, or if they benefit from the consequences of tumor growth and in turn promote tumor progression [68,69]. Furthermore, the work of Samanta et al. [70] demonstrates that the mechanisms behind advanced anti-biofilm and anticancer activities are linked to the generation of excess labile toxic reactive oxygen species (ROS). Such toxic ROS species cause the rapid oxidation and deterioration of cellular membranes. The unity of the action mechanism possibly testifies to the interconnection of anti-biofilm and anticancer activities in certain substances. Taking this into account, we also evaluated the ability of pyrrolidine-1-carboxamides **6a**–**e** possessing a benzofuroxan moiety and the initial benzofuroxan **4** to inhibit bacterial biofilm growth.

A staining assay was performed to estimate the extent of biofilm formation by *Vibrio aquamarinus* DSM 26054 and *Acinetobacter calcoaceticus* VKPM B-10353 under treatment with **6a**, **6b**, **6c**, **6d**, **6e**, and **4** (doses of 1 × 10^−9^–1 × 10^−5^ M). The natural strains *A. calcoaceticus* VKPM B-10353 and *V. aquamarinus* DSM 26054 were chosen as models due to their ability to actively form biofilms and for their extreme degree of similarity to pathogenic species. The obtained biological activity results are summarized in Table 5 (see Supplementary Materials, Figures S1–S14 for additional data). The compounds were evaluated for biofilm production compared to control. The results showed that compounds exhibited a variable degree of anti-biofilm activity against *V. aquamarinus* DSM 26054 and *A. calcoaceticus* VKPM B-10353.

Substances **6b** and **4** had different effects on *A. calcoaceticus* VKPM B-10353 biofilm formation—both suppressing and stimulating, depending on the concentration. Due to the dual nature of their action, they are not recommended for use as agents suppressing biofilm development.

For substances **6a**, **6d**, **6c**, and **6e**, the suppressive effect of different values on the formation of biofilm strains *V. aquamarinus* DSM 26054 and *A. calcoaceticus* VKPM B-10353 was registered in the range of investigated concentrations. Biofilm formation in comparison with control varied from 6.46% to 87.92%.

Biofilm formation by *A. calcoaceticus* VKPM B-10353 was less suppressed in the presence of substances than biofilm formation by *V. aquamarinus* DSM 26054. Biofilm formation varied from 42.37% to 87.92% with respect to control in the presence of the studied substances. For the *V. aquamarinus* DSM 26054 strain, it ranged from 6.46% to 67.29%.

Substances actively inhibited the growth of *A. calcoaceticus* VKPM B-10353 biofilms at the concentrations of 1 × 10^−8^ M and 1 × 10^−7^ M. The maximum inhibition of biofilms was registered under the influence of **6a** and **6d** at the concentration of 1 × 10^−8^ M, and biofilm formation was 42.37% and 46.95%, respectively.

The maximum inhibition of *V. aquamarinus* DSM 26054 biofilm was caused by **6a** and **6d** at the concentration of 1 × 10^−8^ M (biofilm formation was 10.23% and 6.46% in comparison with the control) and **6c** and **6e** at the concentration of 1 × 10^−7^ M (biofilm formation amounted to 9.22% and 11.01%).

Note that **6a** and **6c** actively suppressed the formation of *V. aquamarinus* DSM 26054 biofilm at the minimum concentration of 1 × 10^−9^ M—the preservation of biofilm was 17.7% and 13.54%, respectively.

The tested compounds were also compared with standard antibiotics azithromycin (see Supplementary Materials for additional data). Azithromycin exhibited an insignificant suppression of biofilm at high concentrations. Azithromycin suppressed the intensity of biofilm formation by *V. aquamarinus* DSM 26054 at the concentration of 1 × 10^−5^ M (Supplementary Materials, Figure S13). The optical density was 81.5% of the control values. The inhibitory effect of azithromycin in the studied concentrations on biofilm formation by *A. calcoaceticus* VKPM B-10353 was not detected (Supplementary Materials, Figure S14).

Taken together, pyrrolidine-1-carboxamides **6a**, **6c**, **6d**, and **6e** possessed a greater potential to suppress the formation of biofilms compared to the initial substance, as well as to the antibiotic azithromycin, and are promising as agents suppressing biofilms.

Genotoxicity and pro-oxidant characteristics of pyrrolidine-1-carboxamides possessing benzofuroxan moiety. All compounds in the same concentrations used for biofilm attenuation were also tested with *Escherichia coli* MG1655 (pRecA-lux). This strain was used for the evaluation of genotoxicity (Table 6). Bioluminescent response to DNA damage was detected for compounds **6b**, **6c**, and **6e**. The detected genotoxic effect was evaluated as medium (I > 2) for **6b**, **6c**, and **6e** in the concentration range of 10^−9^–10^−5^ M, and as weak (I < 2) for **6b** at the concentration of 10^−5^ M. These compounds are direct mutagens. Substance **6c**, in addition, is also a promutagen. Its genotoxicity was registered under conditions of metabolic activation in the concentration range of 10^−7^–10^−6^ M. Compounds **6a**, **6d**, and **4** do not belong to the class of DNA-damaging substances.

Prooxidant characteristics (production of superoxide anion and NO) were evaluated using the biosensor *E. coli* MG1655 (pSoxS-lux) (Table 7). Compound **6b** did not possess prooxidant activity. A weak response was observed for compounds **6a** (10^−9^–10^−8^ M), **6d** (10^−6^ M), **6c** (10^−8^–10^−7^ M), and **6e** (10^−7^ M). On the other hand, for compound **4**, a significant effect of superoxide-anion radical or NO level increase was registered in a bacterial cell at the concentration of 1 × 10^−8^ M, and a weak effect was seen for concentrations 10^−9^–10^−8^ M and 10^−7^–10^−5^ M.

Taken together, the most promising candidates for further studies are compounds **6a** and **6d**, for which maximum suppression of bacterial biofilm growth was observed. These compounds were found to be non-genotoxic and possessed a weak pro-oxidant activity. However, a careful study of their biological activity in eukaryotic models is still required.

## 3. Materials and Methods

### 3.1. Chemistry

IR spectra were recorded on a UR-20 spectrometer in the 400–3600 cm^−1^ range in KBr. ^1^H-NMR spectra were recorded on a Bruker AVANCE 400 (400 MHz) spectrometer (Bruker BioSpin, Rheinstetten, Germany) with respect to the signals of residual protons of deuterated solvent (CDCl_3_, DMSO-d_6_). ^13^C-NMR spectra were recorded on a Bruker Avance 600 (151 MHz) spectrometer (Bruker BioSpin, Rheinstetten, Germany) relative to signals of residual protons of deuterated solvent (CDCl_3_, DMSO-d_6_). Elemental analysis was performed on a CHNS-O Elemental Analyser EuroEA3028-HT-OM (EuroVector S.p.A., Milan, Italy). The melting points were determined in glass capillaries on a Stuart SMP 10 instrument. *N*-(4,4-diethoxybutyl)ureas **1a**–**g** were obtained as previously described [38,46].

The X-ray diffraction data for crystal of compound **8f** were collected at 150 K on a Bruker AXS Smart Apex II CCD diffractometer in the ω and φ scan modes using graphite monochromated MoKα (λ 0.71073Å) radiation. The structure was solved by direct method and refined by the full matrix least-squares using the SHELXTL program [71]. All non-hydrogen atoms were refined anisotropically. The positions of hydrogen atoms were located from the Fourier electron density synthesis and were included in the refinement in the isotropic riding model approximation. All figures were made using OLEX2 [72] and Mercury [73]. Crystallographic data for the structure reported in this paper have been deposited with the Cambridge Crystallographic Data Center (1941912, www.ccdc.ac.uk).

*6-Chloro-4-((3-hydroxyphenyl)amino)-5-nitrobenzo[c][1,2,5]oxadiazole 1-oxide* (**4**). To the solution of benzofuroxan 3 (0.40 g, 1.6 mmol) in DMSO (3 mL) a solution of 3-aminophenol (0.35 g, 3.2 mmol) in DMSO (3 mL) was added at room temperature. The reaction mixture was stirred at room temperature for 2 h, reagents consumption was monitored by TLC (eluent: toluene/ethylacetate, 2/1). Then the reaction mixture was poured in water (100 mL), precipitate was filtered off, washed with water, and dried. Crude product was purified by column chromatography (eluent: toluene/ethylacetate, 2/1) and recrystallized from chloroform/hexane (3/1) to give target compound **4** as dark solid. Yield 93%, m.p. 128–130 °C; IR (ν, cm^−1^): 1563, 1628, 3094, 3320, 3447; ^1^H-NMR (400 MHz, CHCl_3_, δ ppm) 4.91 (s, 1H, NH), 6.73–6.75 (m, 1H, Ar-H), 6.80–6.84 (m, 2H, Ar-H), 6.92 (s, 1H, Ar-H), 7.27 (s, 1H, Ar-H), 8.49 (s, 1H, OH); ^13^C-NMR (151 MHz, CHCl_3_, δ ppm) 102.7, 111.8, 113.0, 114.8, 117.1, 128.4, 130.4, 130.6, 132.6, 138.8, 146.1, 156.3; Elemental analysis: calc. for C_12_H_7_ClN_4_O_5_ (322.5): C 44.67; H 2.19; Cl 10.99; N 17.36; found C 44.49; H 2.32; Cl 10.83; N 17.44. ESI *m/z*: [M + H]^+^: calc. for C_12_H_8_ClN_4_O_5_ 323; found 323.

*1-(4,4-Diethoxybutyl)-3-(2-(dimethylamino)ethyl)urea* (**1h**). To a solution of *N*^1^,*N*^1^-dimethylethane-1,2-diamine (0.97 g, 11.0 mmol) in dichloromethane (11 mL) 1,1’-carbonyldiimidazole (2.0 g, 12.3 mmol) was added. The reaction mixture was stirred for 48 hours at room temperature. Then 4,4-diethoxybutan-1-amine (1.77 g, 11.0 mmol) was added and reaction mixture was stirred for another 48 h at room temperature. The reaction mixture was extracted with water (3 × 10 mL), the organic layer was separated, and solvent was removed in vacuum to give target compound **1h** as yellow oil. Yield 64%; ^1^H-NMR (400 MHz, CHCl_3_, δ ppm) 1.10 (t, 6H, *J* = 7.1 Hz, CH_3_), 1.40–1.49 (m, 2H, CH_2_), 1.53–1.60 (m, 2H, CH_2_), 2.13 (s, 6H, CH_3_), 2.31 (t, 2H, *J* = 5.9 Hz, CH_2_), 3.04–3.11 (m, 2H, CH_2_), 3.12–3.20 (m, 2H, CH_2_), 3.36–3.45 (m, 2H, CH_2_), 3.51–3.60 (m, 2H, CH_2_), 4.39 (t, 1H, *J* = 5.6 Hz, CH), 5.37 (s, 1H,NH), 5.55 (s, 1H,NH); ^13^C-NMR (151 MHz, CHCl_3_, δ ppm) 15.2, 25.5, 31.1, 38.1, 40.0, 45.2, 59.3, 61.2, 102.8, 159.1.

General method for the synthesis of pyrrolidine-1-carboxamides **5**–**8**. To a mixture of appropriate *C*-nucleophile (1.61 mmol) and chloroform (5 mL), urea **1** (1.61 mmol) and trifluoroacetic acid (0.18 g, 1.61 mmol; 0.36 g, 3.22 mmol in the case of urea **1h**) were added. The reaction mixture was stirred for 24 h at room temperature, the solvent was removed in vacuum, and the residue was washed thoroughly with diethyl ether and dried in vacuum to give title compound.

*2-(6-Hydroxybenzo[d][1,3]dioxol-5-yl)pyrrolidine-1-carboxamide* (**5a**). Beige solid, yield 93%, m.p. 206–207 °C; IR (ν, cm^−1^): 1534, 1637, 2986, 3174, 3294; ^1^H-NMR (400 MHz, DMSO-*d*_6_, δ ppm) 1.69–1.77 (m, 1H, CH_2_), 1.78–1.88 (m, 2H, CH_2_), 2.06–2.16 (m, 1H, CH_2_), 3.29–3.36 (m, 1H, CH_2_), 3.46–3.52 (m, 1H, CH_2_), 4.93–4.99 (m, 1H, CH), 5.48–5.80 (br s, 2H, NH_2_), 5.86 (dd, 2H, *J* = 4.3 Hz, 1.0 Hz, CH_2_), ), 6.41 (s, 1H, Ar-H), 6.47 (s, 1H, Ar-H); 9.07–9.86 (br s, 1H, OH). ^13^C-NMR (151 MHz, DMSO-*d*_6_, δ ppm) 23.8, 33.2, 46.9, 55.3, 98.4, 100.9, 106.2, 122.9, 140.1, 146.3, 149.1, 157.9; Elemental analysis: calc. for C_12_H_14_N_2_O_4_ (250): C, 57.59; H, 5.64; N, 11.19; found C, 57.70; H, 5.71; N, 11.01; ESI *m/z*: [M + H]^+^: calc. for C_12_H_15_N_2_O_4_ 251; found 251.

*2-(6-Hydroxybenzo[d][1,3]dioxol-5-yl)-N-phenylpyrrolidine-1-carboxamide* (**5b**). Beige solid, yield 98%, m.p. 190–191 °C; IR (ν, cm^−1^): 1596, 1627, 2997, 3050; ^1^H-NMR (400 MHz, DMSO-*d*_6_, δ ppm) 1.72–1.91 (m, 3H, CH_2_), 2.12–2.22 (m, 1H, CH_2_), 3.48–3.56 (m, 1H, CH_2_), 3.72–3.80 (m, 1H, CH_2_), 5.14–5.20 (m, 1H, CH), 5.85 (s, 2H, CH_2_), 6.44 (s, 1H, Ar-H), 6.52 (s, 1H, Ar-H), 6.90 (t, 1H, *J* = 7.4 Hz, Ar-H), 7.20 (t, 2H, *J* = 7.8 Hz, Ar-H), 7.43 (d, 2H, *J* = 8.1 Hz, Ar-H), 7.97 (s, 1H,NH), 9.33 (s, 1H, OH); ^13^C-NMR (151 MHz, DMSO-*d*_6_, δ ppm) 23.7, 33.3, 47.1, 55.7, 98.2, 101.0, 106.1, 119.8, 122.1, 122.7, 128.7, 140.2, 140.9, 146.4, 148.8, 154.1; Elemental analysis: calc. for C_18_H_18_N_2_O_4_ (326): C, 66.25; H, 5.56; N, 8.58; found C, 66.31; H, 5.70; N, 8.35; ESI *m/z*: [M + H]^+^: calc. for C_18_H_19_N_2_O_4_ 327; found 327.

*2-(6-Hydroxybenzo[d][1,3]dioxol-5-yl)-N-(4-methoxyphenyl)pyrrolidine-1-carboxamide* (**5c**). Beige solid, yield 91%, m.p. 112–114 °C; IR (ν, cm^−1^): 1597, 1627, 2971, 2989, 3037; ^1^H-NMR (400 MHz, DMSO-*d*_6_, δ ppm) 1.72–1.92 (m, 3H, CH_2_), 2.10–2.27 (m, 1H, CH_2_), 3.45–3.56 (m, 2H, CH_2_), 3.68 (s, 3H, CH_3_), 5.12–5.22 (m, 1H, CH), 5.85 (s, 2H, CH_2_), 6.46 (s, 1H, Ar-H), 6.52 (s, 1H, Ar-H), 6.79 (d, 2H, *J* = 9.1 Hz, Ar-H), 7.33 (d, 2H, *J* = 9.0 Hz, Ar-H), 7.85 (s, 1H,NH), 9.39 (s, 1H, OH); ^13^C-NMR (151 MHz, DMSO-*d*_6_, δ ppm) 23.7, 33.2, 47.0, 55.6, 55.6, 98.3, 100.9, 106.2, 114.0, 121.8, 128. 8, 133.9, 140.2, 146.4, 148.9, 154.5, 154.9; Elemental analysis: calc. for C_19_H_20_N_2_O_5_ (356): C, 64.04; H, 5.66; N, 7.86; found C, 64.21; H, 5.87; N, 7.94; ESI *m/z*: [M + H]^+^: calc. for C_19_H_21_N_2_O_5_ 357; found 357.

*N-(4-Bromophenyl)-2-(6-hydroxybenzo[d][1,3]dioxol-5-yl)pyrrolidine-1-carboxamide* (**5d**). White solid, yield 95%, m.p. 164 °C; IR (ν, cm^−1^): 1595, 1627, 2848, 2978, 3047; ^1^H-NMR (400 MHz, DMSO-*d*_6_, δ ppm) 1.71–1.94 (m, 3H, CH_2_), 2.11–2.23 (m, 1H, CH_2_), 3.46–3.57 (m, 1H, CH_2_), 3.70–3.81 (m, 1H, CH_2_), 5.14–5.24 (m, 1H, CH), 5.85 (s, 2H, CH_2_), 6.44 (s, 1H, Ar-H), 6.49 (s, 1H, Ar-H), 7.37 (d, 2H, *J* = 8.8 Hz, Ar-H), 7.44 (d, 2H, *J* = 8.7 Hz, Ar-H), 8.17 (s, 1H,NH), 9.30 (s, 1H, OH); ^13^C-NMR (151 MHz, DMSO-*d*_6_, δ ppm) 23.7, 33.2, 47.1, 55.9, 98.2, 100.9, 106.1, 113.5, 121.6, 122.6, 131.5, 140.1, 140.4, 146.3, 148.8, 153.9; Elemental analysis: calc. for C_18_H_17_BrN_2_O_4_ (405): C, 53.35; H, 4.23; Br, 19.72; N, 6.91; found C, 53.41; H, 4.33; Br, 19.79; N, 6.79; ESI *m/z*: [M + H]^+^: calc. for C_12_H_8_ClN_4_O_5_ 323; found 323; ESI *m/z*: [M + H]^+^: calc. for C_18_H_18_BrN_2_O_4_ 406; found 406.

*N-(4-Fluorophenyl)-2-(6-hydroxybenzo[d][1,3]dioxol-5-yl)pyrrolidine-1-carboxamide* (**5e**). White solid, yield 91%, m.p. 184–185 °C; IR (ν, cm^−1^): 1595, 1638, 2883, 2948, 2989, 3164; ^1^H-NMR (400 MHz, DMSO-*d*_6_, δ ppm) 1.72–1.83 (m, 2H, CH_2_), 1.88–1.98 (m, 1H, CH_2_), 2.11–2.28 (m, 1H, CH_2_), 3.48–3.59 (m, 1H, CH_2_), 3.68–3.78 (m, 1H, CH_2_), 5.17–5.27 (m, 1H, CH), 6.57 (d, 2H, *J* = 7.9 Hz, CH_2_), 6.64 (s, 1H, Ar-H), 6.85 (d, 1H, *J* = 8.2 Hz, CH_2_), 6.99–7.07 (m, 2H, Ar-H), 7.33 (s, 1H, Ar-H), 7.40–7.48 (m, 2H, Ar-H), 8.09 (s, 1H, Ar-H), 9.72 (s, 1H,NH), 9.84 (s, 1H, OH); ^13^C-NMR (151 MHz, DMSO-*d*_6_, δ ppm) 23.6, 32.8, 47.1, 55.9, 102.2, 110.2, 113.7, 115.1 (d, *J* = 22.0 Hz), 121.6 (d, *J* = 7.6 Hz), 126.1, 130.5, 133.2, 137.8 (d, *J* = 124.5 Hz), 148.3, 154.2, 157.5 (d, *J* = 137.9 Hz); Elemental analysis: calc. for C_18_H_17_FN_2_O_4_ (344): C, 62.79; H, 4.98; N, 8.14; found C, 62.93; H, 5.09; N, 8.30; ESI *m/z*: [M + H]^+^: calc. for C_18_H_18_FN_2_O_4_ 345; found 345.

*N-Hexyl-2-(6-hydroxybenzo[d][1,3]dioxol-5-yl)pyrrolidine-1-carboxamide* (**5f**). White solid, yield 72%, m.p. 91–93 °C; IR (ν, cm^−1^): 1535, 1624, 2720, 2855, 2929, 3115; ^1^H-NMR (400 MHz, CDCl_3_, δ ppm) 0.87 (t, 3H, *J* = 6.9 Hz, CH_3_), 1.23–1.31 (m, 6H, CH_2_), 1.42–1.51 (m, 2H, CH_2_), 2.04–2.13 (m, 2H, CH_2_), 2.18–2.32 (m, 2H, CH_2_), 3.10–3.16 (m, 1H, CH_2_), 3.19–3.28 (m, 1H, CH_2_), 3.40–3.54 (m, 2H, CH_2_), 5.12–5.21 (m, 1H, CH), 5.85 (d, 2H, *J* = 12.4 Hz, CH_2_), 6.47 (s, 1H, Ar-H), 6.61 (s, 1H, Ar-H); ^13^C-NMR (151 MHz, CDCl_3_, δ ppm) 14.0, 22.6, 24.9, 26.5, 30.1, 31.5, 32.7, 40.9, 46.4, 55.0, 99.9, 100.9, 105.3, 120.8, 141.0, 147.3, 150.4, 158.0; Elemental analysis: calc. for C_18_H_26_N_2_O_4_ (334): C, 64.65; H, 7.84; N, 8.38; found C, 64.75; H, 8.06; N, 8.25; ESI *m/z*: [M + H]^+^: calc. for C_18_H_27_N_2_O_4_ 335; found 335.

*N-Cyclohexyl-2-(6-hydroxybenzo[d][1,3]dioxol-5-yl)pyrrolidine-1-carboxamide* (**5g**). White solid, yield 84%, m.p. 159–160 °C; IR (ν, cm^−1^): 1528, 1624, 2720, 2855, 2929, 3113, 3403; ^1^H-NMR (400 MHz, DMSO-*d*_6_, δ ppm) 1.03–1.12 (m, 2H, CH_2_), 1.13–1.25 (m, 3H, CH_2_), 1.47–1.66 (m, 4H, CH_2_), 1.69–1.78 (m, 2H, CH_2_), 1.79–1.90 (m, 2H, CH_2_), 2.07–2.19 (m, 1H, CH_2_), 3.31–3.43 (m, 2H, CH_2_), 3.47–3.54 (m, 1H, CH_2_), 4.95–5.04 (m, 1H, CH), 5.85 (d, 2H, *J* = 8.2 Hz, CH_2_), 6.42 (s, 1H, Ar-H), 6.47 (s, 1H, Ar-H); ^13^C-NMR (151 MHz, DMSO-*d*_6_, δ ppm) 23.9, 25.2, 25.8, 33.5, 33.6, 46.7, 49.1, 55.0, 98.3, 100.9, 106.2, 122.7, 140.3, 146.5, 149.1, 156.3; Elemental analysis: calc. for C_18_H_24_N_2_O_4_ (332): C, 65.04; H, 7.28; N, 8.43; found C, 65.16; H, 7.42; N, 8.35; ESI *m/z*: [M + H]^+^: calc. for C_18_H_25_N_2_O_4_ 333; found 333.

*2-(2-(6-Hydroxybenzo[d][1,3]dioxol-5-yl)pyrrolidine-1-carboxamido)-N,N-dimethylethan-1-aminium 2,2,2-trifluoroacetate* (**5h**). White solid, yield 75%, m.p. 171–172 °C; IR (ν, cm^−1^): 1527, 1626, 2838, 2929, 3113, 3394; ^1^H-NMR (400 MHz, DMSO-*d*_6_, δ ppm) 1.69–1.80 (m, 2H, CH_2_), 1.81–1.89 (m, 1H, CH_2_), 2.04–2.09 (m, 1H, CH_2_), 2.79 (s, 6H, CH_3_), 3.06–3.16 (m, 2H, CH_2_), 3.31–3.37 (m, 3H, CH_2_), 3.53–3.60 (m, 1H, CH_2_), 4.99–5.05 (m, 1H, CH), 5.85 (d, 2H, *J* = 11.0 Hz, CH_2_), 6.42 (s, 1H, Ar-H), 6.44 (s, 1H, Ar-H), 9.37 (s, 1H,NH), 9.63 (s, 1H, OH); ^13^C-NMR (151 MHz, DMSO-*d*_6_, δ ppm) 23.4, 33.2, 35.9, 43.1, 46.7, 55.9, 57.9, 98.3, 100.9, 106.1, 117.7 (q, *J* = 300.4 Hz), 122.8, 139.9, 146.2, 148.9, 157.1, 158.6 (q, *J* = 31.0 Hz); Elemental analysis: calc. for C_18_H_24_F_3_N_3_O_6_ (435): C, 49.66; H, 5.56; N, 9.65; found C, 49.85; H, 5.67; N, 9.80; ESI *m/z*: [M − CF_3_CO_2_]^+^: calc. for C_16_H_24_N_3_O_4_ 322; found 322.

*6-Chloro-4-((3-hydroxy-4-(1-(phenylcarbamoyl)pyrrolidin-2-yl)phenyl)amino)-5-nitrobenzo[c][1,2,5]oxadiazole 1-oxide* (**6b**). Dark solid, yield 87%, m.p. 165–170 °C with decomposition; IR (ν, cm^−1^): 753, 1383, 1559, 1627, 3073, 3336, 3396; ^1^H-NMR (400 MHz, DMSO-*d*_6_, δ ppm) 1.80 (m, 2H, CH_2_), 1.91 (m, 1H, CH_2_), 2.20 (m, 1H, CH_2_), 3.53–3.54 (m, 1H, CH_2_), 3.75 (m, 1H, CH_2_), 5.22–5.23 (m, 1H, CH), 6.57 (d, 1H, *J* = 7.7 Hz, Ar-H), 6.64 (s, 1H, Ar-H), 6.87 (d, 1H, *J* = 8.2 Hz, Ar-H), 6.91 (t, 1H, *J* = 7.40 Hz, Ar-H), 7.20 (t, 2H, *J* = 7.9 Hz, Ar-H), 7.33 (s, 1H, Ar-H), 7.43 (d, 2H, *J* = 7.1 Hz, Ar-H), 7.99 (s, 1H, NH), 9.74 (s, 1H, NH), 9.84 (s, 1H, OH); ^13^C-NMR (151 MHz, DMSO-*d*_6_, δ ppm) 23.7, 33.0, 47.1, 55.8, 102.3, 110.3, 113.9, 114.3, 119.9, 122.1, 126.2, 127.1, 128.1, 128.7, 130.6, 133.3, 138.5, 140.9, 148.3, 154.2, 154.4; Elemental analysis: calc. for C_23_H_19_ClN_6_O_6_ (510.9): C 54.07; H 3.75; Cl 6.94; N 16.45; found C 54.18; H 3.83; Cl 6.85; N 16.32; ESI *m/z*: [M + H]^+^: calc. for C_23_H_20_ClN_6_O_6_ 511.9; found 512.

*6-Chloro-4-((3-hydroxy-4-(1-((4-methoxyphenyl)carbamoyl)pyrrolidin-2-yl)phenyl)amino)-5-nitrobenzo[c][1,2,5]oxadiazole 1-oxide* (**6c**). Dark solid, yield 89%, m.p. 196–202 °C with decomposition; IR (ν, cm^−1^): 750, 1377, 1557, 1628, 3075, 3306, 3391; ^1^H-NMR (400 MHz, DMSO-*d*_6_, δ ppm) 1.73–1.84 (m, 2H, CH_2_), 1.86–1.94 (m, 1H, CH_2_), 2.12–2.23 (m, 1H, CH_2_), 3.47–3.53 (m, 1H, CH_2_), 3.69 (s, 3H, CH_3_), 3.69–3.74 (m, 1H, CH_2_), 5.16–5.23 (m, 1H, CH), 6.57 (d, 1H, *J* = 7.6 Hz, Ar-H), 6.63 (s, 1H, Ar-H), 6.78 (d, 2H, *J* = 9.02 Hz, Ar-H), 6.87 (d, 1H, *J* = 8.3 Hz, Ar-H), 7.31 (s, 1H, Ar-H), 7.33 (d, 2H, *J* = 4.8 Hz, Ar-H), 7.86 (br s, 1H, NH), 9.73 (br s, 1H, NH), 9.83 (nr s, 1H, OH); ^13^C-NMR (151 MHz, DMSO-*d*_6_, δ ppm) 23.7, 32.1, 32.9, 47.1, 55.6, 102.2, 107.0, 110.3, 114.0, 121.8, 121.9, 123.0, 124.5, 127.1, 130.6, 133.9, 138.5, 141.3, 146.7, 148.2, 154.5, 154.9; Elemental analysis: calc. for C_24_H_21_ClN_6_O_7_ (540.9): C 53.29; H 3.91; Cl 6.55; N 15.54; found C 53.38; H 3.81; Cl 6.42; N 15.67; ESI *m/z*: [M + H]^+^: calc. for C_24_H_22_ClN_6_O_7_ 541.9; found 542.

*4-((4-(1-((4-Bromophenyl)carbamoyl)pyrrolidin-2-yl)-3-hydroxyphenyl)amino)-6-chloro-5-nitrobenzo[c][1,2,5]oxadiazole 1-oxide* (**6d**). Dark solid, yield 95%, m.p. 166–170 °C with decomposition; IR (ν, cm^−1^): 753, 1362, 1560, 1629, 3100, 3297, 3382; ^1^H-NMR (400 MHz, DMSO-*d*_6_, δ ppm) 1.74–1.84 (m, 2H, CH_2_), 1.84–1.97 (m, 1H, CH_2_), 2.15–2.24 (m, 1H, CH_2_), 3.47–3.57 (m, 1H, CH_2_), 3.68–3.77 (m, 1H, CH_2_), 5.18–5.25 (m, 1H, CH), 6.56 (d, 1H, *J* = 8.2 Hz, Ar-H), 6.63 (s, 1H, Ar-H), 6.85 (d, 1H, *J* = 8.24 Hz, Ar-H), 7.33 (s, 1H, Ar-H), 7.36 (d, 2H, *J* = 8.8 Hz, Ar-H), 7.44 (t, 2H, *J* = 8.8 Hz, Ar-H), 8.19 (br s, 1H, NH), 9.71 (br s, 1H, NH), 9.83 (br s, 1H, OH); ^13^C-NMR (151 MHz, DMSO-*d*_6_, δ ppm) 23.6, 32.9, 47.1, 56.0, 110.2, 113.5, 113.8, 114.3, 120.8, 121.7, 126.1, 127.1, 130.6, 131.4, 132.0, 138.5, 139.5, 140.4, 148.3, 153.9, 154.4; Elemental analysis: calc. for C_23_H_18_BrClN_6_O_6_ (589.8): C 46.84; H 3.08; Cl 6.01; N 14.25; found C 46.92; H 3.12; Cl 6.05; N 14.34; ESI *m/z*: [M + H]^+^: calc. for C_23_H_19_BrClN_6_O_6_ 590.8; found 591.

*6-Chloro-4-((4-(1-((4-fluorophenyl)carbamoyl)pyrrolidin-2-yl)-3-hydroxyphenyl)amino)-5-nitrobenzo[c][1,2,5]oxadiazole 1-oxide* (**6e**). Dark solid, yield 76%, m.p. 175–177 °C with decomposition; IR (ν, cm^−1^): 1348, 1564, 1623, 3247, 3404; ^1^H-NMR (400 MHz, DMSO-*d*_6_, δ ppm) 1.65–1.83 (m, 2H, CH_2_), 1.86–1.98 (m, 1H, CH_2_), 2.11–2.27 (m, 1H, CH_2_), 3.49–3.58 (m, 1H, CH_2_), 3.69–3.79 (m, 1H, CH_2_), 5.16–5.25 (m, 1H, CH), 6.57 (dd, 1H, *J* = 8.2 Hz, *J* = 2.1 Hz, Ar-H), 6.64 (d, 1H, *J* = 2.1 Hz, Ar-H), 6.85 (d, 1H, *J* = 8.1 Hz, Ar-H), 6.98–7.07 (m, 2H, Ar-H), 7.33 (s, 1H, Ar-H), 7.41–7.49 (m, 2H, Ar-H), 8.09 (s, 1H, NH), 9.72 (s, 1H,NH), 9.84 (s, 1H, OH); ^13^C-NMR (151 MHz, DMSO-*d*_6_, δ ppm) 23.6, 32.8, 47.1, 55.9, 102.2, 110.2, 113.7, 114.2, 115.10 (d, *J* = 22.0 Hz), 121.6 (d, *J* = 7.6 Hz), 126.1, 127.1, 128.0, 130.5, 133.2, 137.2 (d, *J* = 2.1 Hz), 138.4, 148.3, 154.2, 157.7 (d, *J* = 237.9 Hz); Elemental analysis: calc. for C_23_H_18_ClFN_6_O_6_ (528.9): C, 52.23; H, 3.43; Cl, 6.70; N, 15.89; found C, 52.00; H, 3.61; Cl, 6.87; N, 16.07; ESI *m/z*: [M + H]^+^: calc. for C_23_H_19_ClFN_6_O_6_ 529.9; found 530.

*6-Chloro-4-((4-(1-(hexylcarbamoyl)pyrrolidin-2-yl)-3-hydroxyphenyl)amino)-5-nitrobenzo[c][1,2,5]oxadiazole 1-oxide* (**6f**). Dark solid, yield 44%, m.p. 121–122 °C with decomposition; IR (ν, cm^−1^): 722, 1347, 1563, 1630, 3106, 3194, 3426; ^1^H-NMR (400 MHz, DMSO-*d*_6_, δ ppm) 0.84 (t, 3H, *J* = 6.7 Hz, CH_3_), 1.17–1.26 (m, 6H, CH_2_), 1.31–1.38 (m, 2H, CH_2_), 1.71–1.79 (m, 2H, CH_2_), 1.80–1.90 (m, 1H, CH_2_), 2.07–2.18 (m, 1H, CH_2_), 2.90–3.02 (m, 2H, CH_2_), 3.42–3.54 (m, 2H, CH_2_), 4.99–5.06 (m, 1H, CH), 5.85 (br s, 1H, NH), 6.55 (d, 1H, *J* = 7.2 Hz, Ar-H), 6.61 (s, 1H, Ar-H), 6.79 (d, 1H, *J* = 8.3 Hz, Ar-H), 7.34 (s, 1H, Ar-H), 9.01 (br s, 1H, NH), 9.85 (br s, 1H, OH); ^13^C-NMR (151 MHz, DMSO-*d*_6_, δ ppm) 14.4, 22.5, 23.7, 26.5, 30.4, 31.5, 32.9, 46.7, 55.3, 102.3, 110.4, 113.7, 126.3, 127.1, 128.3, 130.6, 133.3, 134.9, 138.5, 148.3, 154.6, 157.0; Elemental analysis: calc. for C_23_H_27_ClN_6_O_6_ (518.9): C 53.23; H 5.24; Cl 6.83; N 16.19; found C 53.35; H 5.33; Cl 6.72; N 16.12; ESI *m/z*: [M + H]^+^: calc. for C_23_H_28_ClN_6_O_6_ 519.9; found 520.

*6-Chloro-4-((4-(1-(cyclohexylcarbamoyl)pyrrolidin-2-yl)-3-hydroxyphenyl)amino)-5-nitrobenzo[c][1,2,5]oxadiazole 1-oxide* (**6g**). Dark solid, yield 67%, m.p. 170–175 °C with decomposition; IR (ν, cm^−1^): 632, 1348, 1564, 1623, 2934, 3247, 3404; ^1^H-NMR (400 MHz, DMSO-*d*_6_, δ ppm) 0.99–1.23 (m, 6H, CH_2_), 1.50–1.79 (m, 7H, CH_2_), 2.30–2.35 (m, 1H, CH_2_), 2.64–2.69 (m, 1H, CH_2_), 3.27–3.33 (m, 1H, CH_2_), 3.33–3.39 (m, 1H, CH), 4.95–5.02 (m, 1H, CH), 6.57 (d, 1H, *J* = 8.05 Hz, Ar-H), 6.61 (s, 1H, Ar-H), 6.82 (d, 1H, *J* = 8.13 Hz, Ar-H), 7.31 (s, 1H, Ar-H), 7.93 (br s, 1H, NH), 9.83 (br s, 1H, NH), 9.94 (br s, 1H, OH). ^13^C-NMR (151 MHz, DMSO-*d*_6_, δ ppm) 23.8, 25.3, 25.8, 33.6, 46.8, 49.2, 55.0, 65.5, 102.3, 110.4, 114.0, 126.5, 127.1, 128.7, 129.4, 130.6, 133.4, 138.7, 148.3, 154.7, 156.3; Elemental analysis: calc. for C_23_H_25_ClN_6_O_6_ (516.9): C 53.44; H 4.87; Cl 6.86; N 16.26; found C 53.33; H 4.75; Cl 6.79; N 16.21; ESI *m/z*: [M + H]^+^: calc. for C_23_H_26_ClN_6_O_6_ 517.9; found 518.

*6-Chloro-4-((4-(1-((2-(dimethylammonio)ethyl)carbamoyl)pyrrolidin-2-yl)-3-hydroxyphenyl)amino)-5-nitrobenzo[c][1,2,5]oxadiazole 1-oxide 2,2,2-trifluoroacetate* (**6h**). Beige solid, yield 57%, m.p. 211–212 °C with decomposition; IR (ν, cm^−1^): 1349, 1566, 1623, 3175, 3275, 3400; ^1^H-NMR (400 MHz, DMSO-*d*_6_, δ ppm) 1.70–1.79 (m, 1H, CH_2_), 1.84–1.91 (m, 1H, CH_2_), 2.08–2.19 (m, 1H, CH_2_), 2.79 (s, 6H, CH_3_), 3.07–3.13 (m, 2H, CH_2_), 3.32–3.38 (m, 3H, CH_2_), 3.51–3.58 (m, 1H, CH_2_), 5.04–5.12 (m, 1H, CH), 6.54 (d, 1H, *J* = 7.9 Hz, Ar-H), 6.58 (s, 1H, Ar-H), 6.63 (s, 1H, Ar-H), 7.35 (s, 1H, NH), 9.76 (s, 1H,NH), 9.83 (s, 1H, OH); ^13^C-NMR (151 MHz, DMSO-*d*_6_, δ ppm) 23.3, 32.9, 36.0, 43.1, 46.7, 55.9, 58.0, 102.3, 110.1, 113.5, 114.3, 114.7, 117.7 (q, *J* = 300.3 Hz), 126.1, 127.1, 130.5, 133.4, 138.5, 140.3, 148.3, 154.5, 157.1, 158.2, 158.5 (q, *J* = 31.0 Hz); Elemental analysis: calc. for C_23_H_25_ClF_3_N_7_O_8_ (619.9): C, 44.56; H, 4.06; Cl, 5.72; N, 15.82; found C, 44.79; H, 3.81; Cl, 5.87; N, 15.99; ESI *m/z*: [M − CF_3_CO_2_]^+^: calc. for C_21_H_25_ClN_7_O_6_ 506.9; found 507.

*2-(4-Hydroxy-6-methyl-2-oxo-2H-pyran-3-yl)-N-phenylpyrrolidine-1-carboxamide* (**7b**). White solid, yield 69%, m.p. 190–191 °C; IR (ν, cm^−1^): 1588, 1612, 1692, 2630, 2687, 2872, 2961; ^1^H-NMR (400 MHz, DMSO-*d*_6_, δ ppm) 1.76–1.87 (m, 1H, CH_2_), 1.95–2.06 (m, 2H, CH_2_), 2.08–2.21 (m, 1H, CH_2_), 2.13 (s, 3H, CH_3_), 3.50–3.65 (m, 2H, CH_2_), 4.99-5.09 (m, 1H, CH), 5.98 (s, 1H, Ar-H), 6.88 (t, 1H, *J* = 7.3 Hz, Ar-H), 7.18 (t, 2H, *J* = 7.8 Hz, Ar-H), 7.42 (d, 2H, *J* = 8.1 Hz, Ar-H), 7.81 (s, 1H, NH), 11.54 (s, 1H, OH); ^13^C-NMR (151 MHz, DMSO-*d*_6_, δ ppm) 19.7, 25.4, 30.8, 47.4, 52.1, 100.7, 118.1, 119.4, 121.8, 128.7, 141.1, 153.6, 161.3, 163.8, 166.1; Elemental analysis: calc. for C_17_H_18_N_2_O_4_ (314): C, 64.96; H, 5.77; N, 8.91; found C, 65.22; H, 5.89; N, 8.80; ESI *m/z*: [M + H]^+^: calc. for C_17_H_19_N_2_O_4_ 315; found 315.

*2-(4-Hydroxy-6-methyl-2-oxo-2H-pyran-3-yl)-N-(4-methoxyphenyl)pyrrolidine-1-carboxamide* (**7c**). White solid, yield 87%, m.p. 168–169 °C; IR (ν, cm^−1^): 1579, 1612, 1690, 2631, 2678, 2872, 3006; ^1^H-NMR (400 MHz, DMSO-*d*_6_, δ ppm) 1.75–1.86 (m, 1H, CH_2_), 1.97–2.18 (m, 3H, CH_2_), 2.13 (s, 3H, CH_3_), 3.46–3.59 (m, 2H, CH_2_), 3.68 (s, 3H, CH_3_), 4.96–5.03 (m, 1H, CH), 5.97 (s, 1H, Ar-H), 6.78 (d, 2H, *J* = 8.8 Hz, Ar-H), 7.31 (d, 2H, *J* = 9.1 Hz, Ar-H), 7.69 (s, 1H, NH), 11.54 (s, 1H, OH); ^13^C-NMR (151 MHz, DMSO-*d*_6_, δ ppm) 19.7, 25.4, 30.7, 47.3, 52.1, 55.6, 100.7, 102.9, 114.0, 121.2, 134.1, 154.0, 154.7, 161.3, 163.8, 166.2; Elemental analysis: calc. for C_18_H_20_N_2_O_5_ (344): C, 62.78; H, 5.85; N, 8.13; found C, 62.89; H, 5.98; N, 8.06; ESI *m/z*: [M + H]^+^: calc. for C_18_H_21_N_2_O_5_ 345; found 315.

*N-(4-Bromophenyl)-2-(4-hydroxy-6-methyl-2-oxo-2H-pyran-3-yl)pyrrolidine-1-carboxamide* (**7d**). White solid, yield 75%, m.p. 194–195 °C; IR (ν, cm^−1^): 1578, 1612, 1679, 2654, 2815, 2877, 2989; ^1^H-NMR (400 MHz, DMSO-*d*_6_, δ ppm) 1.76–1.86 (m, 1H, CH_2_), 1.92–2.04 (m, 2H, CH_2_), 2.06–2.17 (m, 1H, CH_2_), 2.12 (s, 3H, CH_3_), 3.50–3.61 (m, 2H, CH_2_), 4.97–5.05 (m, 1H, CH), 5.96 (s, 1H, Ar-H), 7.35 (d, 2H, *J* = 8.7 Hz, Ar-H), 7.43 (d, 2H, *J* = 8.5 Hz, Ar-H), 8.03 (s, 1H, NH), 11.42 (s, 1H, OH); ^13^C-NMR (151 MHz, DMSO-*d*_6_, δ ppm) 19.7, 25.4, 30.8, 47.4, 52.3, 100.7, 102.7, 113.1, 121.2, 131.5, 140.6, 153.3, 161.2, 163.7, 166.0; Elemental analysis: calc. for C_17_H_17_BrN_2_O_4_ (392): C, 51.92; H, 4.36; Br, 20.32; N, 7.12; found C, 52.09; H, 4.50; Br, 20.48; N, 7.31; ESI *m/z*: [M + H]^+^: calc. for C_17_H_18_BrN_2_O_4_ 393; found 393.

*N-(4-Fluorophenyl)-2-(4-hydroxy-6-methyl-2-oxo-2H-pyran-3-yl)pyrrolidine-1-carboxamide* (**7e**). White solid, yield 58%, m.p. 177–178 °C; IR (ν, cm^−1^): 1602, 1631, 1689, 2631, 2689, 2881, 3066; ^1^H-NMR (400 MHz, DMSO-*d*_6_, δ ppm) 1.74–1.87 (m, 1H, CH_2_), 1.91–2.14 (m, 3H, CH_2_), 2.12 (s, 3H, CH_3_), 3.50–3.63 (m, 2H, CH_2_), 4.95–5.07 (m, 1H, CH), 5.97 (s, 1H, Ar-H), 6.98–7.04 (m, 2H, Ar-H), 7.39–7.49 (m, 2H, Ar-H), 7.92 (s, 1H, NH), 11.62 (s, 1H, OH); ^13^C-NMR (151 MHz, DMSO-*d*_6_, δ ppm) 19.7, 25.4, 30.8, 47.3, 52.2, 100.6, 102.7, 115.1 (d, *J* = 22.0 Hz), 121.0 (d, *J* = 7.4 Hz), 137.4 (d, *J* = 2.5 Hz), 153.6, 157.5 (d, *J* = 237.6 Hz), 161.2, 163.7, 166.0; Elemental analysis: calc. for C_17_H_17_FN_2_O_4_ (332): C, 61.44; H, 5.16; N, 8.43; found 61.55; H, 4.89; N, 8.27; ESI *m/z*: [M + H]^+^: calc. for C_17_H_18_FN_2_O_4_ 333; found 333.

*N-Hexyl-2-(4-hydroxy-6-methyl-2-oxo-2H-pyran-3-yl)pyrrolidine-1-carboxamide* (**7f**). White solid, yield 47%, m.p. 137–138 °C; IR (ν, cm^−1^): 1592, 1690, 2631, 2686, 2935, 3079; ^1^H-NMR (400 MHz, DMSO-*d*_6_, δ ppm) 0.84 (t, 3H, *J* = 7.0 Hz, CH_3_), 1.16–1.27 (m, 6H, CH_2_), 1.30–1.38 (m, 2H, CH_2_), 1.70–1.80 (m, 1H, CH_2_), 1.95–2.10 (m, 3H, CH_2_), 2.13 (s, 3H, CH_3_), 2.87–2.96 (m, 1H, CH_2_), 2.98–3.07 (m, 1H, CH_2_), 3.31–3.35 (m, 1H, CH_2_), 3.36–3.41 (m, 1H, CH_2_), 4.80–4.88 (m, 1H, CH), 5.70 (s, 1H, NH), 5.96 (s, 1H, Ar-H); ^13^C-NMR (151 MHz, DMSO-*d*_6_, δ ppm) 14.4, 19.7, 22.5, 25.3, 26.5, 30.3, 30.4, 31.5, 35.6, 47.0, 51.9, 101.0, 103.0, 156.9, 161.4, 163.5, 167.0; Elemental analysis: calc. for C_17_H_26_N_2_O_4_ (322): C, 63.33; H, 8.13; N, 8.69; found C, 63.50; H, 8.31; N, 8.87; ESI *m/z*: [M + H]^+^: calc. for C_17_H_27_N_2_O_4_ 323; found 323.

*N-Cyclohexyl-2-(4-hydroxy-6-methyl-2-oxo-2H-pyran-3-yl)pyrrolidine-1-carboxamide* (**7g**). Beige solid, yield 60%, m.p. 184–185 °C; IR (ν, cm^−1^): 1593, 1692, 2631, 2686, 2934, 3079; ^1^H-NMR (400 MHz, DMSO-*d*_6_, δ ppm) 0.99–1.14 (m, 3H, CH_2_), 1.16–1.28 (m, 2H, CH_2_), 1.48–1.54 (m, 1H, CH_2_), 1.55–1.66 (m, 3H, CH_2_), 1.69–1.77 (m, 2H, CH_2_), 1.99–2.09 (m, 3H, CH_2_), 2.12–2.22 (m, 1H, CH_2_), 2.14 (s, 3H, CH_3_), 3.36–3.39 (m, 1H, CH_2_), 3.40–3.47 (m, 1H, CH_2_), 4.78–4.87 (m, 1H, CH), 5.36 (s, 1H, NH), 5.97 (s, 1H, Ar-H), 12.01 (s, 1H, OH); ^13^C-NMR (151 MHz, DMSO-*d*_6_, δ ppm) 19.7, 25.1, 25.3, 25.8, 30.3, 33.6, 47.1, 49.1, 51.6, 100.8, 102.8, 156.1, 161.7, 163.6, 167.0; Elemental analysis: calc. for C_17_H_24_N_2_O_4_ (320): C, 63.73; H, 7.55; N, 8.74; found C, 63.87; H, 7.76; N, 8.59; ESI *m/z*: [M + H]^+^: calc. for C_17_H_25_N_2_O_4_ 321; found 321.

*2-(2-(4-Hydroxy-6-methyl-2-oxo-2H-pyran-3-yl)pyrrolidine-1-carboxamido)-N,N-dimethylethan-1-aminium 2,2,2-trifluoroacetate* (**7h**). White solid, yield 77%, m.p. 146–147 °C; IR (ν, cm^−1^): 1592, 1692, 2683, 2985, 3064; ^1^H-NMR (400 MHz, DMSO-*d*_6_, δ ppm) 1.73–1.81 (m, 1H, CH_2_), 1.83–1.90 (m, 1H, CH_2_), 1.96–2.08 (m, 2H, CH_2_), 2.12 (s, 3H, CH_3_), 2.78 (s, 6H, CH_3_), 3.05–3.12 (m, 2H, CH_2_), 3.21–3.30 (m, 1H, CH_2_), 3.32–3.39 (m, 3H, CH_2_), 4.85–4.94 (m, 1H, CH), 5.98 (s, 1H, Ar-H), 6.22 (s, 1H, NH), 9.56 (s, 1H, OH), 11.70 (s, 1H, NH^+^); ^13^C-NMR (151 MHz, DMSO-*d*_6_, δ ppm) 19.7, 25.2, 31.3, 36.0, 43.1, 46.9, 52.4, 58.1, 100.8, 102.9, 177.7 (q, *J* = 31.2 Hz), 156.8, 158.6 (q, *J* = 299.4 Hz), 160.9, 163.6, 166.2; Elemental analysis: calc. for C_17_H_24_F_3_N_3_O_6_ (423): C, 48.23; H, 5.71; N, 9.92; found C, 48.30; H, 5.85; N, 10.14; ESI *m/z*: [M − CF_3_CO_2_]^+^: calc. for C_15_H_24_N_3_O_4_ 310; found 310.

*2-(4-Hydroxy-2-oxo-2H-chromen-3-yl)-N-phenylpyrrolidine-1-carboxamide* (**8b**). Beige solid, yield 51%, m.p. 179–180 °C; IR (ν, cm^−1^): 1595, 1616, 1695, 2853, 2930, 3075. ^1^H-NMR (400 MHz, DMSO-*d*_6_, δ ppm) 1.87–1.98 (m, 1H, CH_2_), 2.13–2.27 (m, 3H, CH_2_), 3.64–3.71 (m, 2H, CH_2_), 5.23–5.29 (m, 1H, CH), 6.91 (t, 1H, *J* = 7.4 Hz, Ar-H), 7.19 (t, 3H, *J* = 7.9 Hz, Ar-H), 7.32–7.37 (m, 2H, Ar-H, NH), 7.42 (d, 2H, *J* = 8.0 Hz, Ar-H), 7.59 (t, 1H, *J* = 8.0 Hz, Ar-H), 7.94 (d, 1H, *J* = 7.9 Hz, Ar-H), 8.26 (s, 1H, OH); ^13^C-NMR (151 MHz, DMSO-*d*_6_, δ ppm) 25.8, 29.6, 47.8, 53.2, 106.5, 116.5, 118.1, 120.2, 122.3, 124.0, 124.3, 128.7, 132.5, 140.6, 152.7, 154.9, 161.3, 162.2; Elemental analysis: calc. for C_20_H_18_N_2_O_4_ (350): C, 68.56; H, 5.18; N, 8.00; found C, 68.70; H, 5.40; N, 7.89; ESI *m/z*: [M + H]^+^: calc. for C_20_H_19_N_2_O_4_ 351; found 351.

*2-(4-Hydroxy-2-oxo-2H-chromen-3-yl)-N-(4-methoxyphenyl)pyrrolidine-1-carboxamide* (**8c**). Beige solid, yield 45%, m.p. 152–153 °C; IR (ν, cm^−1^): 1596, 1617, 1697, 2847, 2984, 3036; ^1^H-NMR (400 MHz, DMSO-*d*_6_, δ ppm) 1.81–2.00 (m, 1H, CH_2_), 2.12–2.23 (m, 2H, CH_2_), 2.24–2.34 (m, 1H, CH_2_), 3.59–3.66 (m, 2H, CH_2_), 3.69 (s, 3H, CH_3_), 5.18–5.28 (m, 1H, CH), 6.79 (d, 2H, *J* = 9.1 Hz, Ar-H), 7.25–7.38 (m, 5H, Ar-H, NH), 7.59 (t, 1H, *J* = 7.0 Hz, Ar-H), 7.92 (d, 1H, *J* = 6.7 Hz, Ar-H), 8.19 (s, 1H, OH); ^13^C-NMR (151 MHz, DMSO-*d*_6_, δ ppm) 25.8, 29.4, 47.7, 53.2, 55.6, 106.5, 114.0, 116.4, 120.4, 122.3, 123.9, 124.3, 132.5, 133.3, 152.7, 155.1, 155.5, 161.2, 162.6; Elemental analysis: calc. for C_21_H_20_N_2_O_5_ (380): C, 66.53; H, 5.50; N, 7.49; found C, 66.53; H, 5.50; N, 7.49; ESI *m/z*: [M + H]^+^: calc. for C_21_H_21_N_2_O_5_ 381; found 381.

*N-(4-Bromophenyl)-2-(4-hydroxy-2-oxo-2H-chromen-3-yl)pyrrolidine-1-carboxamide* (**8d**). Beige solid, yield 68%, m.p. 179 °C; IR (ν, cm^−1^): 1595, 1617, 2797, 2837, 2987, 3078; ^1^H-NMR (400 MHz, DMSO-*d*_6_, δ ppm) 1.83–1.86 (m, 1H, CH_2_), 2.08–2.27 (m, 3H, CH_2_), 3.62–3.72 (m, 2H, CH_2_), 5.23–5.31 (m, 1H, CH), 7.30–7.40 (m, 5H, Ar-H, NH), 7.43 (d, 2H, *J* = 8.90 Hz), 7.59 (t, 1H, *J* = 8.2 Hz, Ar-H), 7.94 (d, 1H, *J* = 8.3 Hz, Ar-H), 8.39 (s, 1H, OH); ^13^C-NMR (151 MHz, DMSO-*d*_6_, δ ppm) 25.8, 29.8, 47.8, 53.2, 106.5, 113.6, 116.5, 120.0, 121.8, 123.9, 124.3, 131.5, 132.4, 140.2, 152.7, 154.3, 161.3, 161.9; Elemental analysis: calc. for C_20_H_17_BrN_2_O_4_ (429): C, 55.96; H, 3.99; Br, 18.61; N, 6.53; found C, 56.14; H, 4.18; Br, 18.73; N, 6.70; ESI *m/z*: [M + H]^+^: calc. for C_20_H_18_BrN_2_O_4_ 430; found 430.

*N-(4-Fluorophenyl)-2-(4-hydroxy-2-oxo-2H-chromen-3-yl)pyrrolidine-1-carboxamide* (**8e**). Beige solid, yield 51%, m.p. 165 °C; IR (ν, cm^−1^): 1589, 1624, 1697, 2847, 2983, 3036, 3106; ^1^H-NMR (400 MHz, DMSO-*d*_6_, δ ppm) 1.92–1.98 (m, 1H, CH_2_), 1.12–1.28 (m, 3H, CH_2_), 3.61–3.70 (m, 2H, CH_2_), 5.22–5.29 (m, 1H, CH), 6.99–7.05 (m, 2H, Ar-H), 7.29–7.40 (m, 3H, Ar-H, NH), 7.40–7.47 (m, 2H, Ar-H), 7.59 (t, 1H, *J* = 8.5 Hz, Ar-H), 7.93 (d, 1H, *J* = 6.9 Hz, Ar-H), 8.33 (s, 1H, OH); ^13^C-NMR (151 MHz, DMSO-*d*_6_, δ ppm) 25.7, 29.6, 47.7, 53.1, 106.5, 115.2 (d, *J* = 22.0 Hz), 116.4, 117.0, 121.9 (d, *J* = 7.7 Hz), 123.9, 124.2, 132.4, 136.9 (d, *J* = 2.5 Hz), 152.7, 154.8, 157.8 (d, *J* = 138.1 Hz), 161.3, 162.2; Elemental analysis: calc. for C_20_H_17_FN_2_O_4_ (368): C, 65.21; H, 4.65; N, 7.60; found C, 65.42; H, 4.78; N, 7.83; ESI *m/z*: [M + H]^+^: calc. for C_20_H_18_FN_2_O_4_ 369; found 369.

*N-Hexyl-2-(4-hydroxy-2-oxo-2H-chromen-3-yl)pyrrolidine-1-carboxamide* (**8f**). Beige solid, yield 34%, m.p. 124-125 °C; IR (ν, cm^−1^): 1554, 1614, 1687, 2858, 2929, 2953, 3075, 3374; ^1^H-NMR (400 MHz, DMSO-*d*_6_, δ ppm) 0.79 (t, 3H, *J* = 6.8 Hz, CH_3_), 1.19–1.22 (m, 5H, CH_2_), 1.30–1.35 (m, 1H, CH_2_), 1.37–1.41 (m, 1H, CH_2_), 1.85–1.95 (m, 1H, CH_2_), 1.07–1.16 (m, 1H, CH_2_), 2.20–2.17 (m, 1H, CH_2_), 2.40–2.48 (m, 1H, CH_2_), 2.94–3.02 (m, 2H, CH_2_), 3.03–3.10 (m, 1H, CH_2_), 3.31–3.39 (m, 1H, CH_2_), 3.41–3.49 (m, 1H, CH_2_), 5.06–5.14 (m, 1H, CH), 6.57 (s, 1H, NH); 7.29–7.33 (m, 2H, Ar-H), 7.55–7.60 (m, 1H, Ar-H), 7.87 (d, 1H, *J* = 7.8 Hz, Ar-H); ^13^C-NMR (151 MHz, DMSO-*d*_6_, δ ppm) 14.3, 22.5, 25.7, 26.5, 28.8, 30.1, 31.5, 40.7, 47.4, 53.4, 106.3, 116.3, 117.2, 124.1, 124.2, 132.6, 153.0, 158.8, 161.1, 164.5; Elemental analysis: calc. for C_20_H_26_N_2_O_4_ (358): C, 67.02; H, 7.31; N, 7.82; found C, 67.29; H, 7.55; N, 7.99; ESI *m/z*: [M + H]^+^: calc. for C_20_H_27_N_2_O_4_ 359; found 359.

Crystal data: C_20_H_26_N_2_O_4_, M = 358.43, colorless crystal 0.12 × 0.15 × 0.15 mm^3^, triclinic, space group P-1, Z = 6, a = 12.9336(12), b = 14.2803(13), c = 16.0108(14) Å, α = 70.825(2), β = 84.411(2), γ = 87.932(2)°, V = 2779.8(4) Å^3^, ρ_calc_ = 1.285 g/cm^3^, μ = 0.9 mm^−1^, 33,903 reflections collected (±h, ±k, ±l), 14,791 independent (Rint 0.0849) and 7433 observed reflections [I ≥ 2***σ***(*I*)], 703 refined parameters, R = 0.0673, *wR*_2_ = 0.1723, max. residual electron density was 0.667 (−0.528) eÅ^−3^.

*N-Cyclohexyl-2-(4-hydroxy-2-oxo-2H-chromen-3-yl)pyrrolidine-1-carboxamide* (**8g**). Beige solid, yield 68%, m.p. 166–167 °C; IR (ν, cm^−1^): 1549, 1616, 1694, 2475, 2853, 2935, 3075, 3373; ^1^H-NMR (400 MHz, DMSO-*d*_6_, δ ppm) 1.01–1.10 (m, 1H, CH_2_), 1.14–1.26 (m, 4H, CH_2_), 1.48–1.56 (m, 1H, CH_2_), 1.59–1.67 (m, 2H, CH_2_), 1.68–1.78 (m, 2H, CH_2_), 1.84–1.94 (m, 1H, CH_2_), 2.06–2.16 (m, 1H, CH_2_), 2.20–2.29 (m, 1H, CH_2_), 2.39–2.48 (m, 1H, CH_2_), 3.35–3.50 (m, 3H, CH_2_, CH), 5.04–5.13 (m, 1H, CH), 6.26 (s, 1H, OH), 7.30–7.35 (m, 2H, Ar-H), 7.59 (td, 1H, *J* = 7.8 Hz, *J* = 1.6 Hz, Ar-H) 7.88 (dd, 1H, *J* = 8.3 Hz, *J* = 1.6 Hz, Ar-H); ^13^C-NMR (151 MHz, DMSO-*d*_6_, δ ppm) 25.4, 25.7, 28.7, 33.3, 33.5, 47.5, 49.9, 53.4, 106.2, 116.3, 117.2, 124.1, 124.2, 132.6, 153.0, 158.1, 161.1, 164.5; Elemental analysis: calc. for C_20_H_24_N_2_O_4_ (356): C, 67.40; H, 6.79; N, 7.86; found C, 67.54; H, 6.89; N, 7.73; ESI *m/z*: [M + H]^+^: calc. for C_20_H_25_N_2_O_4_ 357; found 357.

*2-(2-(4-Hydroxy-2-oxo-2H-chromen-3-yl)pyrrolidine-1-carboxamido)-N,N-dimethylethan-1-aminium 2,2,2-trifluoroacetate* (**8h**). Beige solid, yield 73%, m.p. 147–148 °C; IR (ν, cm^−1^): 1544, 1615, 1684, 2718, 2876, 2957, 3038, 3368; ^1^H-NMR (400 MHz, DMSO-*d*_6_, δ ppm) 1.83–1.94 (m, 1H, CH_2_), 2.08–2.22 (m, 2H, CH_2_), 2.24–2.35 (m, 1H, CH_2_), 2.78 (s, 6H, CH_3_), 3.06–3.15 (m, 2H, CH_2_), 3.26–3.33 (m, 1H, CH_2_), 3.35–3.41 (m, 1H, CH_2_), 3.43–3.49 (m, 2H, CH_2_), 5.08–5.16 (m, 1H, CH), 6.75 (s, 1H, NH), 7.29–7.38 (m, 2H, Ar-H), 7.59 (t, 1H, *J* = 8.2 Hz, Ar-H) 7.92 (d, 1H, *J* = 7.9 Hz, Ar-H) 9.62 (s, 1H, OH); ^13^C-NMR (151 MHz, DMSO-*d*_6_, δ ppm) 25.6, 29.7, 36.0, 43.0, 47.2, 53.3, 57.4, 106.2, 116.2, 116.9 (q, *J* = 199.9 Hz), 117.4, 124.1, 124.2, 132.4, 152.9, 157.9, 158.7 (q, *J* = 32.1 Hz), 161.3, 163.5; Elemental analysis: calc. for C_20_H_24_F_3_N_3_O_6_ (458): C, 52.29; H, 5.27; N, 9.15; found C, 52.48; H, 5.16; N, 8.97; ESI *m/z*: [M + H]^+^: calc. for C_18_H_24_N_3_O_4_ 346; found 346.

### 3.2. Biological Studies

#### 3.2.1. In Vitro Studies of Anti-Cancer Activity

Cytotoxicity assay. Cytotoxic effects of the test compounds on human cancer and normal cells were estimated by means of the multifunctional Cytell Cell Imaging system (GE Health Care Life Science, Sweden) using the Cell Viability Bio App which precisely counts the number of cells and evaluates their viability from fluorescence intensity data. Two fluorescent dyes that selectively penetrate the cell membranes and fluoresce at different wavelengths were used in the experiments. A low-molecular-weight 4′,6-diamidin-2-phenylindol dye (DAPI) is able to penetrate the intact membranes of living cells and color nuclei in blue. The high-molecular-weight propidium iodide dye penetrates only dead cells with damaged membranes, staining them in yellow. As a result, living cells are painted in blue and dead cells are painted in yellow. DAPI and propidium iodide were purchased from Sigma. The M-Hela clone 11 human, epithelioid cervical carcinoma, strain of Hela, clone of M-Hela from the Type Culture Collection of the Institute of Cytology (Russian Academy of Sciences) and Chang liver cell line (Human liver cells) from N. F. Gamaleya Research Center of Epidemiology and Microbiology were used in the experiments. The cells were cultured in a standard Eagle’s nutrient medium manufactured at the Chumakov Institute of Poliomyelitis and Virus Encephalitis (PanEco company) and supplemented with 10% fetal calf serum and 1% nonessential amino acids. The cells were plated into a 96-well plate (Eppendorf) at a concentration of 100,000 cells/mL, 150 μL of medium per well, and cultured in a CO_2_ incubator at 37 °C. Twenty-four hours after seeding the cells into wells, the compound under study was added at a preset dilution, 150 μL to each well. The dilutions of the compounds at concentrations of 1–100 µM were prepared immediately in nutrient media; 5% DMSO (which does not induce the inhibition of cells at this concentration) was added for better solubility. The experiments were repeated three times. Intact cells cultured in parallel with experimental cells were used as a control.

Induction of Apoptotic Effects by test compounds. Cell Culture. M-Hela cells at 1 × 10^6^ cells/well in a final volume of 2 mL were seeded into six-well plates. After 24 hours of incubation, a solution of the test compound **6g** was added to the wells at the concentration studied.

Cytell Cell Imaging System Assay. M-Hela cells were plated into a 24-well plate (Eppendorf) at a concentration of 1 × 10^6^ cells/mL, 500 µL of medium per well, and cultured in a CO_2_ incubator at 37 °C. Twenty-four hours after seeding the cells into wells the compound was added at a preset dilution, 500 µL to each well. The dilutions of compound **6g** were prepared immediately in nutrient media; 5% DMSO (which did not induce the inhibition of cells at this concentration) was added for better solubility. Evaluation of apoptotic effects was performed with the help of multifunctional system Cytell Cell Imaging, using Cell Viability BioApp and Automated Imaging BioApp applications. The annexin V-Alexa Fluor 647 apoptosis detection kit, DAPI and propidium iodide purchased from Sigma.

Multiplex analysis of early apoptosis markers. M-Hela cells were incubated for 24 hours with the test substance. Cells were lysed in MILLIPLEX^®^ MAP Lysis buffer containing protease inhibitors. Twenty micrograms of total protein of each lysate diluted in MILLIPLEX^®^ MAP Assay Buffer 2 was analyzed according to the analysis protocol (the lysate was incubated at 4 °C overnight). The mean fluorescence intensity (MFI) was detected using the Luminex^®^ system, MERCK, USA.

Flow Cytometry Assay. Mitochondrial membrane potential. Cells were harvested at 2000 rpm for 5 min and then washed twice with ice-cold PBS, followed by resuspension in JC-10 (10 µg/mL) and incubation at 37 °C for 10 min. After the cells were rinsed three times and suspended in PBS, the JC-10 fluorescence was observed by flow cytometry (Guava easy Cyte 8HT, Guava Technologies Inc., Hayward, CA, USA).

Statistical analysis. The experiments were repeated three times. The cytometric results were analyzed by the Cytell Cell Imaging multifunctional system using the Cell Viability BioApp and Apoptosis BioApp application. The data in the tables and graphs are given as the mean ± standard error.

#### 3.2.2. In Vivo Studies of Anti-Cancer Activity

Animals. In vivo experiments were performed using the BDF_1_ hybrid male mice of 22–24 g weight. The experimental animals were caged in a standard vivarium in 12 h light conditions with free access to food and water. All manipulations with the animals were performed in accordance with the solutions of the Commission on Bioethics of the Institute of Problems of Chemical Physics, Russian Academy of Sciences (IPCP RAS). 

Anti-tumor activity. The tumors were transplanted intraperitoneally (i.p.) in accordance with a standard procedure inoculum: 10^6^ tumor cells in isotonic solution of NaCl, V = 0.2 cm^3^ (leukemia P388) [60]. Original compounds were injected intraperitoneally as aqueous solution. Doses from 18 to 83 mg/kg/day and the mode of administration on days 1, 5, and 9 after transplantation were used. In each experiment, a single group of tumor-bearing animals not injected with the compounds served as the control group. Each group consisted of six mice. The animals were observed daily for survival for a minimum of 60 days. The efficacy of the therapy against leukemia (defined as increase in lifespan—ILS) was assessed as the percentage of the median survival time (MST) of the treated group (t) to that of the control group (c): ILS(%) = (MSTt/MSTc) × 100.

Statistics. The experiments were carried out in triplicate. The data are presented in the form X ± SD (mean ± standard deviation). The significance of the differences between the groups was assessed using Student’s *t*-test. Values of *p* < 0.05 were considered statistically significant. The data were processed statistically using GraphPad Prism.

#### 3.2.3. Bacterial Biofilm Formation Inhibitory Activity

Bacterial strains and cultivation conditions. For the detection of biofilms, formation strains *Vibrio aquamarinus* DSM 26054 and *Acinetobacter calcoaceticus* VKPM B-10353 were used. These strains form biofilms, making them useful for studying biofilms.

*E. coli* MG1655 (pRecA-lux) was used for the evaluation of the genotoxicity of the synthesized compounds. The biosensor with the PrecA promotor fixes the presence of the factors causing damage of DNA in a cell [74]. The biosensor *E. coli* MG1655 (pSoxS-lux) was used for the evaluation of prooxidant activity. The biosensor with the PsoxS promoter fixes the production of superoxide anion and NO [75]. Bioluminescent strains were obtained by the transformation of *E. coli* MG1655 by hybrid plasmids pRecA-lux, pSoxS-lux. The gene cassette luxCDABE *Photorhabdus luminescens* under the control PrecA promoters was used in this biosensor. This plasmid was created on the basis of pBR322 and contained a selective marker of ampicillin resistance (Amp gene). The strains were kindly furnished by Manukhov I.V., Federal State Unitary Enterprise “GosNIIGenetika”).

The bacterial strains *Acinetobacter calcoaceticus* VKPM B-10353, *E. coli* MG1655 (pRecA-lux), and *E. coli* MG1655 (pSoxS-lux) were cultivated in Luria–Bertani (LB) medium [76] under constant shaking to early exponential phase at 37 °C. Cells were used immediately for stress induction tests. One hundred micrograms of ampicillin per milliliter were added into LB medium at cultivation of *E. coli* MG1655 (pRecA-lux) and *E. coli* MG1655 (pSoxS-lux). Strain *V. aquamarinus* DSM 26054 was grown in LB medium supplemented with 3% NaCl.

Chemicals. All of the chemicals used were of analytical grade. Crystal violet and *N*-methyl-*N*-nitro-*N*-nitrosoguanidine were obtained from Sigma-Aldrich (USA). Ampicillin was obtained from Sintez (Russia). Azithromycin was obtained from Farmstandart (Russia). Test solutions were prepared in deionized water immediately before the tests. Rat liver microsomal enzymes (S9 fraction) were from Moltox (USA).

The test compounds were dissolved in DMSO to the concentration of 1 × 10^−2^ M. Then, they were diluted with ethanol. The control solutions were analogous dilutions of DMSO in ethanol. The tested compounds were also compared with the standard antibiotic azithromycin. Azithromycin was dissolved in DMSO to the concentration of 5 × 10^−3^ M and then diluted with deionized water.

Biosensors assay procedure. The detailed protocol of toxicity testing by means of a bacterial lux-biosensors is described in the article [77].

Calculation. The criterion of toxic influence was bioluminescence intensity change of the test object in the researched sample in comparison with the control sample.

The induction factor (I) was defined as the relation of luminescence intensity of a lux-biosensor suspension containing tested sample (L_c_) to the luminescence intensity of a lux-biosensor control suspension (L_k_): I = L_c_/L_k_.

If at significant differences from control induction factor values were ≤2, the detected genotoxic effect was evaluated as “weak”, if they were in the range from 2 to 10 as “medium”, and above 10 as “strong”. All the experiments were carried out three times independently.

Difference reliability of bioluminescence in experiment from control value was estimated by *t*-criterion with the help of Excel software. The conclusions about sample toxicity were made at *p* < 0.05.

Test system for evaluation of biofilms production. To quantify the formation of biofilms, the crystal violet assay was used, with some modifications [78]. The necessary concentrations of the test compounds were prepared as described above.

*V. aquamarinus* DSM 26054 was cultivated for 24 h in LB medium supplemented with 3% NaCl in the Innova 40R shaker incubator (New Brunswick Scientific, USA) at 25 °C and 200 rpm. *A. calcoaceticus* VKPM B-10353 was cultivated for 24 h in LB medium in the Innova 40R shaker incubator (New Brunswick Scientific, Enfield, CT, USA) at 30 °C and 200 rpm. Then, the suspensions of the daily culture of *V. aquamarinus* DSM 26054 and *A. calcoaceticus* VKPM B-10353 were diluted with LB medium supplemented with 3% NaCl to the density of 1 × 10^8^ cells/mL.

The resulting suspension (180 μL) was added to the wells of a polystyrene microplate (Nuova Aptaca, Canelli, Italy). To some of the wells, 20 μL of the test substances at various concentrations were added. Since solvents used could also influence the biofilm formation, 20 μL of the appropriate solvent was added to the other part of the wells at same dilutions (control). Six replicates were done for each treatment and control. The microplate was covered with a lid and wrapped with Parafilm (Bemis Company, Inc., Oshkosh, WI, USA).

After incubation at 25 °C for 72 h, biofilms were stained. The contents in the wells were removed by means of a dispenser. The wells were then carefully washed three times with 250 μL of sterile saline. The microplates were shaken to remove all non-adherent bacteria. Biofilms were fixed with 200 μL of 96% ethanol for 15 min. After the microplates had dried in air, 200 μL of 0.5% crystal violet was introduced into the wells. After 10 min, the dye was removed. The excess dye was removed by washing with water three times. After the microplates were air-dried, the dye in the wells bound to biofilms was dissolved with 200 μL of 96% ethanol. The extraction level (absorption) of crystal violet by ethanol was measured after 60 minutes at a wavelength of 570 nm using a FLUOstar Omega microplate reader (BMG Labtech, Offenburg, Germany) in optical density units (OD570). The intensity of biofilm formation directly corresponds to the intensity of staining of the contents of the wells with the dye. Biofilm formation was determined by the difference between the mean OD readings obtained in the presence of compounds and the control.

Each experiment was performed in triplicate. The values were expressed as mean + SD. Student’s *t*-test was used to compare these values. Differences were considered statistically significant at *p* < 0.05.

## 4. Conclusions

In conclusion, a series of novel 2-(het)arylpyrrolidine-1-carboxamides were obtained via a modular approach based on intramolecular cyclization/Mannich-type reaction of *N*-(4,4-diethoxybutyl)ureas. Their anti-cancer activities were tested both in vitro and in vivo. A pyrrolidine derivative possessing a *cyclo*-hexyl substituent in the carboxamide moiety and a benzofuroxan fragment in the pyrrolidine ring was determined as the most active in the in vitro assay. Notably, its activity towards M-Hela tumor cell lines was found to be twice that of reference drug tamoxifen. At the same time, its cytotoxicity towards normal Chang liver cells did not exceed tamoxifen’s toxicity. The obtained results indicate that the death of M-Hela cells presumably occurs via an apoptotic pathway due to activation of the surface cell receptors and not due to mitochondrial dysfunction. In the in vivo studies, water-soluble compounds possessing *N*-(2-(dimethylamino)ethyl)pyrrolidine-1-carboxamide scaffold and either a heterocyclic (hydroxycoumarine) or aromatic (sesamol) substituent in the pyrrolidine core were proven to be the most effective. The number of surviving animals on day 60 of observation ranged from 17% to 83% and increased life span (ILS) ranged from 80% to 447%. Additionally, compounds possessing a benzofuroxan moiety were found to effectively suppress bacterial biofilm growth, and thus are promising candidates for further development as anti-bacterial agents.

## Figures and Tables

**Table 1:** Synthesized library of novel (het)arylpyrrolidines **5**–**8** ^1^.

		(Het)Ar Block			
		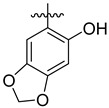	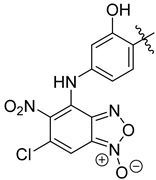	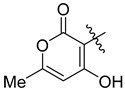	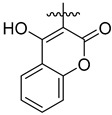
**R (amine block)**	H	**5a** (93)	-	-	-
	Ph	**5b** (98)	**6b** (87)	**7b** (69)	**8b** (51)
	4-MeO-C_6_H_4_	**5c** (91)	**6c** (89)	**7c** (87)	**8c** (45)
	4-Br-C_6_H_4_	**5d** (95)	**6d** (95)	**7d** (75)	**8d** (68)
	4-F-C_6_H_4_	**5e** (91)	**6e** (76)	**7e** (58)	**8e** (51)
	*n*-C_6_H_13_	**5f** (72)	**6f** (44)	**7f** (47)	**8f** (34)
	*cyclo*-C_6_H_11_	**5g** (84)	**6g** (67)	**7g** (60)	**8g** (68)
	TFA•(CH_2_)_2_NMe_2_	**5h** (75)	**6h** (57)	**7h** (77)	**8h** (73)

^1^ Isolated yields are given in parentheses.

**Table 2:** Cytotoxic effects of pyrrolidines **5a**–**6h** on the cancer and normal human cell lines ^1^.

Test Compound	IC_50_ (µM)		Test Compound	IC_50_ (µM)	
	Cancer Cell Line	Normal Cell Line		Cancer Cell Line	Normal Cell Line
	M-Hela	Chang Liver		M-Hela	Chang Liver
**5a**	>100	>100	-	-	-
**5b**	>100	>100	**6b**	56 ± 4.1	>100
**5c**	>100	>100	**6c**	26.0 ± 1.9	62 ± 4.3
**5d**	>100	>100	**6d**	25.5 ± 1.6	53 ± 3.8
**5e**	>100	>100	**6e**	26 ± 1.8	48 ± 3.0
**5f**	>100	>100	**6f**	47 ± 2.9	57 ± 4.3
**5g**	>100	>100	**6g**	14.7 ± 0.9	46 ± 2.7
**5h**	>100	>100	**6h**	100 ± 8.6	>100
			**tamoxifen**	28.0 ± 2.5	46.2 ± 3.5

^1^ Three independent experiments were carried out.

**Table 3:** Mean survival time and increased life span (ILS) of murine leukemia P388 at individual treatment with **5h**, **8h**, **5b**, **6h**, and **7h**.

Compound	Dose									
	18 mg/kg/day		26 mg/kg/day		40 mg/kg/day		55 mg/kg/day		83 mg/kg/day	
	MST ± SD ^1^ (days)	ILS ^2^ (%)	MST ± SD ^1^ (days)	ILS ^2^ (%)	MST ± SD ^1^ (days)	ILS ^2^ (%)	MST ± SD ^1^ (days)	ILS ^2^ (%)	MST ± SD ^1^ (days)	ILS ^2^ (%)
**5b**	27.7 ± 10.3 ***	177	26.5 ± 10.6 ***	165	26.0 ± 10.8 **	160	18.0 ± 8.4 *	80	9.7 ± 0.2	0
**5h**	35.7 ± 10.9 ***	276	18.5 ± 8.3 *	95	52.0 ± 8.0 ***	447	38.0 ± 9.9 ***	300	19.0 ± 8.2 **	100
**6h**	13.8 ± 0.6 ***	37	13.5 ± 0.5 ***	34	12.8 ± 0.7 ***	27	143.8 ± 0.2 ***	39	11.2 ± 1.6	11
**7h**	11.0 ± 0.6 *	0	10.3 ± 0.2	0	10.2 ± 0.2	0	10.7 ± 0.2	0	10.3 ± 0.2	0
**8h**	19.3 ± 8.1 *	93	20.7 ± 8.0 **	107	13.2 ± 1.6 ***	32	19.8 ± 8.1 **	98	26.2 ± 10.7 **	162

^1^ MST: mean survival time; SD: standard deviation; ^2^ ILS%: the percentage of the median survival time (MST) of the treated group (t) to that of the control group (c). ILS% = (MSTt/MSTc) × 100. * Statistically significant increase over the control (*p* < 0.05); ** Statistically significant increase over the control (*p* < 0.01); *** Statistically significant increase over the control (*p* < 0.001).

**Table 4:** Effect of compounds **5b**, **5h**, **6h**, **7h**, and **8h** against i.p. implanted P388 murine tumors.

Compound	Dose									
	18 mg/kg/day		26 mg/kg/day		40 mg/kg/day		55 mg/kg/day		83 mg/kg/day	
	Survivors/Total ^1^	Survival, %	Survivors/Total ^1^	Survival, %	Survivors/Total ^1^	Survival, %	Survivors/Total ^1^	Survival, %	Survivors/Total ^1^	Survival, %
**Control**	0/6	0	0/6	0	0/6	0	0/6	0	0/6	0
**5b**	2/6	33	2/6	33	2/6	33	2/6	17	0/6	0
**5h**	3/6	50	1/6	17	5/6	83	3/6	50	1/6	17
**6h**	0/6	0	0/6	0	0/6	0	0/6	0	0/6	0
**7h**	0/6	0	0/6	0	0/6	0	0/6	0	0/6	0
**8h**	1/6	17	1/6	17	1/6	0	1/6	17	1/6	33

^1^ Survivors on day 60.

**Table 5:** Biofilm formation (%) by *Vibrio aquamarinus* DSM 26054 and *Acinetobacter calcoaceticus* VKPM B-10353 in the presence of pyrrolidine-1-carboxamides possessing benzofuroxan moiety in reference to control (control = 100%).

№	Compound	Strain	Biofilm Formation, %				
			Compound Concentration, M				
			1 × 10^−9^	1 × 10^−8^	1 × 10^−7^	1 × 10^−6^	1 × 10^−5^
**1**	**6a**	*V. aquamarinus* DSM 26054	17.70 *	10.23 *	20.34 *	16.49 *	42.65 *
		*A. calcoaceticus* VKPM B-10353	79.58 *	42.37 *	54.83 *	95.00	86.19
**2**	**6b**	*V. aquamarinus* DSM 26054	8.21 *	15.42 *	26.04 *	39.02 *	89.12
		*A. calcoaceticus* VKPM B-10353	90.41	70.79 *	73.93 *	88.44	119.69 *
**3**	**6c**	*V. aquamarinus* DSM 26054	13.54 *	14.67 *	9.22 *	18.28 *	26.65 *
		*A. calcoaceticus* VKPM B-10353	93.75	55.74 *	72.77 *	79.31 *	82.82
**4**	**6d**	*V. aquamarinus* DSM 26054	67.29 *	6.46 *	10.70 *	24.11 *	63.12
		*A. calcoaceticus* VKPM B-10353	79.16 *	46.95 *	50.00 *	95.71	89.50
**5**	**6e**	*V. aquamarinus* DSM 26054	67.29	17.96 *	11.01 *	26.02 *	39.57 *
		*A. calcoaceticus* VKPM B-10353	87.92 *	47.66 *	55.40 *	53.27 *	86.50
**6**	**4**	*V. aquamarinus* DSM 26054	66.58	9.31 *	11.34 *	43.56 *	96.57
		*A. calcoaceticus* VKPM B-10353	86.67 *	62.12 *	78.63 *	97.99	149.22*
**7**	**Azithromycin**	*V. aquamarinus* DSM 26054	103.42	102.43	99.39	103.80	81.50 *
		*A. calcoaceticus* VKPM B-10353	95.00	96.07	105.71	106.07	101.79

* Differences compared to the control samples are statistically significant, *t* criterion, *p* < 0,05; the solutions of appropriate solvent in ethanol with the same concentration were used as control in experiments with pyrrolidine-1-carboxamides; six replicates were done for each treatment and control.

**Table 6:** Genotoxicity (induction factor, I) of the pyrrolidine-1-carboxamides possessing a benzofuroxan moiety registered with the bacterial lux-biosensor *Escherichia coli* MG1655 (pRecA-lux).

Compound	Activation ^1^	Concentration of Compound, M				
		10^−9^	10^−8^	10^−7^	10^−6^	10^−5^
**6a**	-	0.65 ± 0.01	0.88 ± 0.06	0.88 ± 0.03	0.61 ± 0.03	0.54 ± 0.01
	+	0.72 ± 0.04	0.86 ± 0.01	0.80 ± 0.03	0.70 ± 0.05	0.65 ± 0.06
**6b**	-	2.34 ± 0.06 *	2.28 ± 0.12 *	2.07 ± 0.11 *	2.15 ± 0.06 *	1.77 ± 0.04 *
	+	1.47 ± 0.04	1.31 ± 0.01	1.30 ± 0.02	1.36 ± 0.04	1.23 ± 0.02
**6c**	-	2.67 ± 0.07 *	2.84 ± 0.02 *	2.64 ± 0.14 *	2.82 ± 0.05 *	2.33 ± 0.04 *
	+	1.30 ± 0.01	1.37 ± 0.02	1.49 ± 0.02 *	1.54 ± 0.03 *	1.35 ± 0.02
**6d**	-	0.64 ± 0.03	0.70 ± 0.01	0.62 ± 0.03	0.70 ± 0.01	0.60 ± 0.02
	+	0.76 ± 0.05	0.79 ± 0.03	0.71 ± 0.05	0.71 ± 0.03	0.66 ± 0.04
**6e**	-	2.56 ± 0.10 *	2.74 ± 0.04 *	2.46 ± 0.02 *	2.39 ± 0.02 *	2.00 ± 0.02 *
	+	1.43 ± 0.01	1.35 ± 0.04	1.45 ± 0.02	1.47 ± 0.04	1.24 ± 0.02
**4**	-	1.21 ± 0.04	1.27 ± 0.07	1.14 ± 0.02	1.27 ± 0.01	1.10 ± 0.00
	+	1.19 ± 0.01	1.11 ± 0.02	1.18 ± 0.01	1.28 ± 0.04	1.11 ± 0.01

^1^ Variants with metabolic activation (+S9) and without it (-S9); * difference from the control experiment are statistically significant, *t*-test; *p* < 0.05

**Table 7:** Prooxidant activity (induction factor) of the pyrrolidine-1-carboxamides possessing a benzofuroxan moiety registered with the bacterial lux-biosensor *E. coli* MG1655 (pSoxS-lux).

Compound	Concentration of Compound, M				
	10^−9^	10^−8^	10^−7^	10^−6^	10^−5^
**6a**	1.54 ± 0.13 *	1.70 ± 0.23 *	1.36 ± 0.03	1.46 ± 0.06	1.24 ± 0.08
**6b**	1.03 ± 0.10	1.21 ± 0.00	1.04 ± 0.04	1.24 ± 0.11	0.89 ± 0.03
**6c**	1.11 ± 0.03	1.54 ± 0.20 *	1.68 ± 0.20 *	1.46 ± 0.03	0.96 ± 0.09
**6d**	1.34 ± 0.09	1.11 ± 0.07	1.11 ± 0.01	1.60 ± 0.23 *	1.10 ± 0.07
**6e**	0.93 ± 0.03	1.25 ± 0.28	1.85 ± 0.15 *	1.34 ± 0.10	0.84 ± 0.04
**4**	1.85 ± 0.10 *	2.08 ± 0.05 *	1.74 ± 0.14 *	1.54 ± 0.07 *	1.64 ± 0.04 *

* Difference from the control experiment are statistically significant, *t*-test; *p* < 0.05.

## Supplementary Materials

The following are available online at https://www.mdpi.com/1420-3049/24/17/3086/s1, Figures S1–S14 (Anti-biofilm activity data), Figure S15, Tables S1–S4 (X-ray data); Figure S16 (In vivo anti-cancer activity data), copies of NMR spectra of all synthesized compounds.
