# Supplementary material for: Synthesis of Novel 2-(Het)arylpyrrolidine Derivatives and Evaluation of Their Anticancer and Anti-Biofilm Activity
Source: Molecules. 2019 Aug 25;24(17):3086. doi: 10.3390/molecules24173086 (PMC6749236; doi:10.3390/molecules24173086)
Supplement: Supplementary file 1 [file molecules-24-03086-s001.pdf]

SUPPLEMENTARY MATERIALS

SYNTHESIS OF NOVEL 2-(HET)ARYLPYRROLIDINE DERIVATIVES AND EVALUATION OF THEIR ANTICANCER AND ANTI-BIOFILM ACTIVITY

Andrey Smolobochkin,[1] Almir Gazizov,[1]* Marina Sazykina,[2] Nurgali Akylbekov,[3] Elena Chugunova,[1,4]* Ivan Sazykin,[2] Anastasiya Gildebrant,[2] Julia Voronina,[5] Alexander Burilov,[1] Shorena Karchava,[2] Maria Klimova,[2] Alexandra Voloshina,[1] Anastasia Sapunova,[1] Elena Klimanova,[6] Tatyana Sashenkova,[6] Ugulzhan Allayarova,[6] Anastasiya Balakina,[6,7] Denis Mishchenko[6,7]

Arbuzov Institute of Organic and Physical Chemistry, FRC Kazan Scientific Center of RAS, Russia, 420088, Kazan, Arbuzov str., 8

[2] *Southern Federal University, Russia, 344090, Rostov-on-Don, Stachki Avenue, 194/2*

[3] *Institute of Chemical Research and Technology of Korkyt Ata Kyzylorda State University, The Republic of Kazakhstan, 120014, Kyzylorda, Aiteke bie str., 29A*

[4] *Kazan Federal University, Russia, 420008, Kazan, Kremlyovskaya str., 18*

[5] *N. S. Kurnakov Institute of General and Inorganic Chemistry, RAS, 31 Leninsky Av., 119991 Moscow, Russian Federation*

[6] *Institute of Problems of Chemical Physics RAS, Chernogolovka 142432, Russia*

[7] *Scientific and Educational Center in Chernogolovka of Moscow Region State University, Mytishi, 141014, Russia*

* *Correspondence: chugunova.e.a@gmail.com, Tel.: +7 843 272 7324 (Elena Chugunova); agazizov@iopc.ru; Tel.: +7 843 272 7324 (Almir Gazizov)*

Contents

Anti-biofilm activity studies 2

X-ray studies 9

In vivo anti-cancer activity 11

In vitro anti-cancer activity 11

Copies of NMR spectra 12

Anti-biofilm activity studies

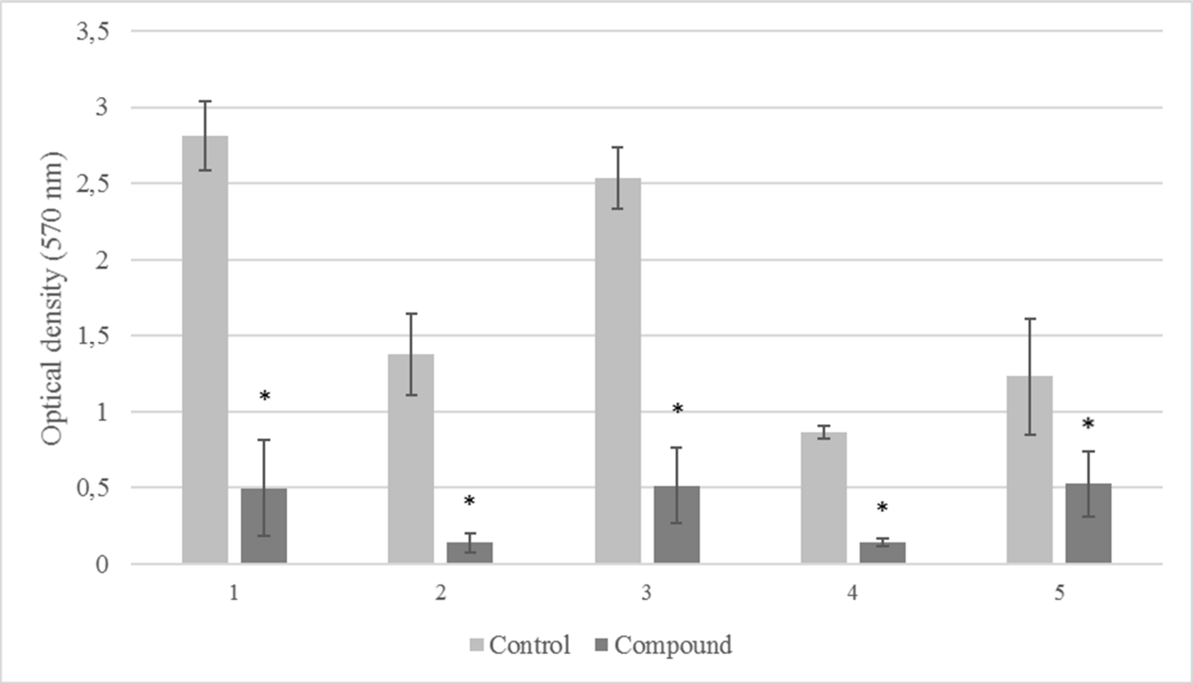

Figure S 1. Antibiofilm activity of pyrrolidine **6a** against *V. aquamarinus* DSM 26054: 1 – **6a** (1×10^{-9} M); 2 – **6a** (1×10^{-8} M); 3 – **6a** (1×10^{-7} M); 4 – **6a** (1×10^{-6} M); 5 – **6a** (1×10^{-5} M). The solutions of appropriate solvent in ethanol with the same concentration were used as controls. Each experiment was performed in triplicate and repeated in six different occasions. The values were expressed as mean ± SD. Student's T-test was used to compare these values. *Differences were considered statistically significant at $p < 0.05$.

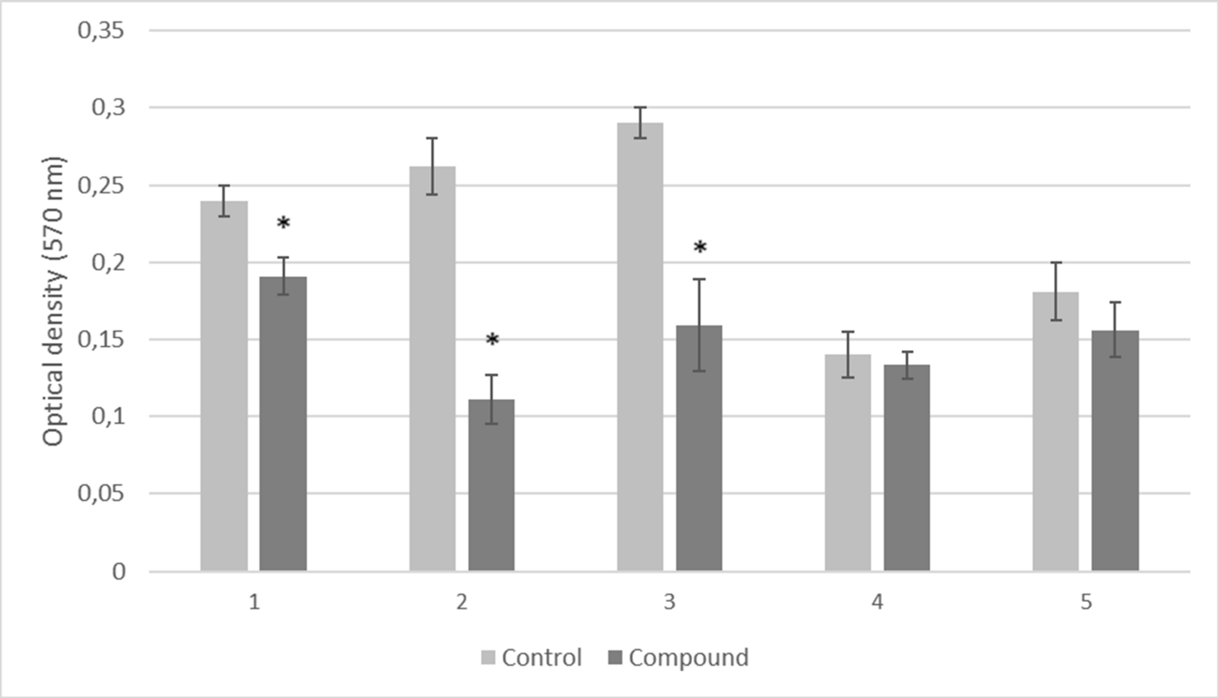

Figure S 2. Antibiofilm activity of pyrrolidine **6a** against *A. calcoaceticus* VKPM B-10353: 1 – **6a** (1×10^{-9} M); 2 – **6a** (1×10^{-8} M); 3 – **6a** (1×10^{-7} M); 4 – **6a** (1×10^{-6} M); 5 – **6a** (1×10^{-5} M). The solutions of appropriate solvent in ethanol with the same concentration were used as controls. Each experiment was performed in triplicate and repeated in six different occasions. The values were expressed as mean ± SD. Student's T-test was used to compare these values. *Differences were considered statistically significant at $p < 0.05$.

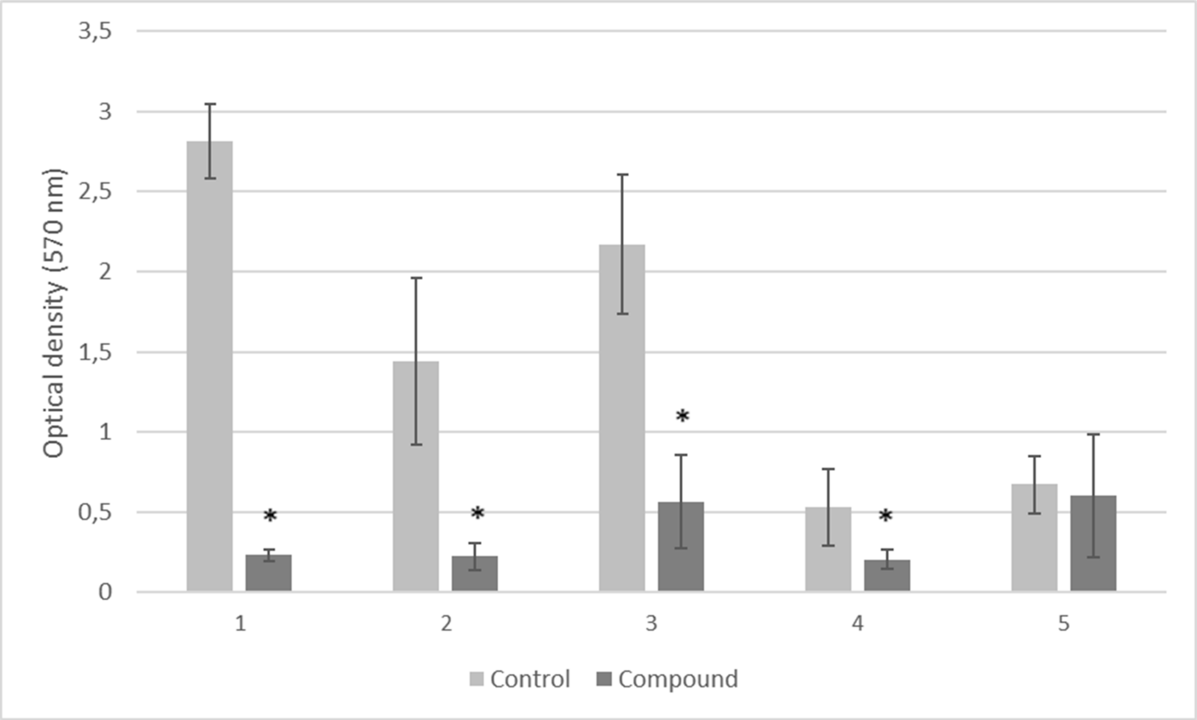

Figure S 3. Antibiofilm activity of pyrrolidine **6b** against *V. aquamarinus* DSM 26054: 1 – **6b** (1×10^{-9} M); 2 – **6b** (1×10^{-8} M); 3 – **6b** (1×10^{-7} M); 4 – **6b** (1×10^{-6} M); 5 – **6b** (1×10^{-5} M). The solutions of appropriate solvent in ethanol with the same concentration were used as controls. Each experiment was performed in triplicate and repeated in six different occasions. The values were expressed as mean ± SD. Student's T-test was used to compare these values. *Differences were considered statistically significant at $p < 0.05$.

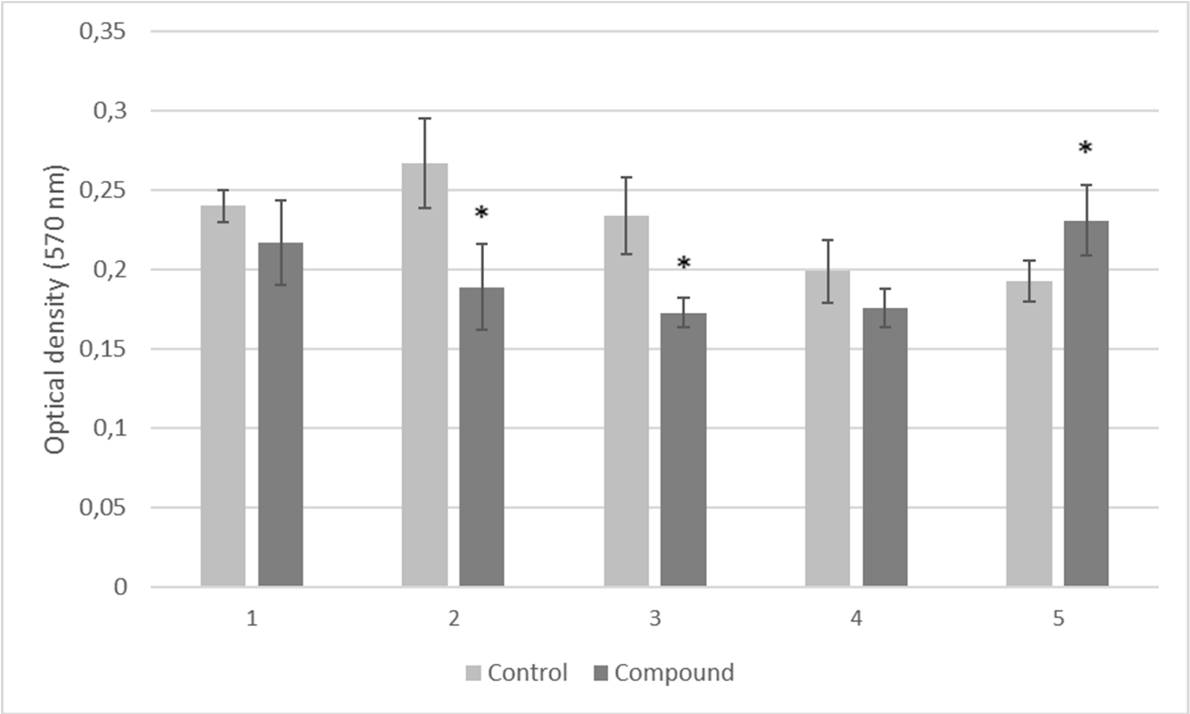

Figure S 4. Antibiofilm activity of pyrrolidine **6b** against *A. calcoaceticus* VKPM B-10353: 1 – **6b** (1×10^{-9} M); 2 – **6b** (1×10^{-8} M); 3 – **6b** (1×10^{-7} M); 4 – **6b** (1×10^{-6} M); 5 – **6b** (1×10^{-5} M). The solutions of appropriate solvent in ethanol with the same concentration were used as controls. Each experiment was performed in triplicate and repeated in six different occasions. The values were expressed as mean ± SD. Student's T-test was used to compare these values. *Differences were considered statistically significant at $p < 0.05$.

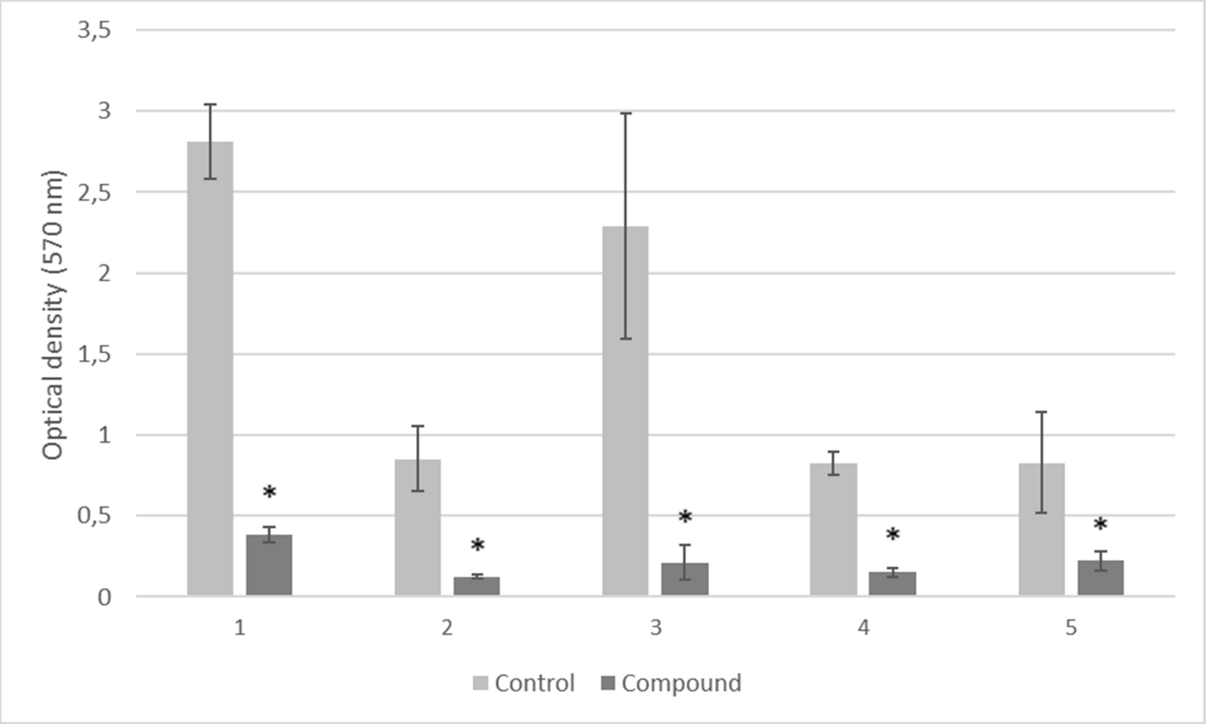

Figure S 5. Antibiofilm activity of pyrrolidine **6c** against V. aquamarinus DSM 26054: 1 – **6c** (1×10^{-9} M); 2 – **6c** (1×10^{-8} M); 3 – **6c** (1×10^{-7} M); 4 – **6c** (1×10^{-6} M); 5 – **6c** (1×10^{-5} M). The solutions of appropriate solvent in ethanol with the same concentration were used as controls. Each experiment was performed in triplicate and repeated in six different occasions. The values were expressed as mean $\pm$ SD. Student's T-test was used to compare these values. *Differences were considered statistically significant at $p < 0.05$.

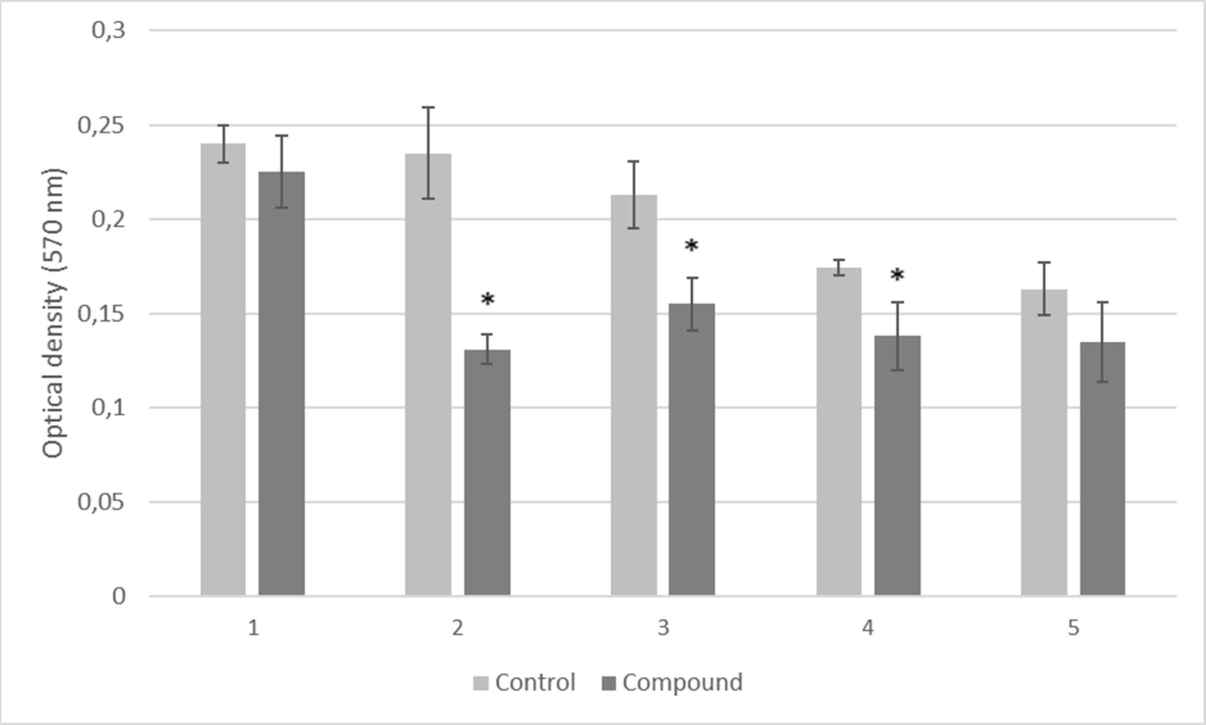

Figure S 6. Antibiofilm activity of pyrrolidine **6c** against *A. calcoaceticus* VKPM B-10353: 1 – **6c** (1×10^{-9} M); 2 – **6c** (1×10^{-8} M); 3 – **6c** (1×10^{-7} M); 4 – **6c** (1×10^{-6} M); 5 – **6c** (1×10^{-5} M). The solutions of appropriate solvent in ethanol with the same concentration were used as controls. Each experiment was performed in triplicate and repeated in six different occasions. The values were expressed as mean $\pm$ SD. Student's T-test was used to compare these values. *Differences were considered statistically significant at $p < 0.05$.

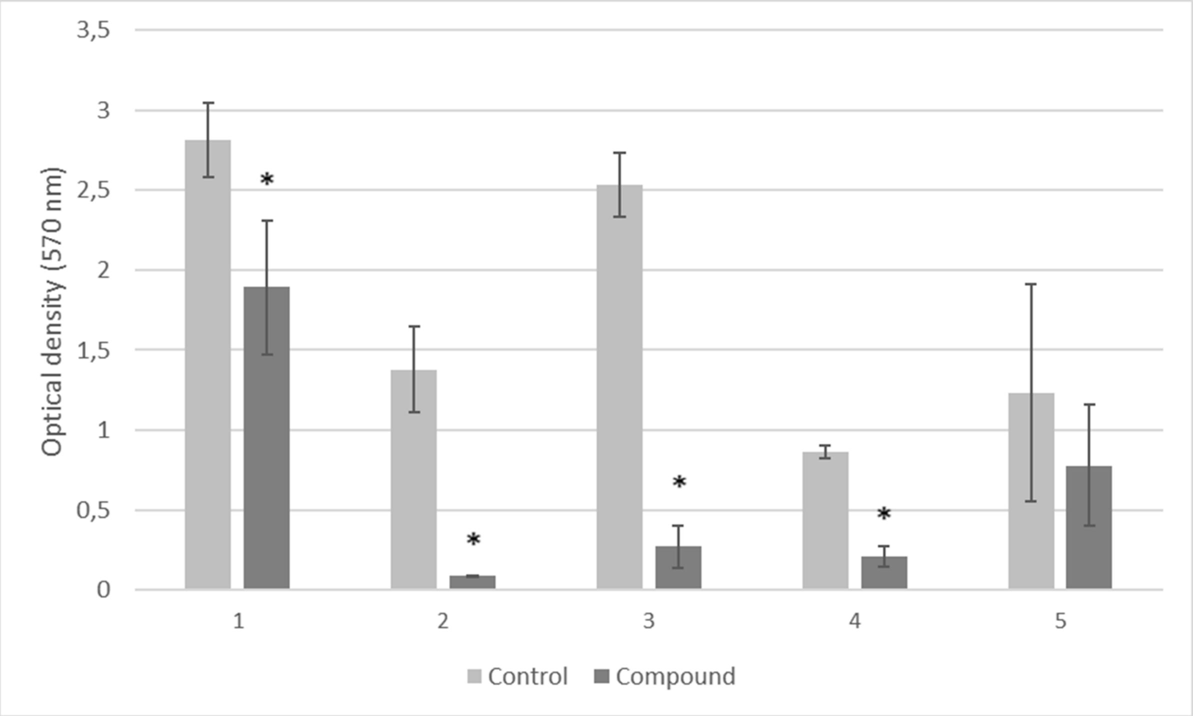

Figure S 7. Antibiofilm activity of pyrrolidine **6d** against *V. aquamarinus* DSM 26054: 1 – **6d** (1×10^{-9} M); 2 – **6d** (1×10^{-8} M); 3 – **6d** (1×10^{-7} M); 4 – **6d** (1×10^{-6} M); 5 – **6d** (1×10^{-5} M). The solutions of appropriate solvent in ethanol with the same concentration were used as controls. Each experiment was performed in triplicate and repeated in six different occasions. The values were expressed as mean ± SD. Student's T-test was used to compare these values. *Differences were considered statistically significant at $p < 0.05$.

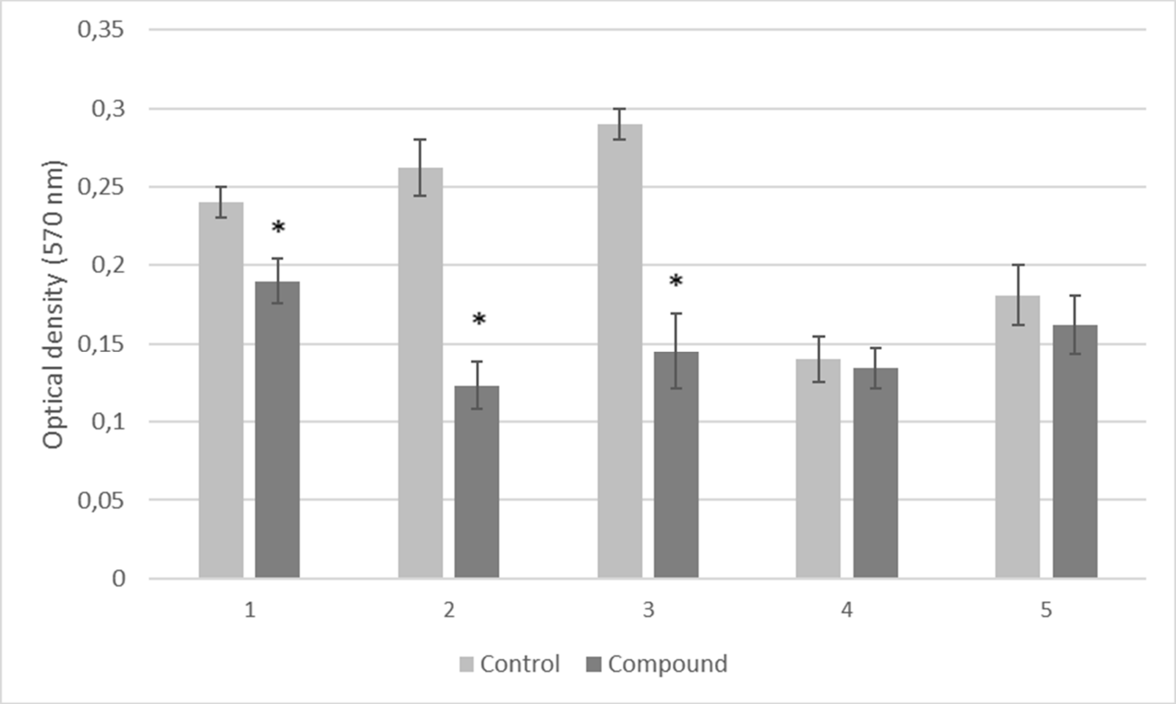

Figure S 8. Figure 4 – Antibiofilm activity of pyrrolidine **6d** against *A. calcoaceticus* VKPM B-10353: 1 – **6d** (1×10^{-9} M); 2 – **6d** (1×10^{-8} M); 3 – **6d** (1×10^{-7} M); 4 – **6d** (1×10^{-6} M); 5 – **6d** (1×10^{-5} M). The solutions of appropriate solvent in ethanol with the same concentration were used as controls. Each experiment was performed in triplicate and repeated in six different occasions. The values were expressed as mean ± SD. Student's T-test was used to compare these values. *Differences were considered statistically significant at $p < 0.05$.

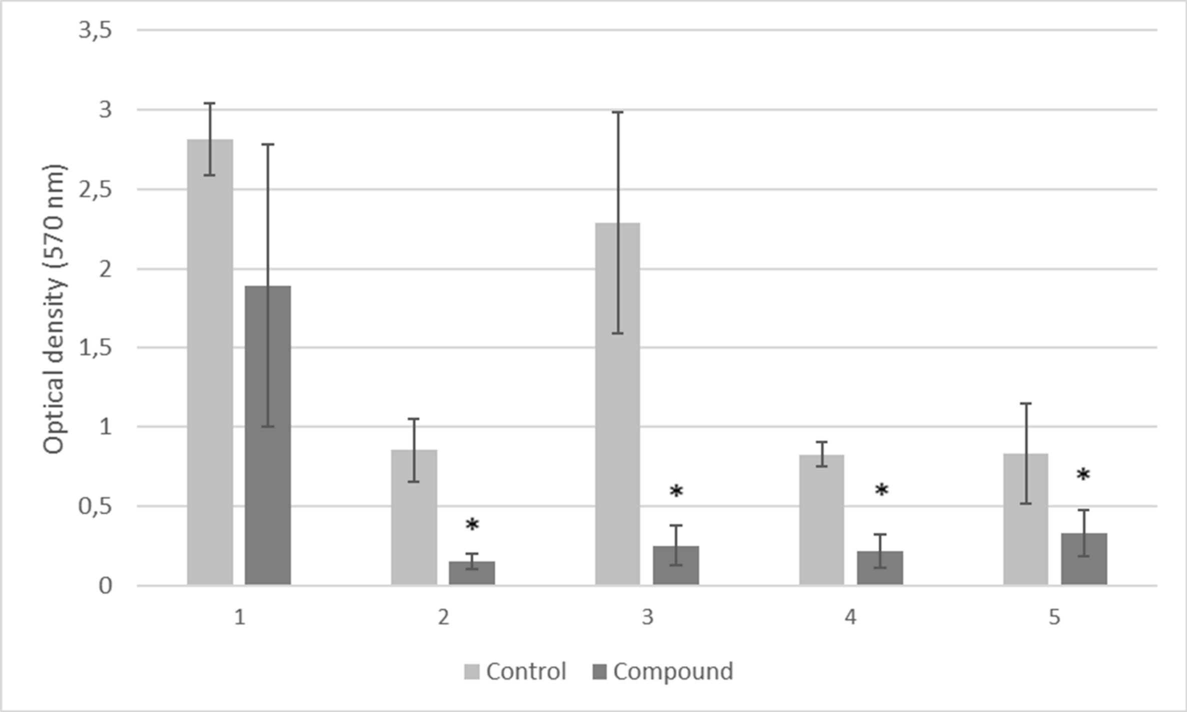

Figure S 9. Antibiofilm activity of pyrrolidine **6e** against *V. aquamarinus* DSM 26054: 1 – **6e** (1×10^{-9} M); 2 – **6e** (1×10^{-8} M); 3 – **6e** (1×10^{-7} M); 4 – **6e** (1×10^{-6} M); 5 – **6e** (1×10^{-5} M). The solutions of appropriate solvent in ethanol with the same concentration were used as controls. Each experiment was performed in triplicate and repeated in six different occasions. The values were expressed as mean $\pm$ SD. Student's T-test was used to compare these values. *Differences were considered statistically significant at $p < 0.05$.

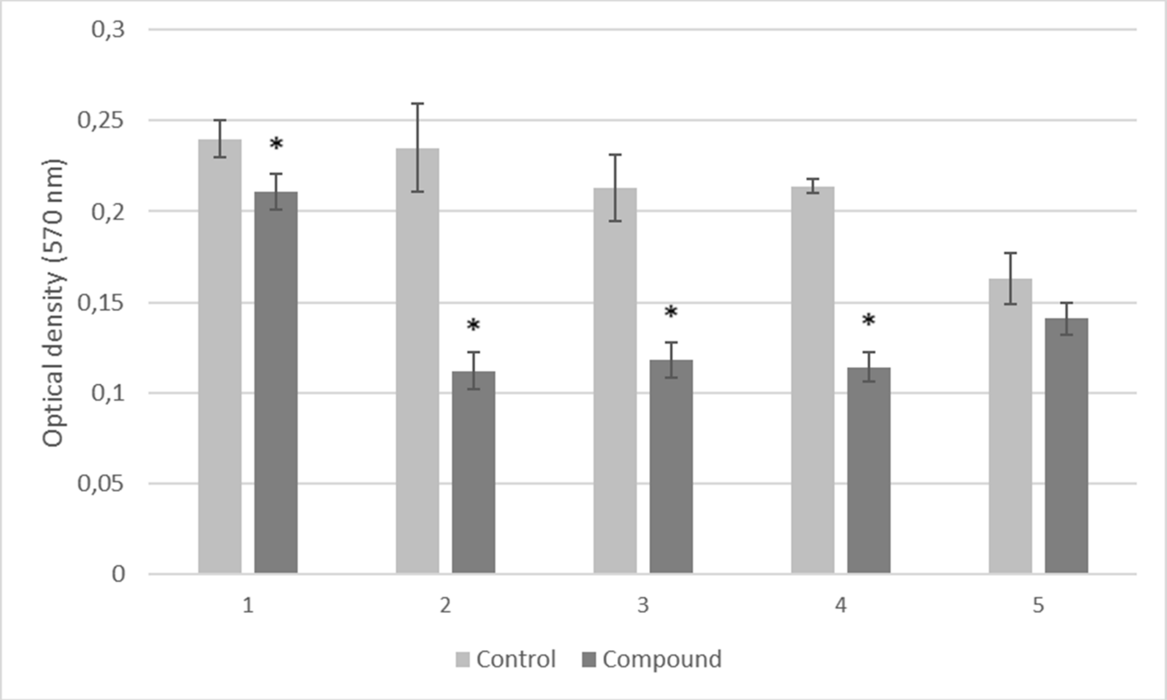

Figure S 10. Antibiofilm activity of pyrrolidine **6e** against *A. calcoaceticus* VKPM B-10353: 1 – **6e** (1×10^{-9} M); 2 – **6e** (1×10^{-8} M); 3 – **6e** (1×10^{-7} M); 4 – **6e** (1×10^{-6} M); 5 – **6e** (1×10^{-5} M). The solutions of appropriate solvent in ethanol with the same concentration were used as controls. Each experiment was performed in triplicate and repeated in six different occasions. The values were expressed as mean $\pm$ SD. Student's T-test was used to compare these values. *Differences were considered statistically significant at $p < 0.05$.

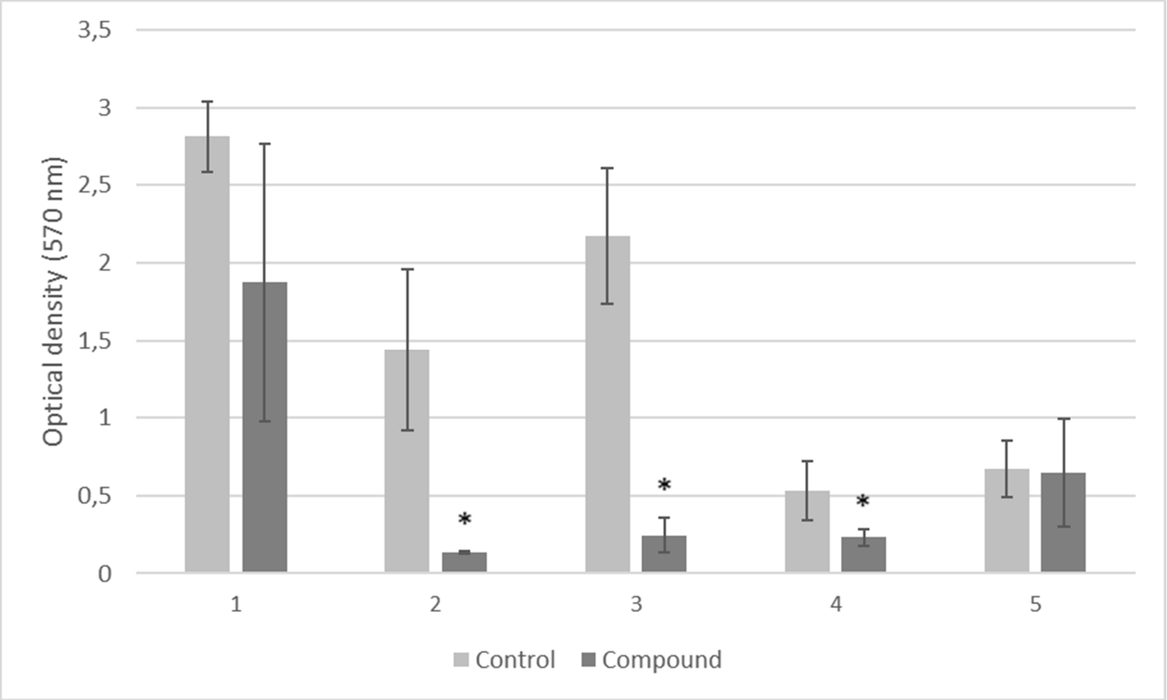

Figure S 11. Antibiofilm activity of phenol **4** against *V. aquamarinus* DSM 26054: 1 – **4** (1×10^{-9} M); 2 –**4** (1×10^{-8} M); 3 – **4** (1×10^{-7} M); 4 – **4** (1×10^{-6} M); 5 – **4** (1×10^{-5} M). The solutions of appropriate solvent in ethanol with the same concentration were used as controls. Each experiment was performed in triplicate and repeated in six different occasions. The values were expressed as mean + SD. Student's T-test was used to compare these values. *Differences were considered statistically significant at p < 0.05.

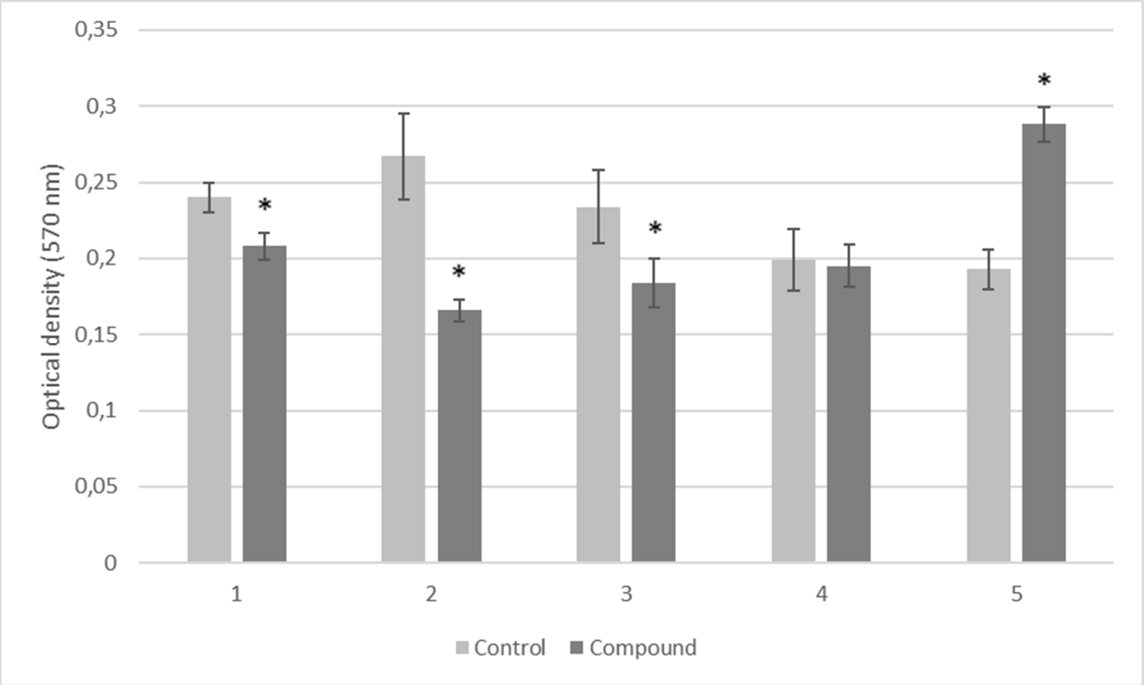

Figure S 12. Figure 12 – Antibiofilm activity of phenol **4** against *A. calcoaceticus* VKPM B-10353: 1 – **4** (1×10^{-9} M); 2 – **4** (1×10^{-8} M); 3 – **4** (1×10^{-7} M); 4 – **4** (1×10^{-6} M); 5 – **4** (1×10^{-5} M). The solutions of appropriate solvent in ethanol with the same concentration were used as controls. Each experiment was performed in triplicate and repeated in six different occasions. The values were expressed as mean $\pm$ SD. Student's T-test was used to compare these values. *Differences were considered statistically significant at *p* < 0.05.

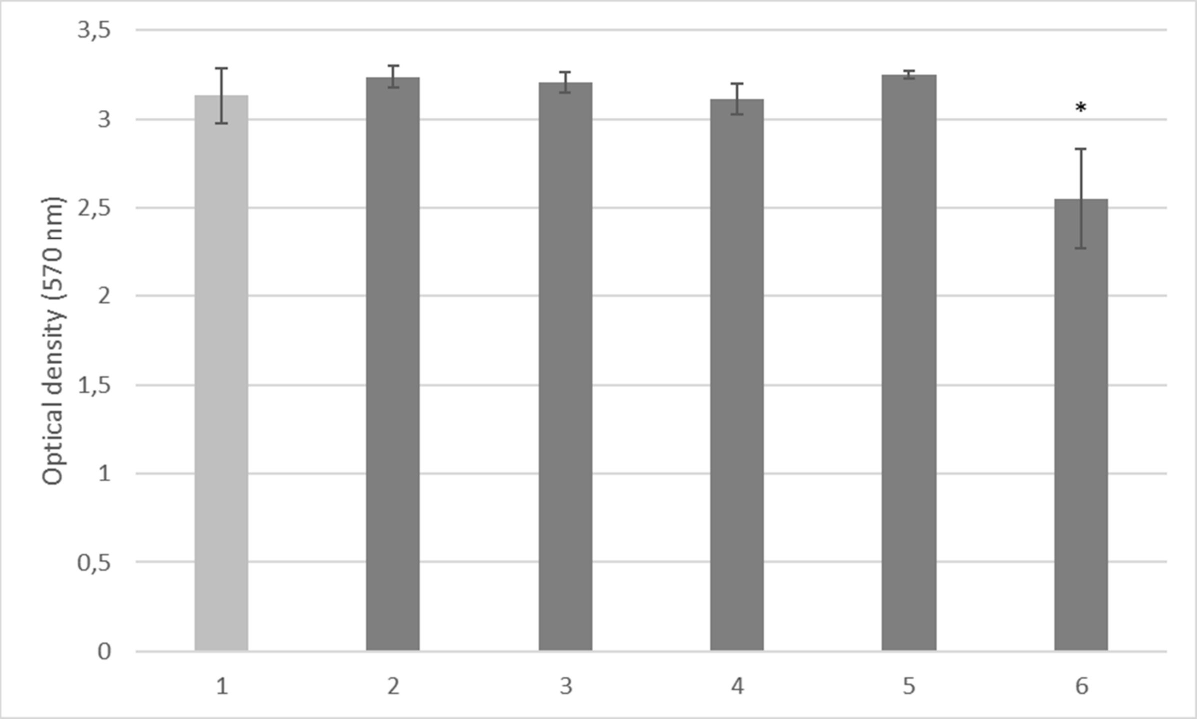

Figure S 13. Antibiofilm activity of azithromycin against *V. aquamarinus* DSM 26054: 1 – control; 2 – azithromycin (1×10^{-9} M); 3 – azithromycin (1×10^{-8} M); 4 – azithromycin (1×10^{-7} M); 5 – azithromycin (1×10^{-6} M); 6 – azithromycin (1×10^{-5} M). Deionized H_2O was used as control. Each experiment was performed in triplicate and repeated in six different occasions. The values were expressed as mean + SD. Student's T-test was used to compare these values. *Differences were considered statistically significant at p < 0.05.

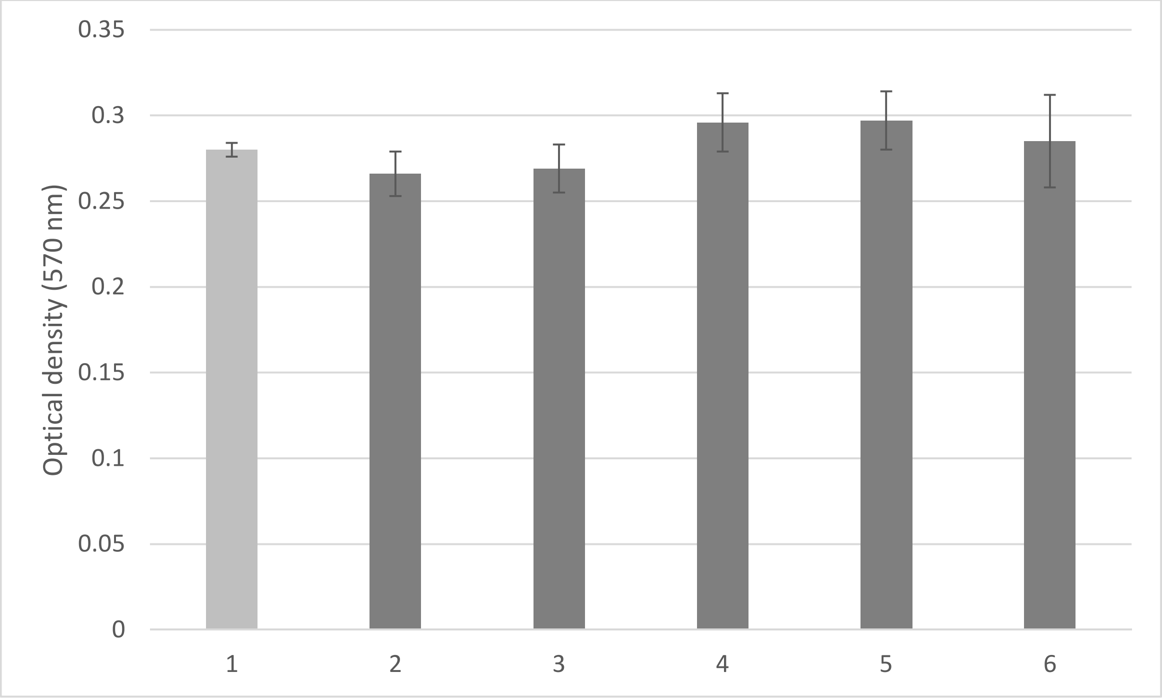

Figure S 14. Antibiofilm activity of azithromycin against *A. calcoaceticus* VKPM B-10353: 1 – control; 2 – azithromycin (1×10^{-9} M); 3 – azithromycin (1×10^{-8} M); 4 – azithromycin (1×10^{-7} M); 5 – azithromycin (1×10^{-6} M); 6 – azithromycin (1×10^{-5} M). Deionized H_2O was used as control. Each experiment was performed in triplicate and repeated in six different occasions. The values were expressed as mean $\pm$ SD. Student's T-test was used to compare these values. *Differences were considered statistically significant at $p < 0.05$.

X-ray studies

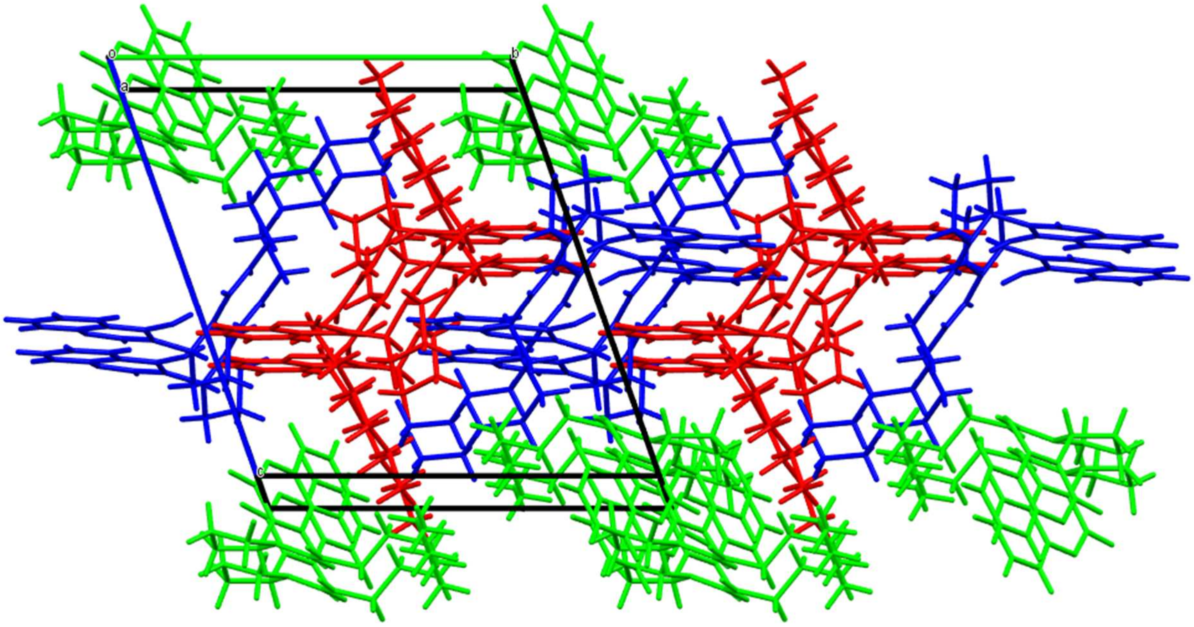

Figure S 15. Crystal packing of compound **8f** (molecules of different symmetry equivalents are shown in different colors).

Table S 1. Torsion angles in pyrrolidine and hexane substituents in compound **8f**.

Torsion angle	a	b	c	Torsion angle	a	b	c
N1-C2-C3-C4	18.0(3)	21.0(3)	36.1(3)	C16-N17-C18-C19	83.4(3)	98.6(3)	90.2(3)
C2-C3-C4-C5	30.0(3)	33.6(3)	35.3(3)	N17-C18-C19-C20	173.5(2)	171.8(3)	54.8(3)
C3-C4-C5-N1	29.7(3)	32.6(3)	20.6(3)	C18-C19-C20-C21	66.5(4)	49.3(5)	170.3(2)
C5-N1-C2-C3	0.8(3)	0.6(3)	23.9(3)	C19-C20-C21-C22	61.3(4)	179.0(3)	173.3(2)
C2-N1-C5-C4	19.0(3)	19.9(3)	2.1(3)	C20-C21-C22-C23	172.4(3)	175.8(4)	174.4(3)

Table S 2. H-bonds in crystal of compound **8f**.

H-bond	D-H	H...A	D...A	D-H...A
O15B-H15B...O16B	0.86	1.68	2.536(3)	173
O15C-H15C...O16C	0.85	1.72	2.550(3)	165
O15A-H16A...O16A	0.85	1.66	2.508(3)	171
N17A-H17A...O7A	0.88	2.10	2.921(3)	156
N17B-H17B...O7B	1.02	1.91	2.842(3)	151
N17C-H17C...O7C	0.89	1.98	2.867(3)	171
C2A-H2AA...O15A	1.00	2.48	2.882(3)	103
C3A-H3AB...O7A	0.99	2.44	2.914(3)	109
C4A-H4AB...O7A	0.99	2.54	3.131(3)	118
C5A-H5AB...O7A	0.99	2.52	3.338(3)	140

H-bond	D-H	H...A	D...A	D-H...A
C2B-H2BA...O15B	1.00	2.49	2.893(3)	104
C3B-H3BB...O7B	0.99	2.31	3.003(4)	126
C12B-H12B...O16C	0.95	2.38	3.251(3)	152
C18B-H18D...O16B	0.99	2.42	2.765(3)	100
C4C-H4CA...O7C	0.99	2.32	3.006(3)	126
C5C-H5CA...O15C	1.00	2.48	2.890(3)	104

Table S 3. π…π interactions in crystal of compound **8f**.

Π…π	Cg-Cg	Alpha	CgI_Perp	CgJ_Perp
Cg2...Cg3	3.4890(16)	0.88(13)	3.4838(11)	3.4858(11)
Cg3...Cg2	3.4890(16)	0.88(13)	3.4858(11)	3.4838(11)
Cg7...Cg11	3.7432(16)	1.41(13)	-3.4105(11)	-3.3934(11)
Cg11...Cg7	3.7432(16)	1.41(13)	-3.3934(11)	-3.4105(11)

Table S 4. CH…π interactions in crystal of compound **8f**.

C-H…π	H..Cg	H-Perp	Gamma	X-H..Cg
C2A-H2AA...Cg11	2.89	2.86	7.89	143
C21A-H21B...Cg6	2.78	2.73	10.66	144
C22C-H22E...Cg3	2.66	-2.65	6.12	168

In vivo anti-cancer activity

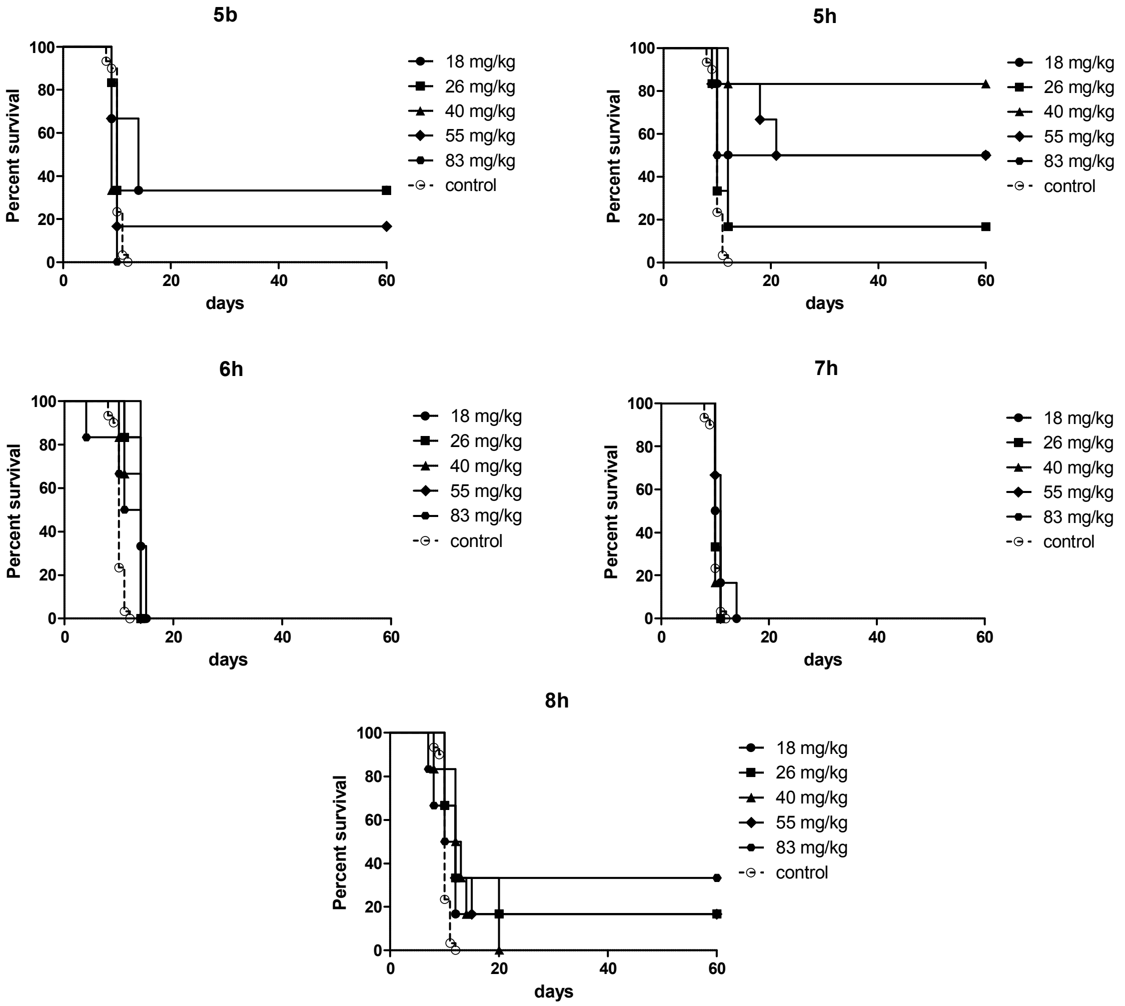

Figure S 16. The Kaplan-Meier curves demonstrating the percentage of survival of mice bearing P388 leukemia as a function of time.

In vitro anti-cancer activity

Table S 5. Cytotoxic effects of pyrrolidines **7b–8h** on the cancer and normal human cell lines.[1]

Test compounds	IC_{50} (μM)		Test compounds	IC_{50} (μM)	
	Cancer cell line	Normal cell line		Cancer cell line	Normal cell line
	M-Hela	Chang liver		M-Hela	Chang liver
**7b**	>100	>100	**8b**	>100	>100
**7c**	>100	>100	**8c**	>100	>100
**7d**	>100	>100	**8d**	>100	>100
**7e**	>100	>100	**8e**	>100	>100
**7f**	>100	>100	**8f**	>100	>100
**7g**	>100	>100	**8g**	>100	>100
**7h**	>100	>100	**8h**	>100	>100

[1] Three independent experiments were carried out

Copies of NMR spectra

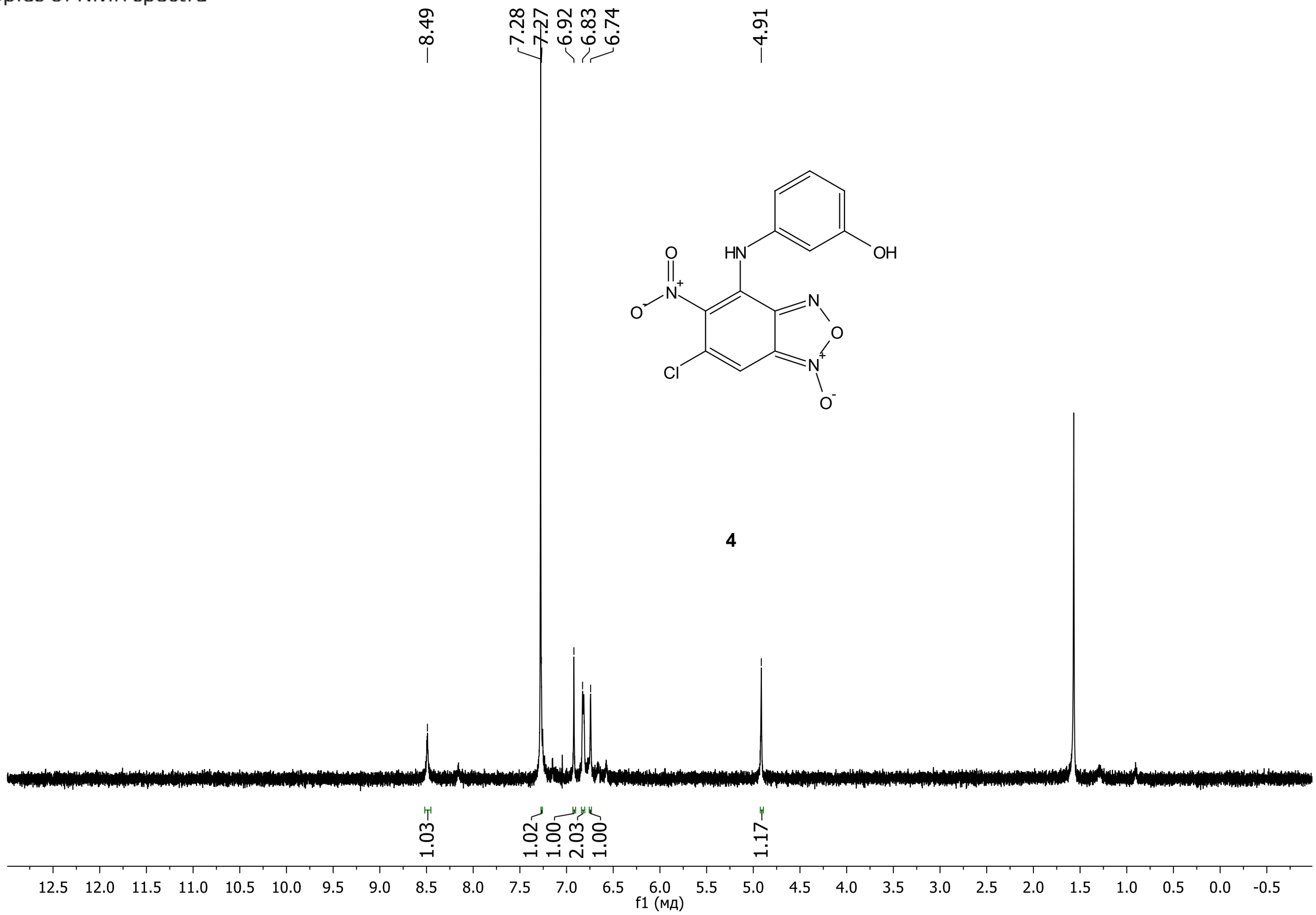

Figure S 17.

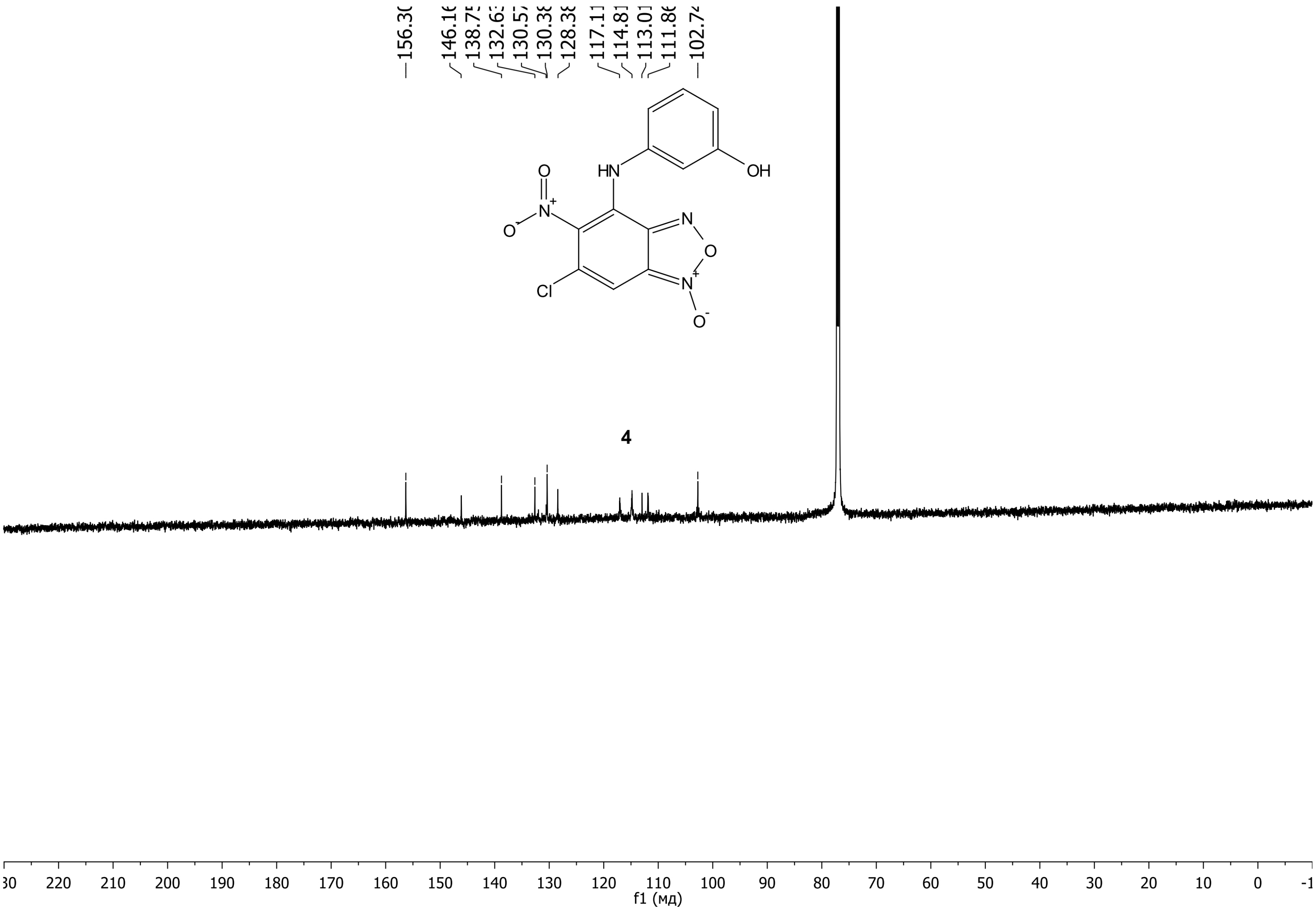
156.3
146.1
138.7
132.6
130.5
130.3
128.3
117.1
114.8
113.0
111.8
102.7
HN
OH
O
N+
O-
N
O
N+
O-
Cl
4
30 220 210 200 190 180 170 160 150 140 130 120 110 100 90 80 70 60 50 40 30 20 10 0 -1
f1 (мд)

Figure S 18.

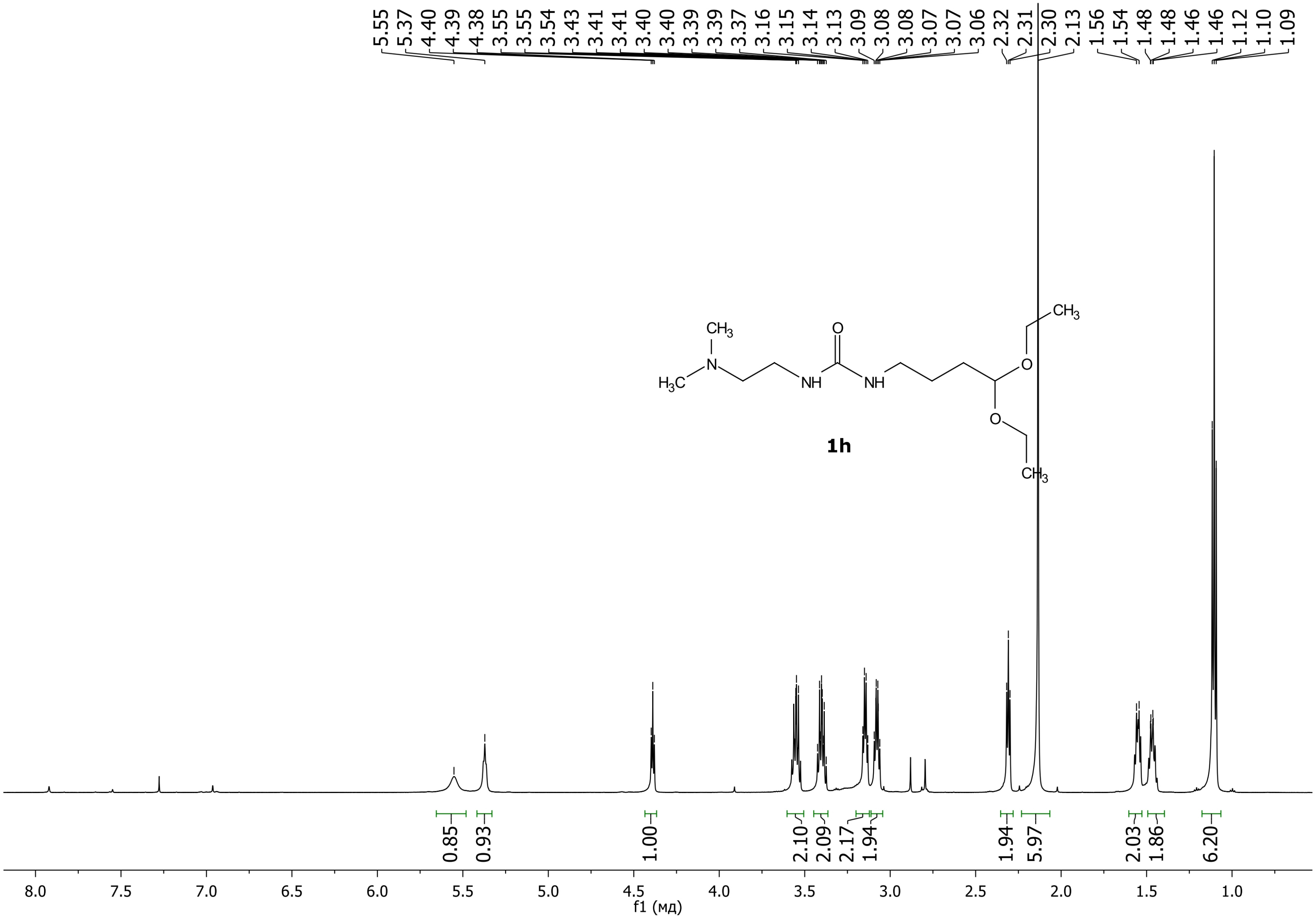
5.55
5.37
4.40
4.39
4.38
3.55
3.55
3.54
3.43
3.41
3.41
3.40
3.40
3.39
3.39
3.37
3.16
3.15
3.14
3.13
3.09
3.08
3.08
3.07
3.07
3.06
2.32
2.31
2.30
2.13
1.56
1.54
1.48
1.48
1.46
1.46
1.12
1.10
1.09
CH3
O
CH3
H3C
N
NH
NH
O
O
CH3
1h
0.85
0.93
1.00
2.10
2.09
2.17
1.94
1.94
5.97
2.03
1.86
6.20
8.0
7.5
7.0
6.5
6.0
5.5
5.0
4.5
4.0
3.5
3.0
2.5
2.0
1.5
1.0
f1 (мд)

Figure S 19.

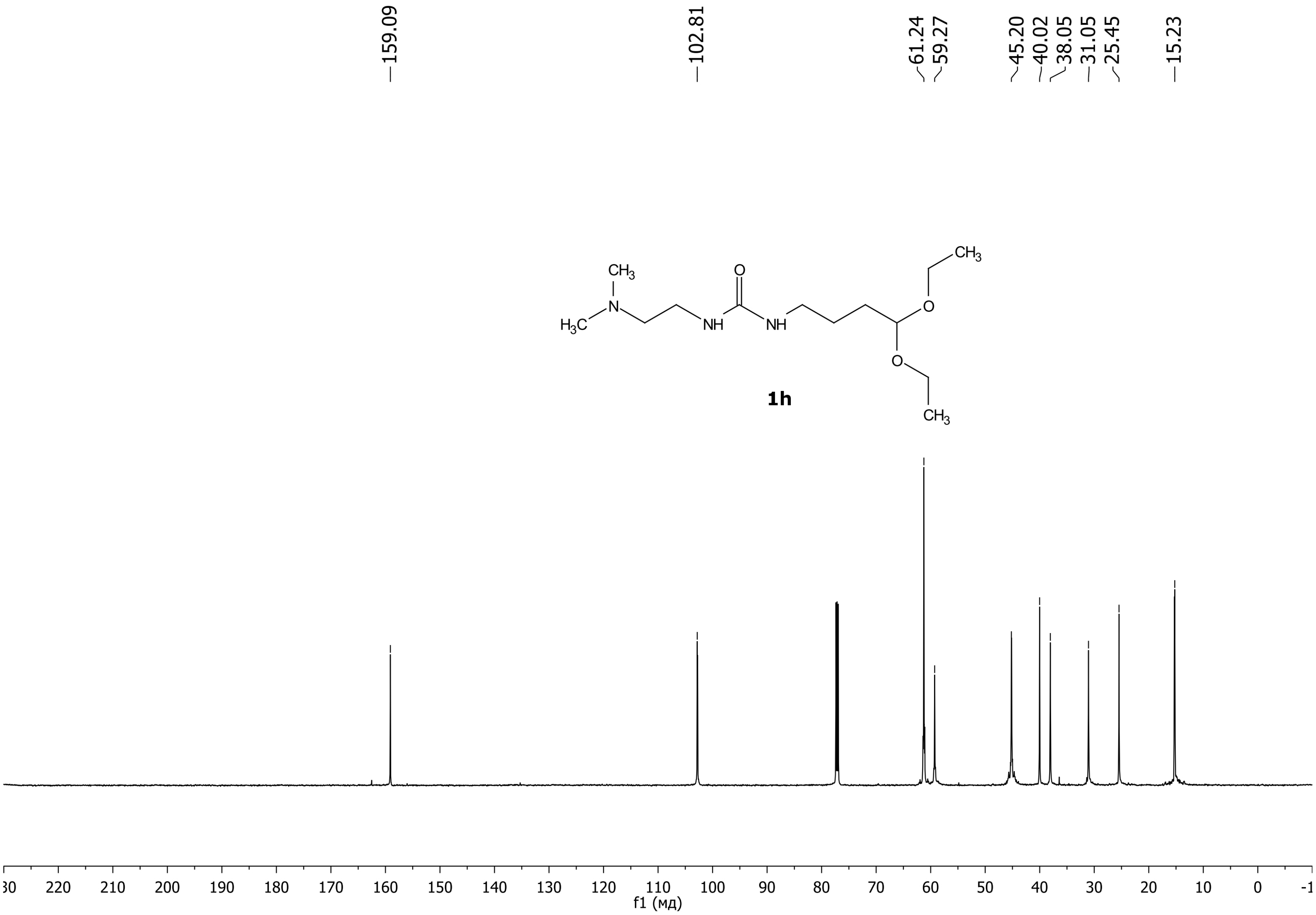
159.09
102.81
61.24
59.27
45.20
40.02
38.05
31.05
25.45
15.23
CH3
O
CH3
H3C
N
NH
NH
O
O
1h
CH3
f1 (мд)

Figure S 20.

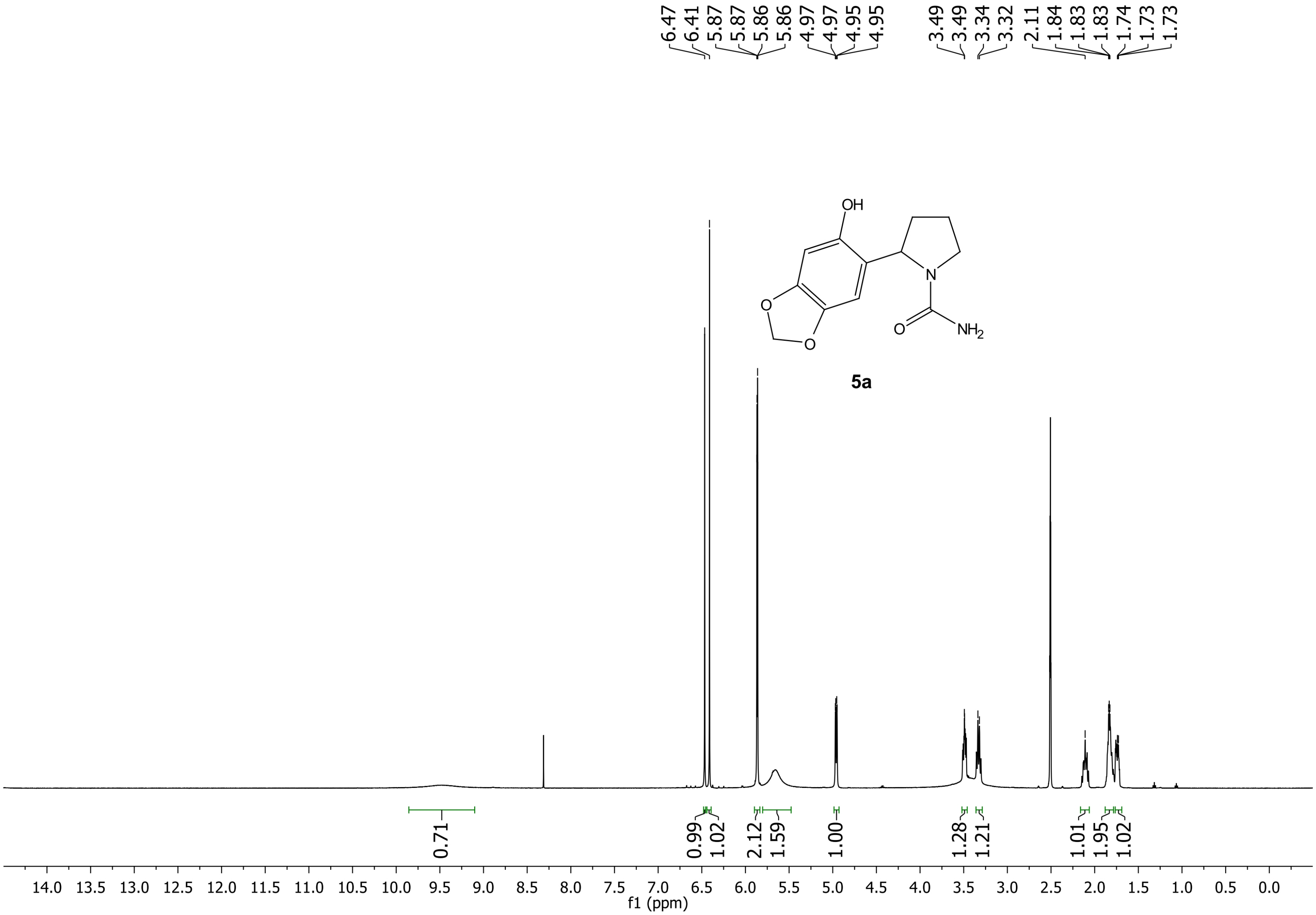
6.47
6.41
5.87
5.87
5.86
5.86
4.97
4.97
4.95
4.95
3.49
3.49
3.34
3.32
2.11
1.84
1.83
1.83
1.74
1.73
1.73
OH
O
O
N
O
NH2
5a
0.71
0.99
1.02
2.12
1.59
1.00
1.28
1.21
1.01
1.95
1.02
14.0 13.5 13.0 12.5 12.0 11.5 11.0 10.5 10.0 9.5 9.0 8.5 8.0 7.5 7.0 6.5 6.0 5.5 5.0 4.5 4.0 3.5 3.0 2.5 2.0 1.5 1.0 0.5 0.0
f1 (ppm)

Figure S 21.

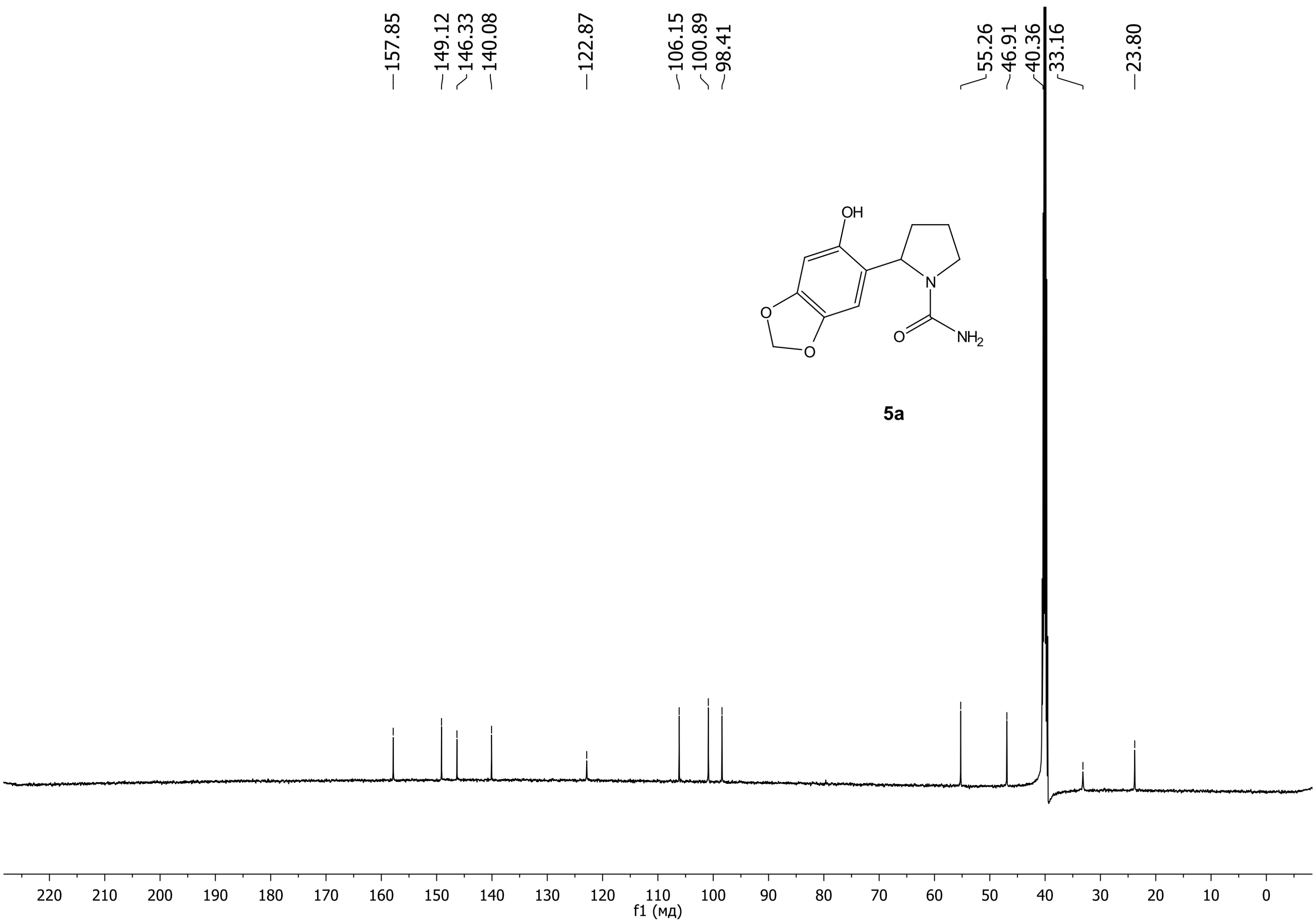

157.85
149.12
146.33
140.08
122.87
106.15
100.89
98.41
55.26
46.91
40.36
33.16
23.80
OH
N
O
O
O
NH2
5a
220 210 200 190 180 170 160 150 140 130 120 110 100 90 80 70 60 50 40 30 20 10 0
f1 (мд)

Figure S 22.

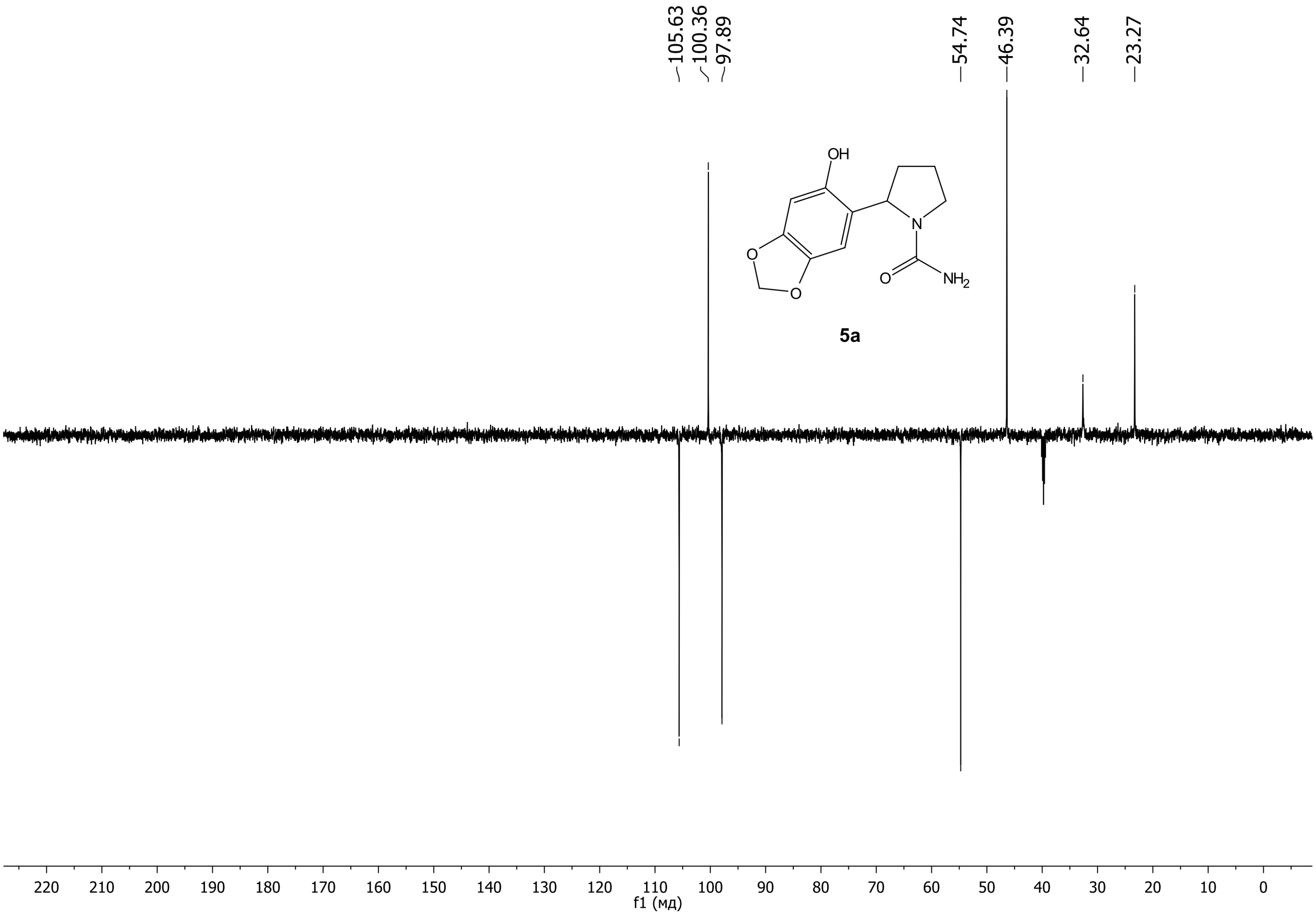
105.63
100.36
97.89
54.74
46.39
32.64
23.27
OH
O
O
N
O
NH2
5a
220 210 200 190 180 170 160 150 140 130 120 110 100 90 80 70 60 50 40 30 20 10 0
f1 (мд)

Figure S 23.

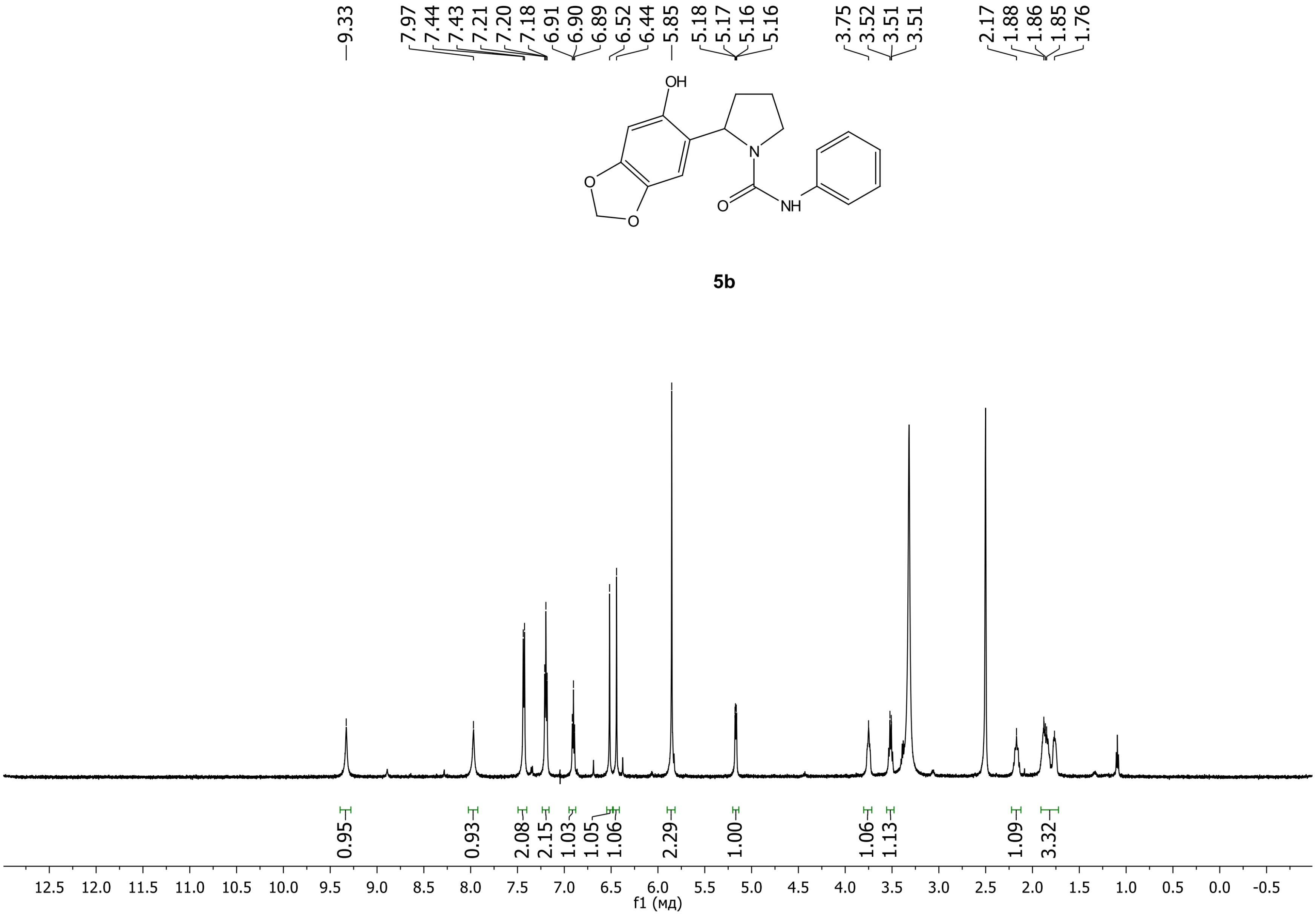

5b

Figure S 24.

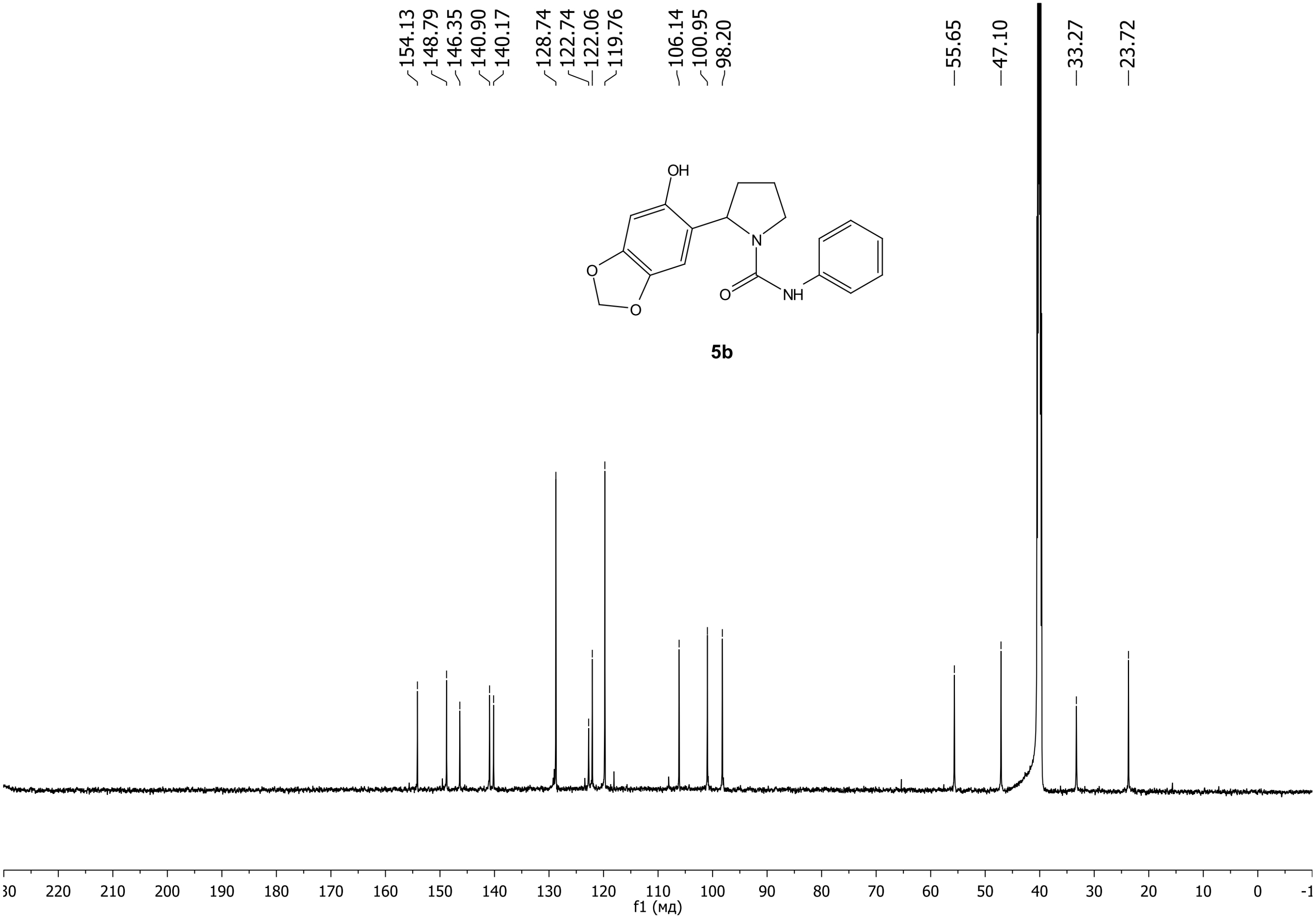
154.13
148.79
146.35
140.90
140.17
128.74
122.74
122.06
119.76
106.14
100.95
98.20
55.65
47.10
33.27
23.72
OH
O
O
N
O
NH
5b
30
220
210
200
190
180
170
160
150
140
130
120
110
100
90
80
70
60
50
40
30
20
10
0
-1
f1 (мд)

Figure S 25.

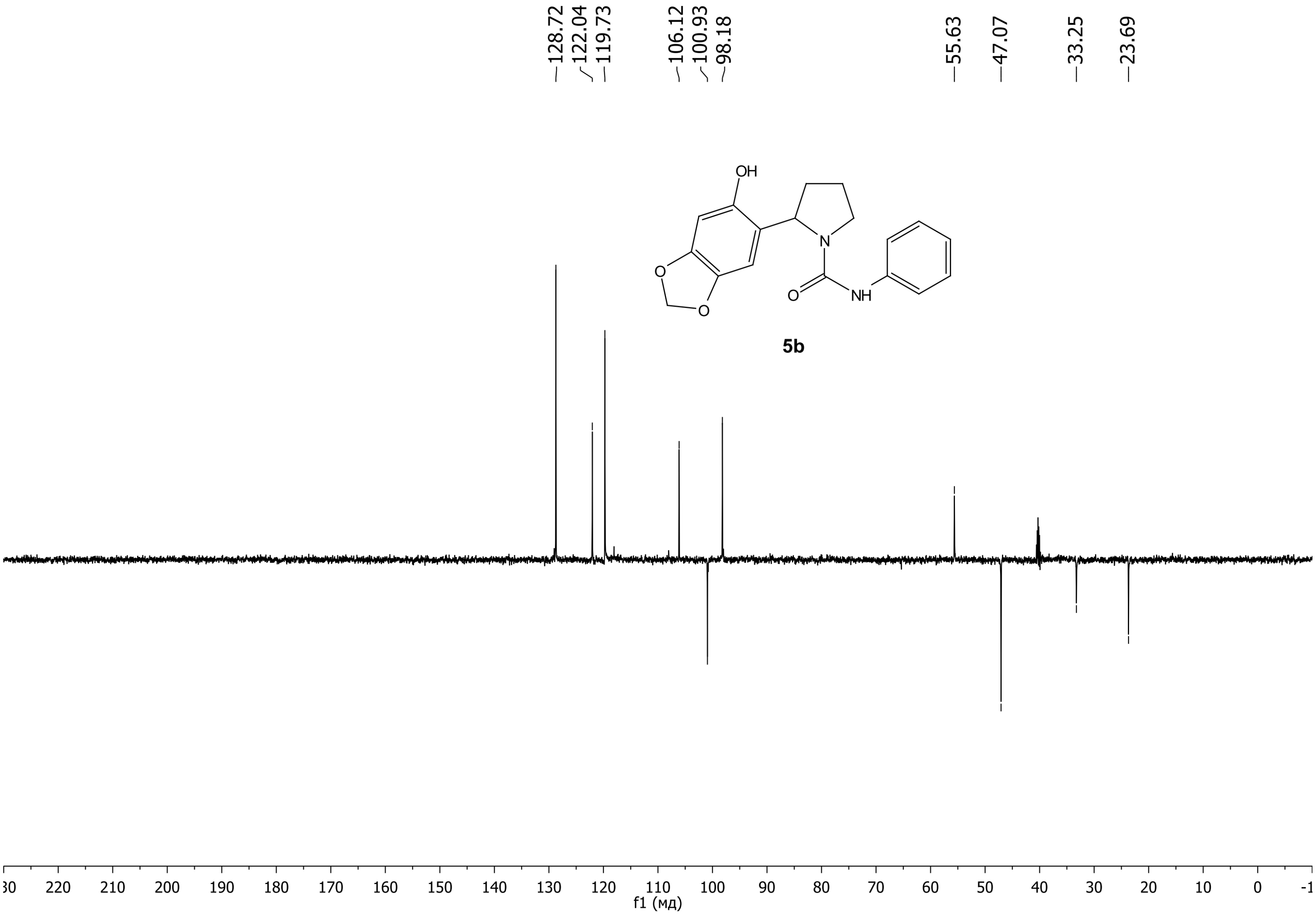
128.72
122.04
119.73
106.12
100.93
98.18
55.63
47.07
33.25
23.69
OH
O
O
N
O
NH
5b
f1 (мд)

Figure S 26.

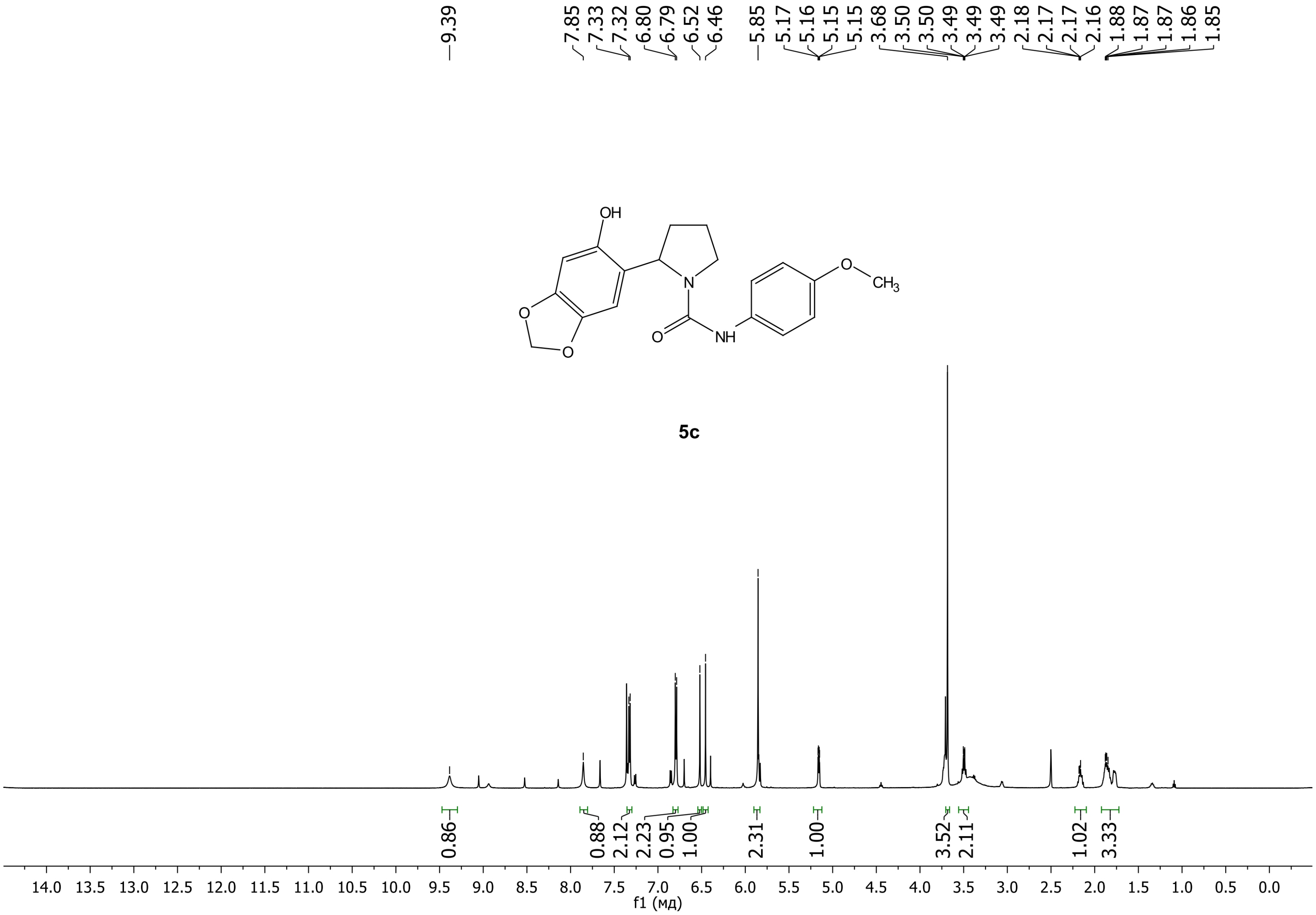
9.39
7.85
7.33
7.32
6.80
6.79
6.52
6.46
5.85
5.17
5.16
5.15
5.15
3.68
3.50
3.50
3.49
3.49
3.49
2.18
2.17
2.17
2.16
1.88
1.87
1.87
1.86
1.85
OH
N
O
CH3
O
O
O
NH
5c
0.86
0.88
2.12
2.23
0.95
1.00
2.31
1.00
3.52
2.11
1.02
3.33
14.0 13.5 13.0 12.5 12.0 11.5 11.0 10.5 10.0 9.5 9.0 8.5 8.0 7.5 7.0 6.5 6.0 5.5 5.0 4.5 4.0 3.5 3.0 2.5 2.0 1.5 1.0 0.5 0.0
f1 (мд)

Figure S 27.

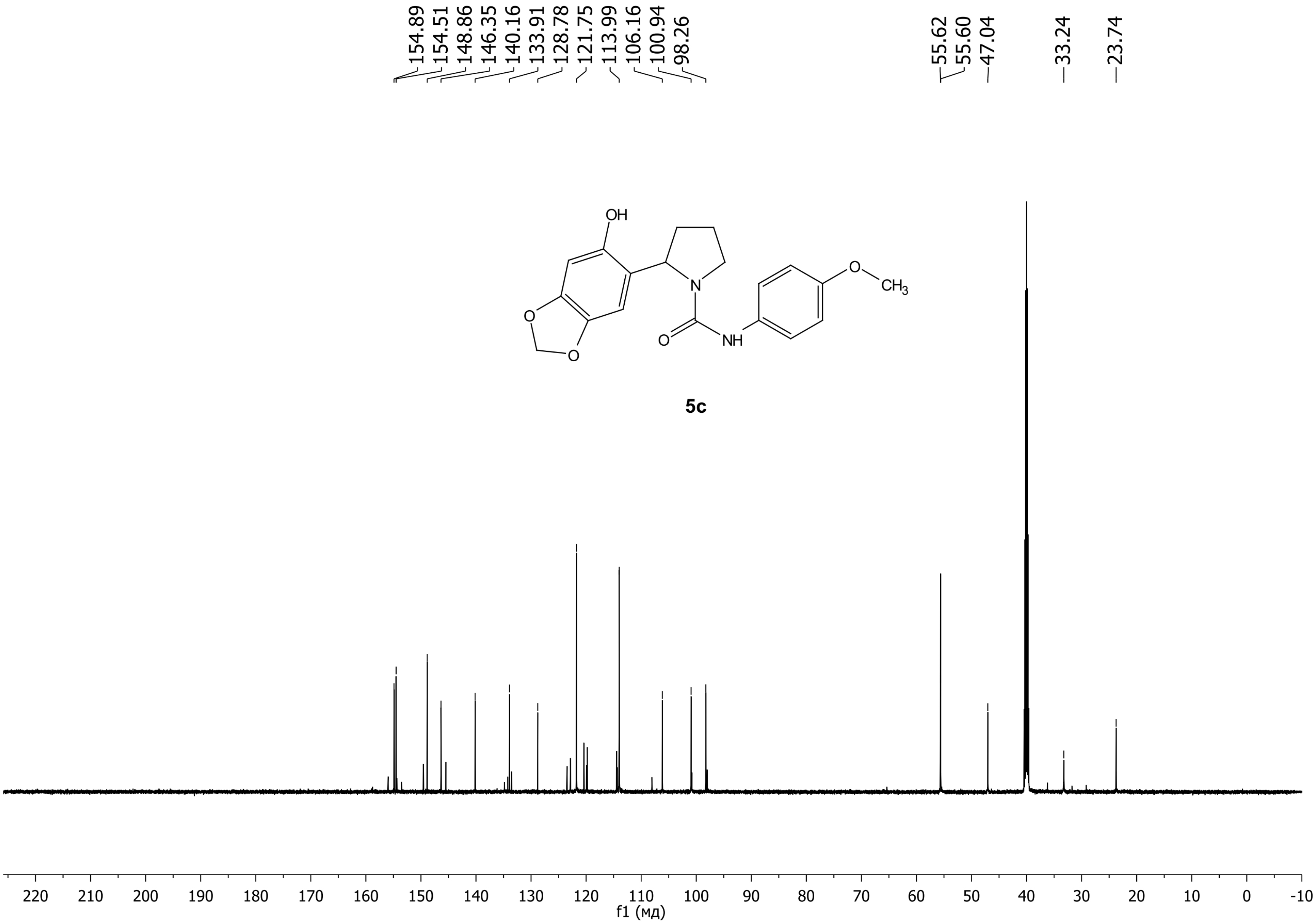
154.89
154.51
148.86
146.35
140.16
133.91
128.78
121.75
113.99
106.16
100.94
98.26
55.62
55.60
47.04
33.24
23.74
OH
O
O
N
O
NH
O
CH3
5c
220 210 200 190 180 170 160 150 140 130 120 110 100 90 80 70 60 50 40 30 20 10 0 -10
f1 (мд)

Figure S 28.

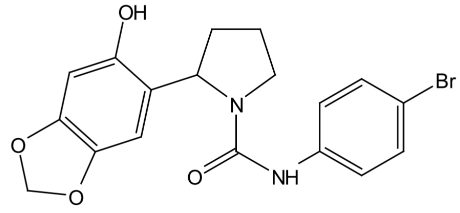

5d

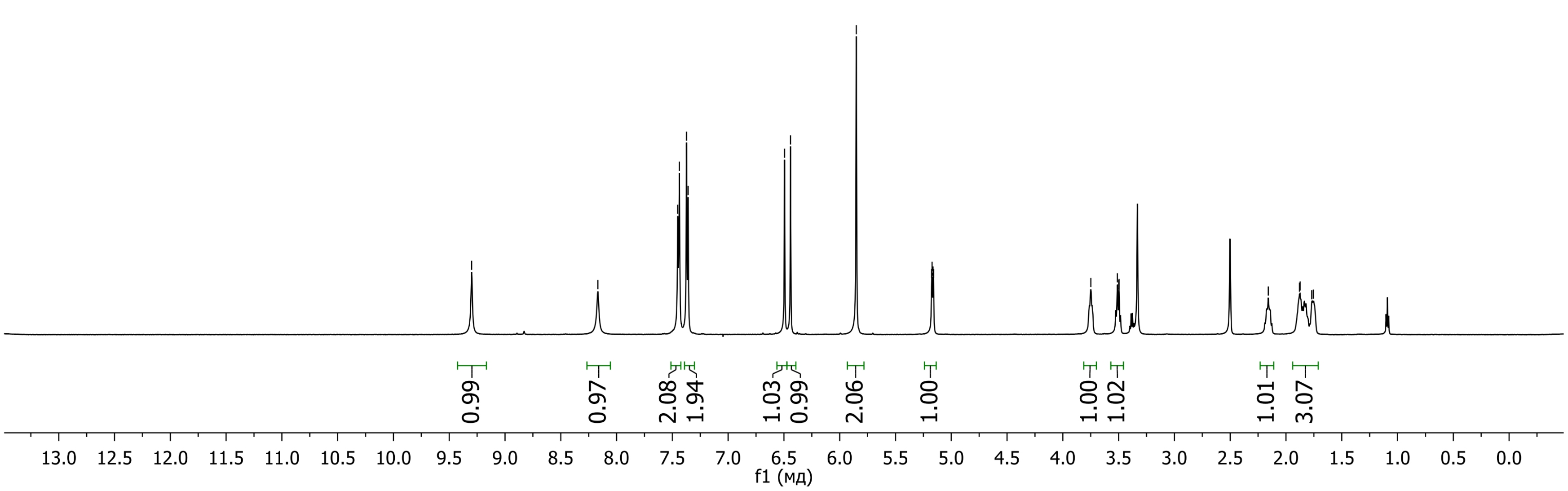

Figure S 29.

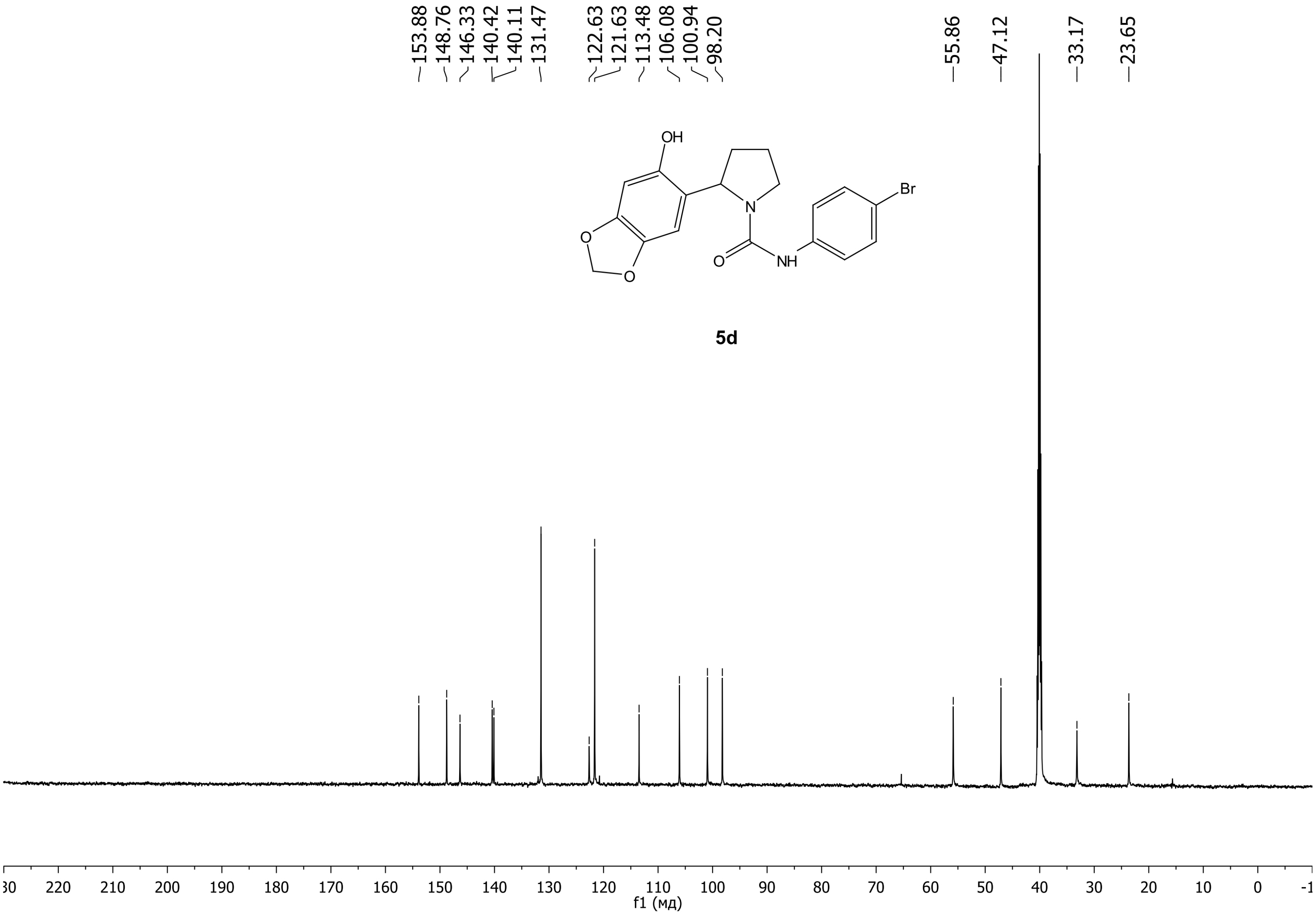
153.88
148.76
146.33
140.42
140.11
131.47
122.63
121.63
113.48
106.08
100.94
98.20
55.86
47.12
33.17
23.65
OH
O
O
N
Br
O
NH
5d
f1 (мд)

Figure S 30.

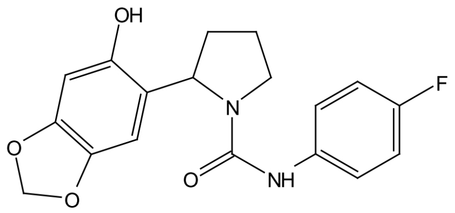

5e

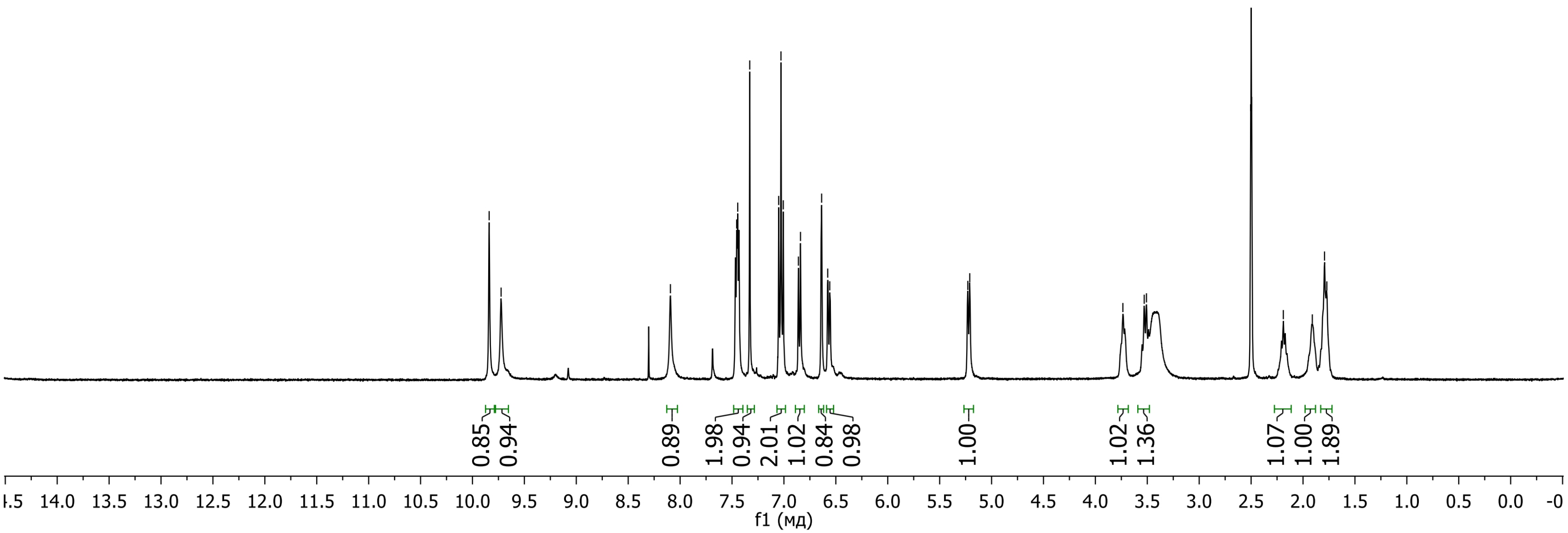

Figure S 31.

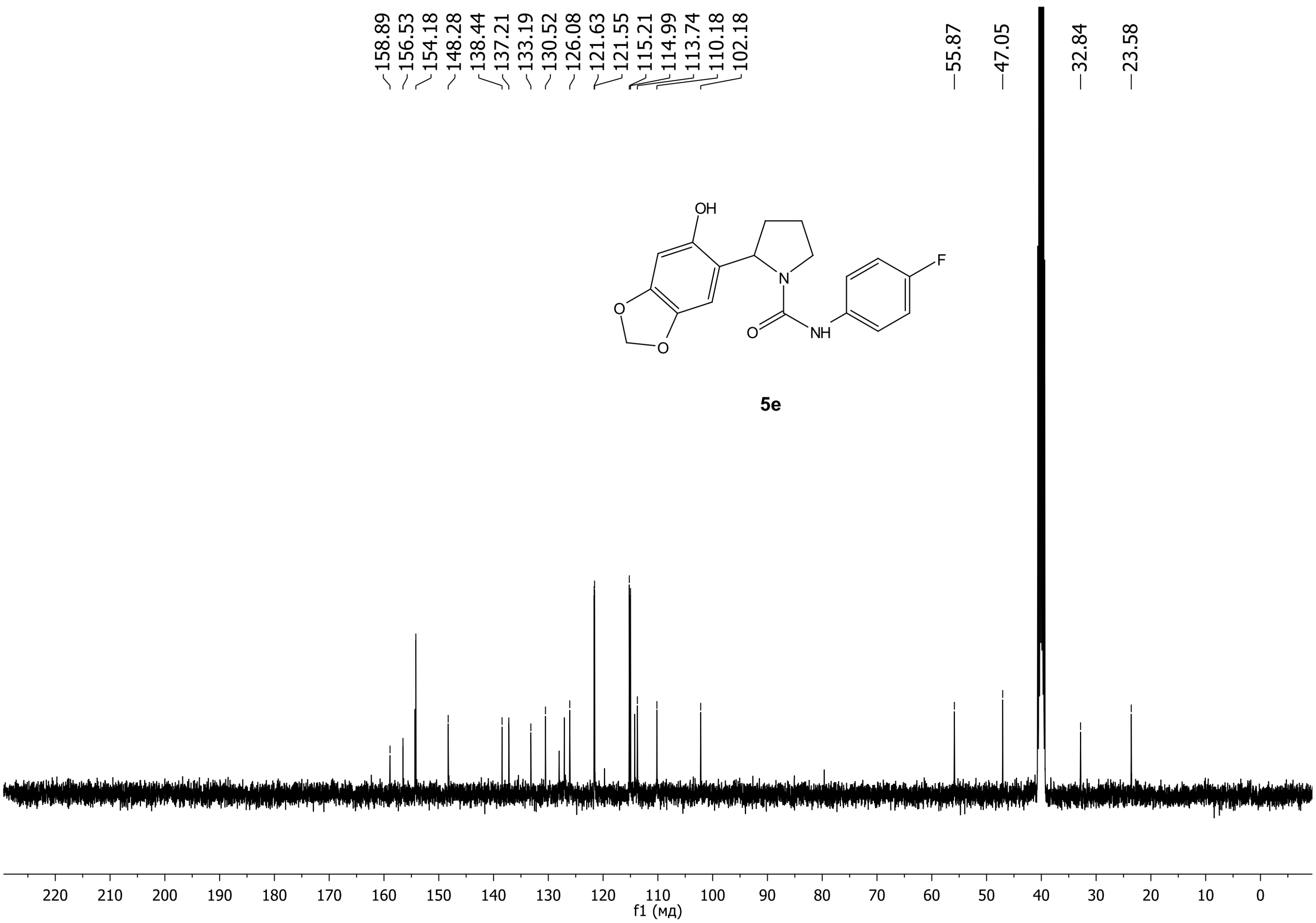
158.89
156.53
154.18
148.28
138.44
137.21
133.19
130.52
126.08
121.63
121.55
115.21
114.99
113.74
110.18
102.18
55.87
47.05
32.84
23.58
OH
F
O
N
O
O
NH
5e
220
210
200
190
180
170
160
150
140
130
120
110
100
90
80
70
60
50
40
30
20
10
0
f1 (мд)

Figure S 32.

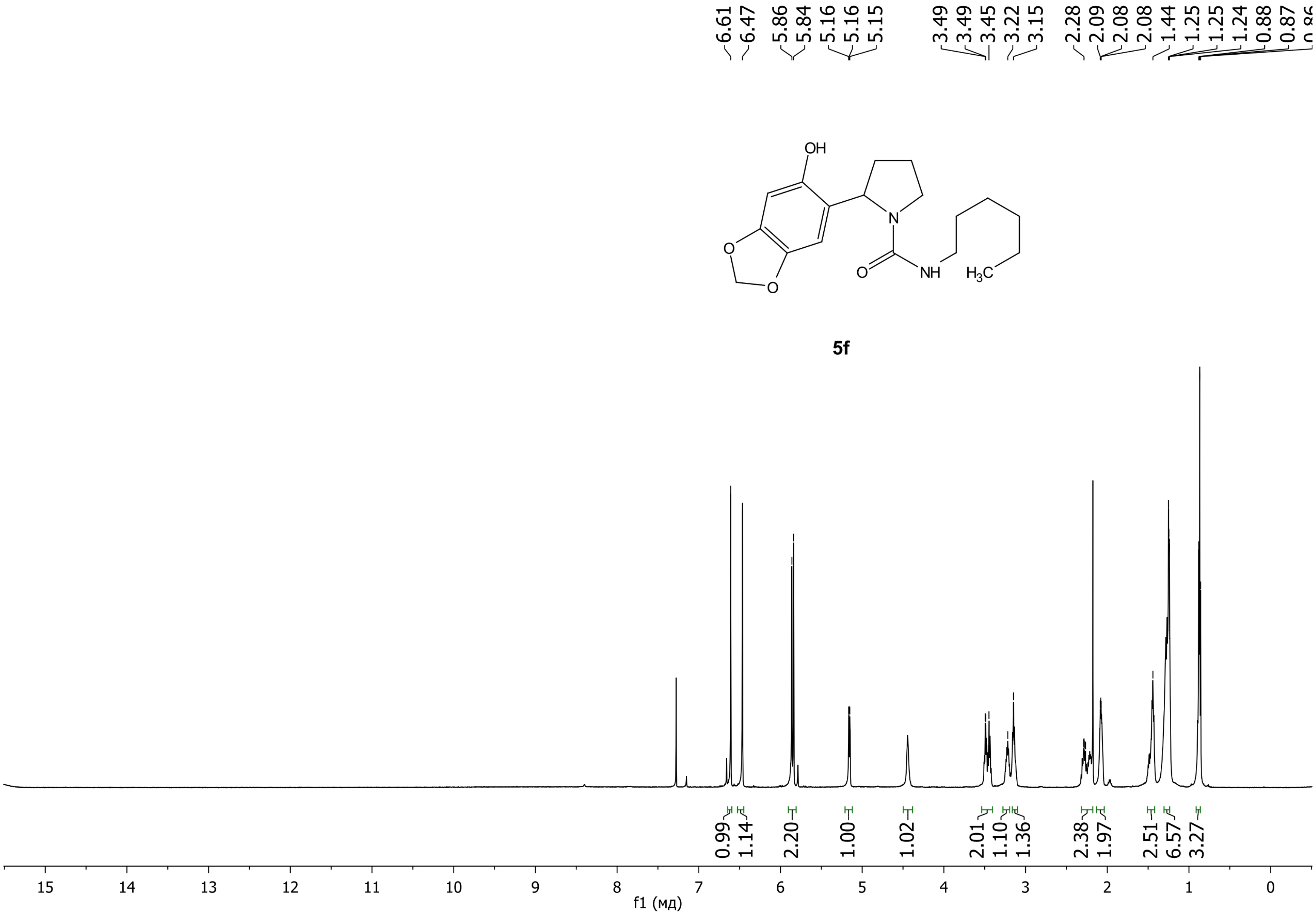

5f

Figure S 33.

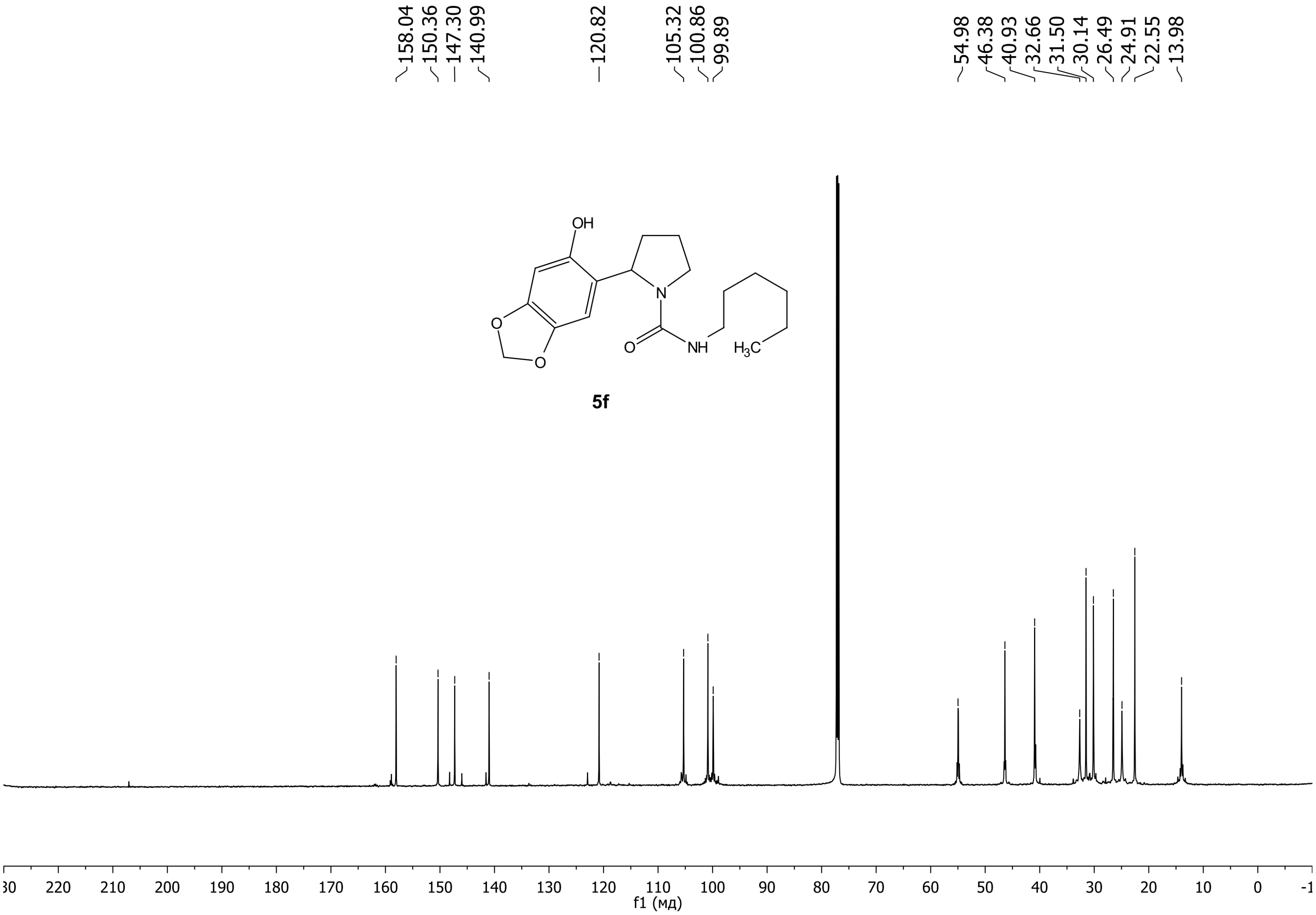
158.04
150.36
147.30
140.99
120.82
105.32
100.86
99.89
54.98
46.38
40.93
32.66
31.50
30.14
26.49
24.91
22.55
13.98
OH
N
O
O
O
NH
H_3C
5f
f1 (мд)

Figure S 34.

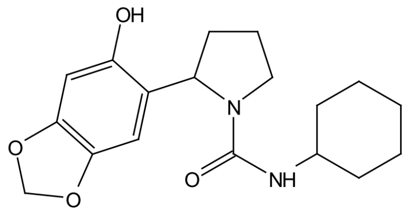

5g

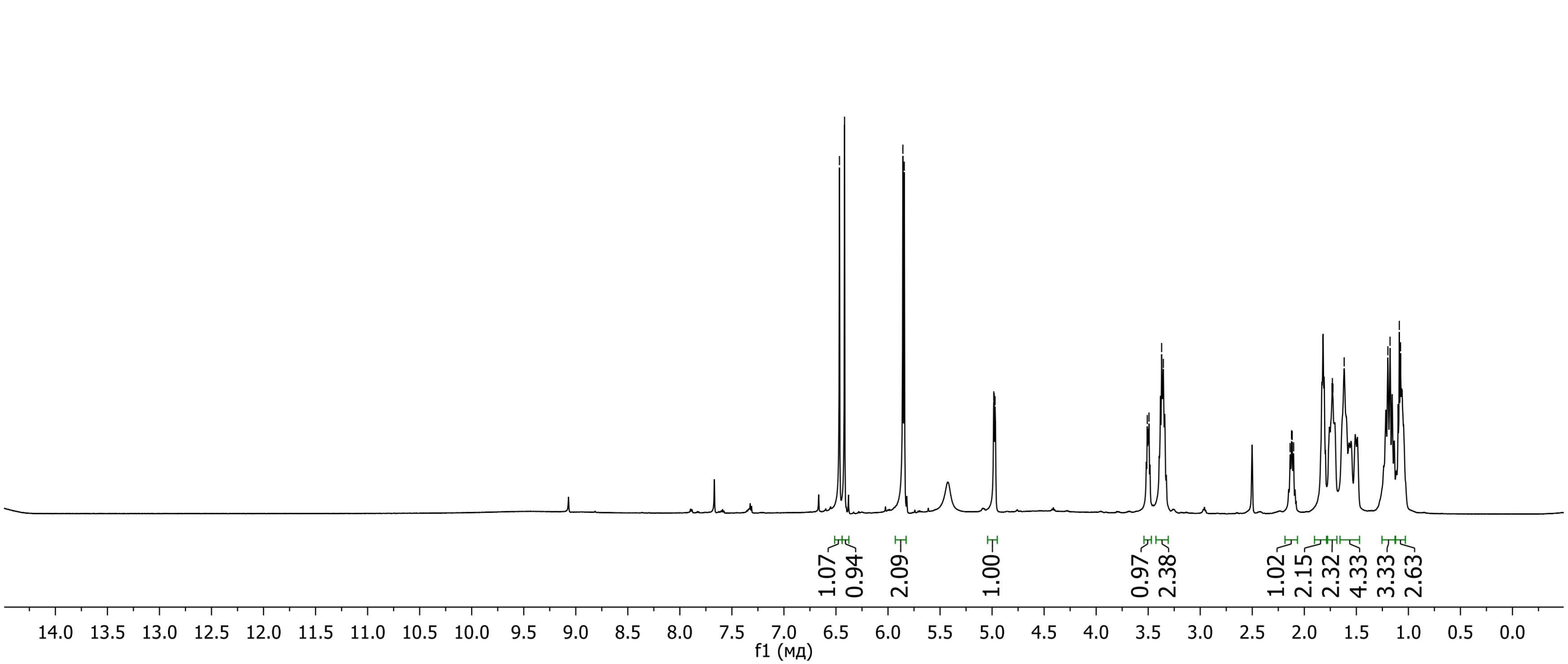

Figure S 35.

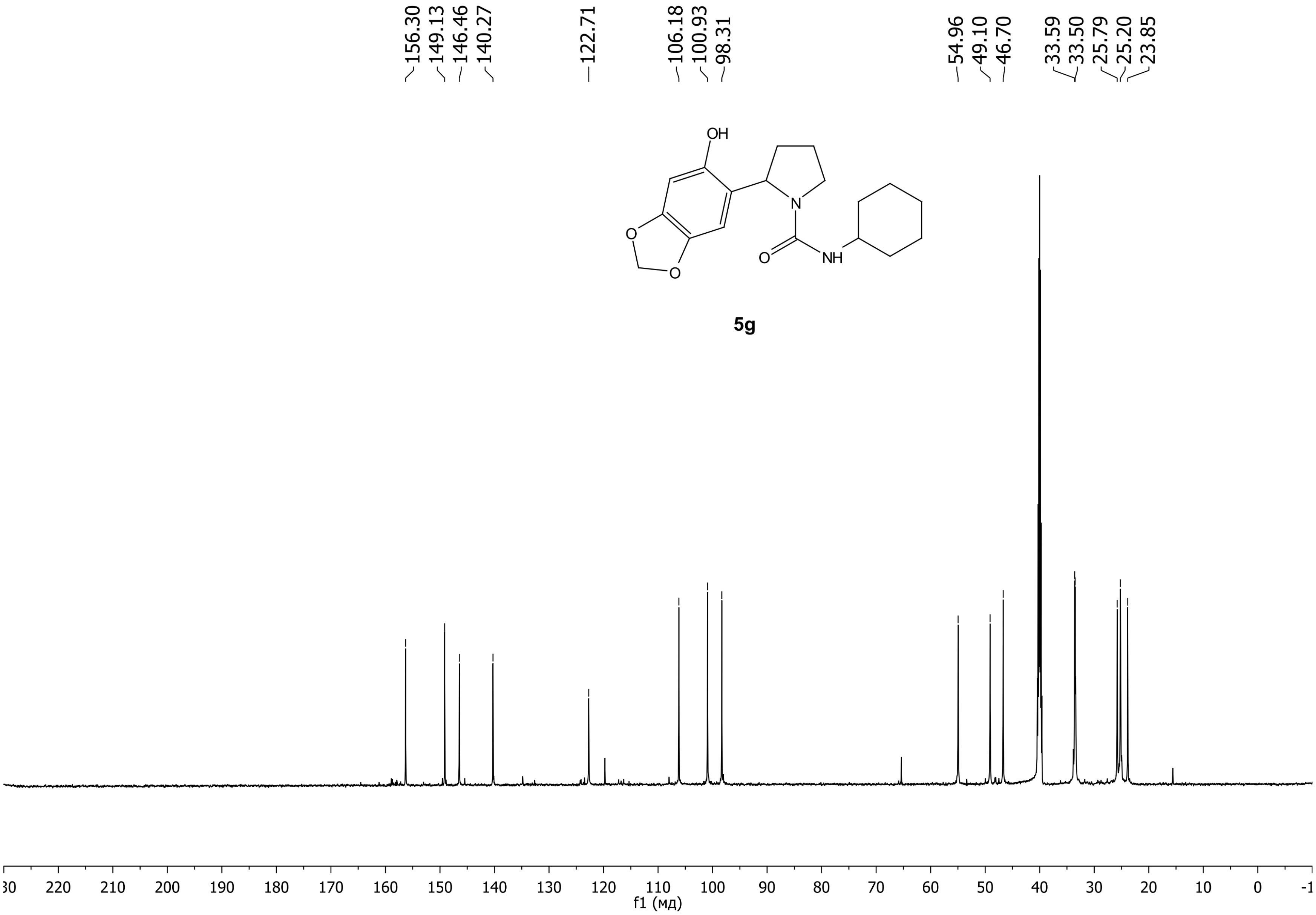
156.30
149.13
146.46
140.27
122.71
106.18
100.93
98.31
54.96
49.10
46.70
33.59
33.50
25.79
25.20
23.85
OH
O
O
N
O
NH
5g
220 210 200 190 180 170 160 150 140 130 120 110 100 90 80 70 60 50 40 30 20 10 0
f1 (мд)

Figure S 36.

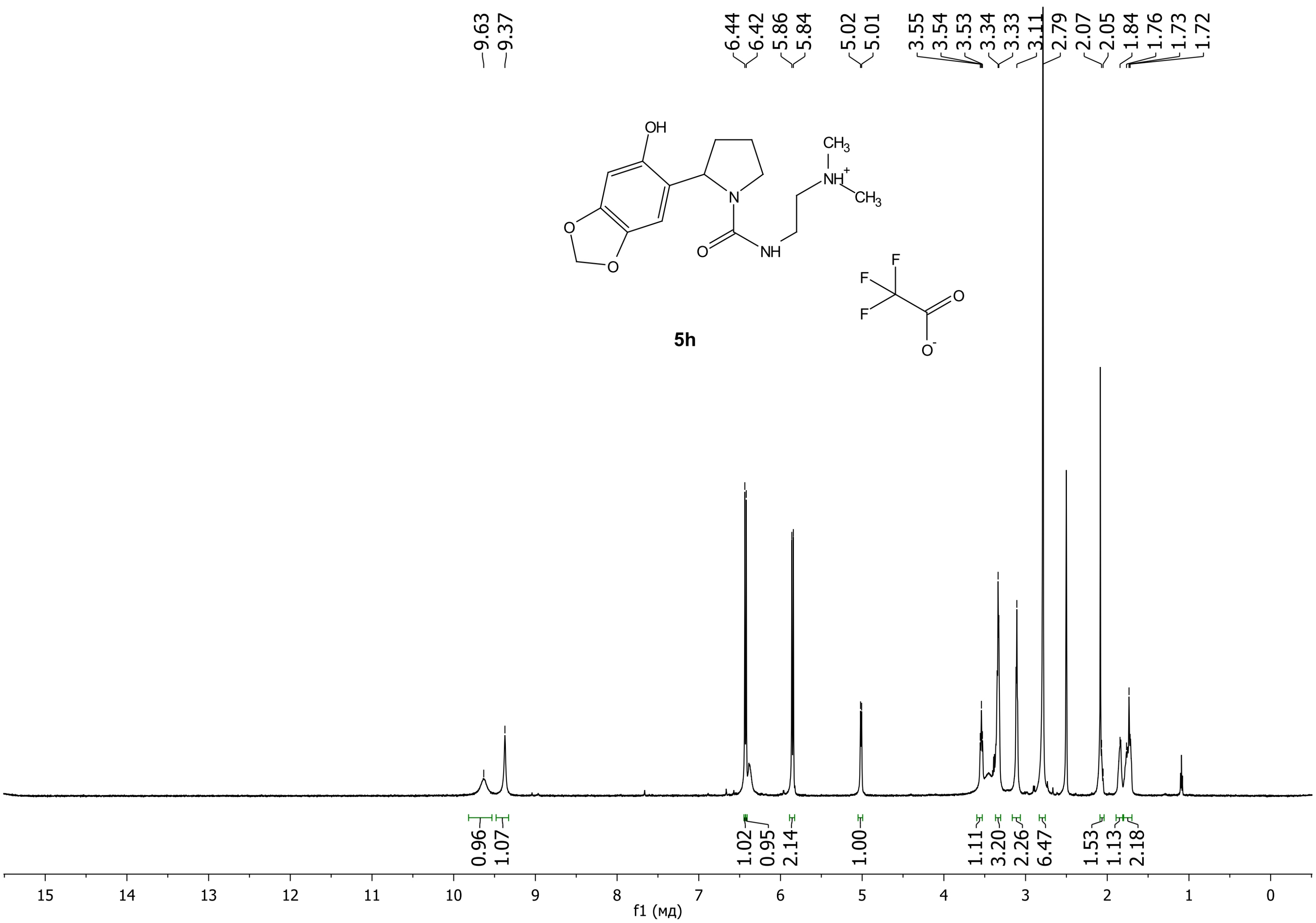
9.63
9.37
6.44
6.42
5.86
5.84
5.02
5.01
3.55
3.54
3.53
3.34
3.33
3.11
2.79
2.07
2.05
1.84
1.76
1.73
1.72
OH
CH3
NH+
CH3
N
O
O
O
NH
F
F
F
O
O-
5h
0.96
1.07
1.02
0.95
2.14
1.00
1.11
3.20
2.26
6.47
1.53
1.13
2.18
15
14
13
12
11
10
9
8
7
6
5
4
3
2
1
0
f1 (мд)

Figure S 37.

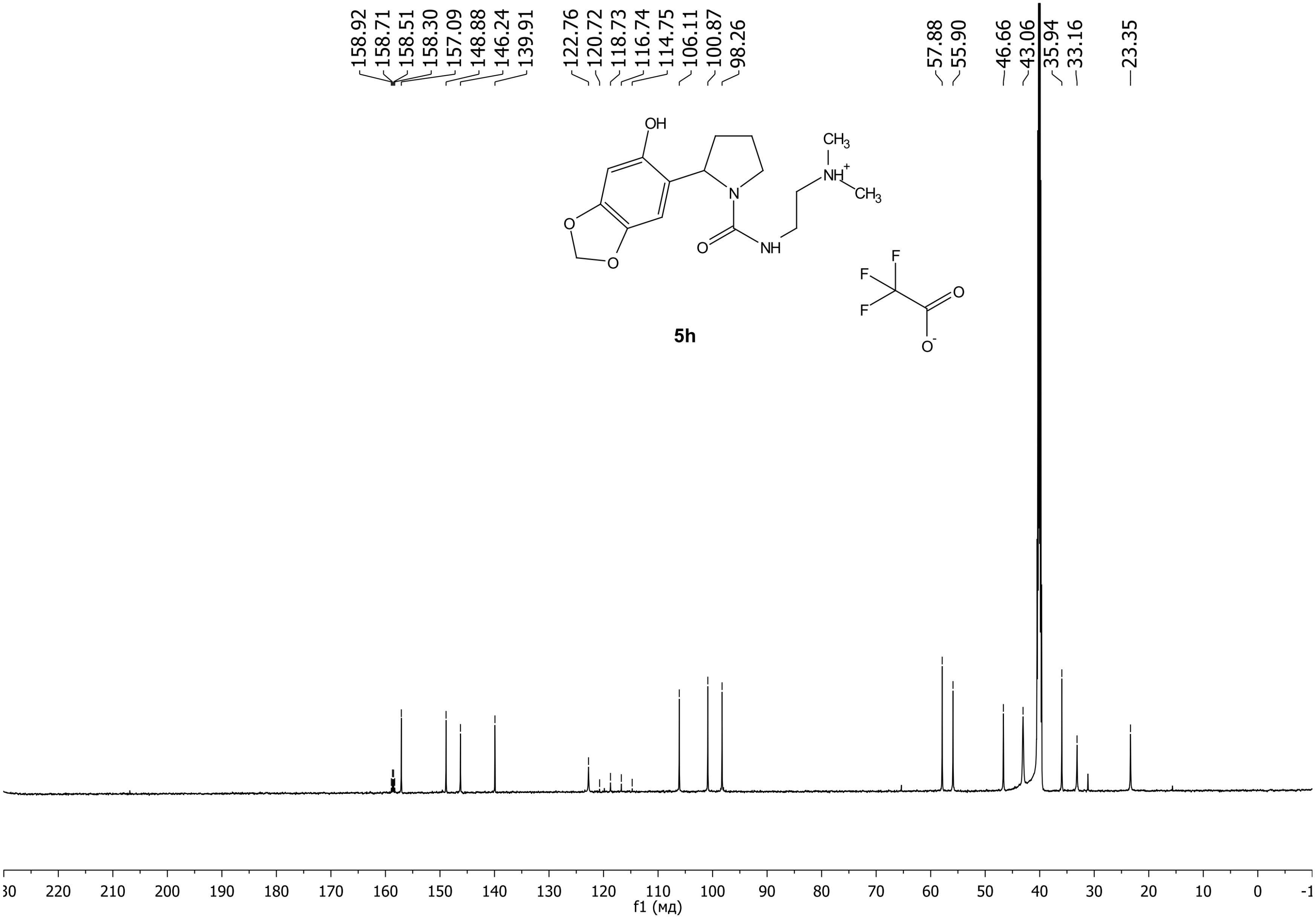
158.92
158.71
158.51
158.30
157.09
148.88
146.24
139.91
122.76
120.72
118.73
116.74
114.75
106.11
100.87
98.26
57.88
55.90
46.66
43.06
35.94
33.16
23.35
OH
O
O
N
O
NH
CH3
NH+
CH3
F
F
F
O
O-
5h
f1 (мд)

Figure S 38.

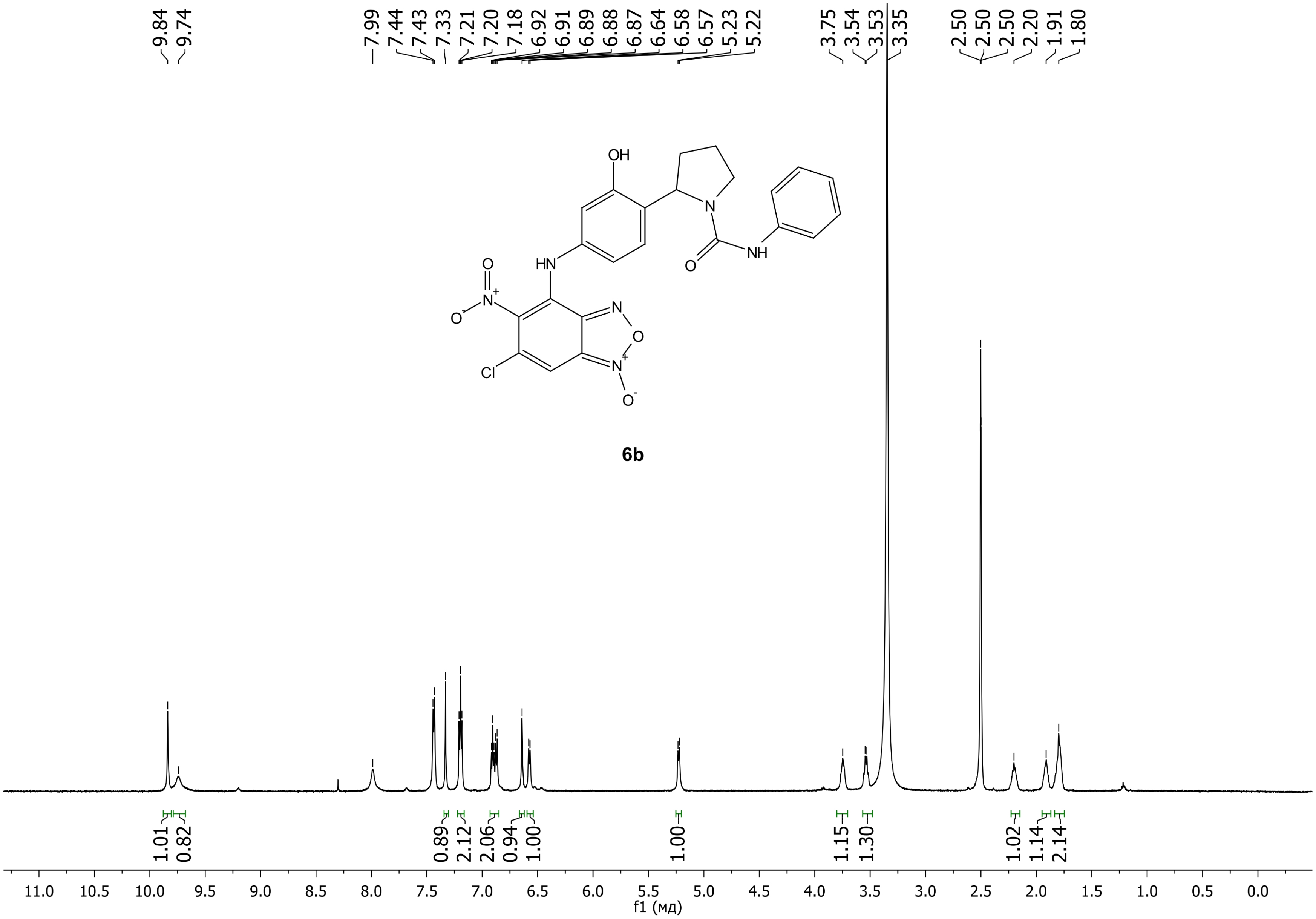

9.84
9.74
7.99
7.44
7.43
7.33
7.21
7.20
7.18
6.92
6.91
6.89
6.88
6.87
6.64
6.58
6.57
5.23
5.22
3.75
3.54
3.53
3.35
2.50
2.50
2.50
2.20
1.91
1.80
OH
N
HN
NH
O
N+
O-
O
N
O
N+
O-
Cl
6b
1.01
0.82
0.89
2.12
2.06
0.94
1.00
1.00
1.15
1.30
1.02
1.14
2.14
11.0
10.5
10.0
9.5
9.0
8.5
8.0
7.5
7.0
6.5
6.0
5.5
5.0
4.5
4.0
3.5
3.0
2.5
2.0
1.5
1.0
0.5
0.0
f1 (мд)

Figure S 39.

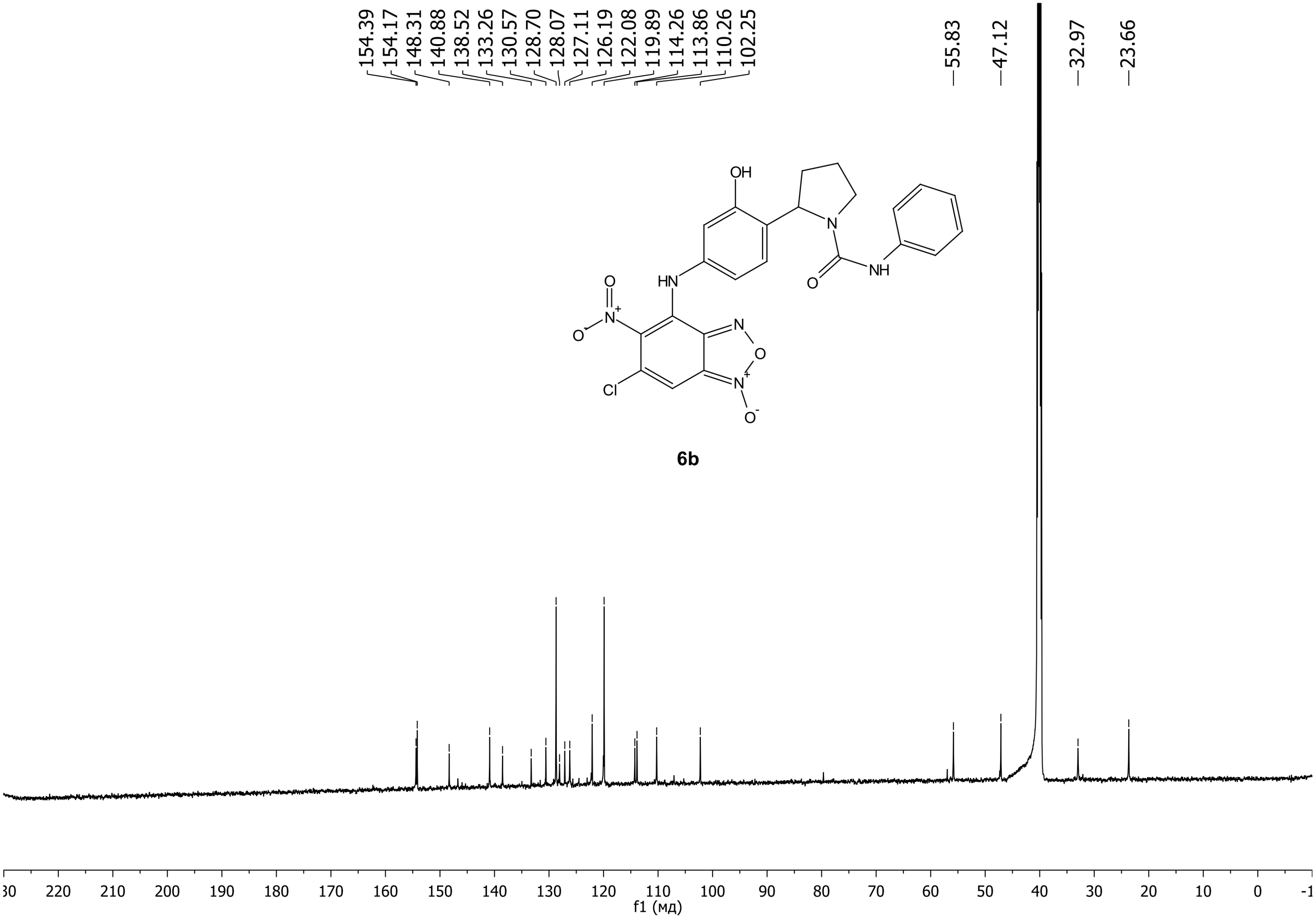
154.39
154.17
148.31
140.88
138.52
133.26
130.57
128.70
128.07
127.11
126.19
122.08
119.89
114.26
113.86
110.26
102.25
55.83
47.12
32.97
23.66
OH
N
HN
NH
O
O
N+
O-
N
O
N+
O-
Cl
6b
f1 (мд)

Figure S 40.

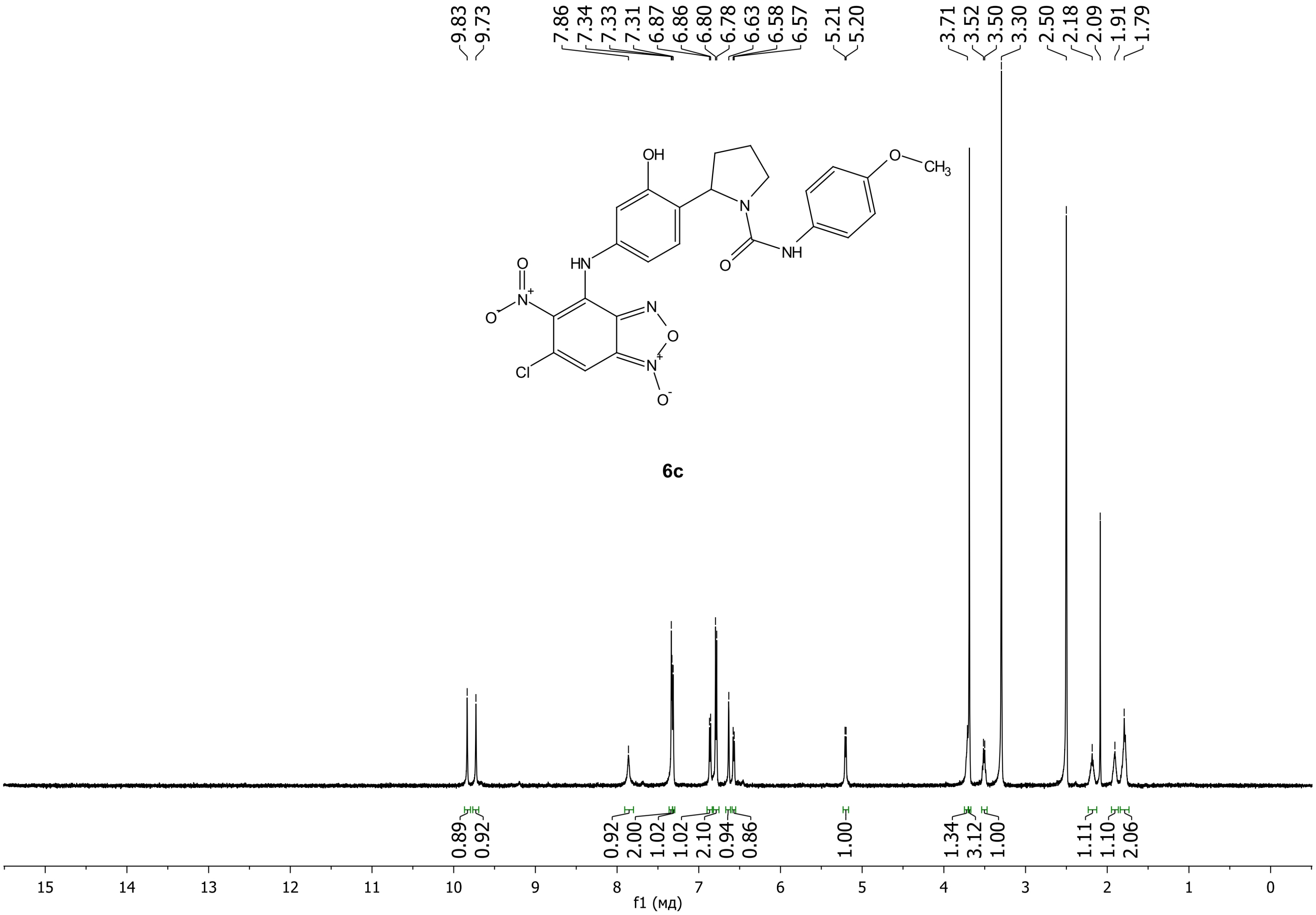

6c

Figure S 41.

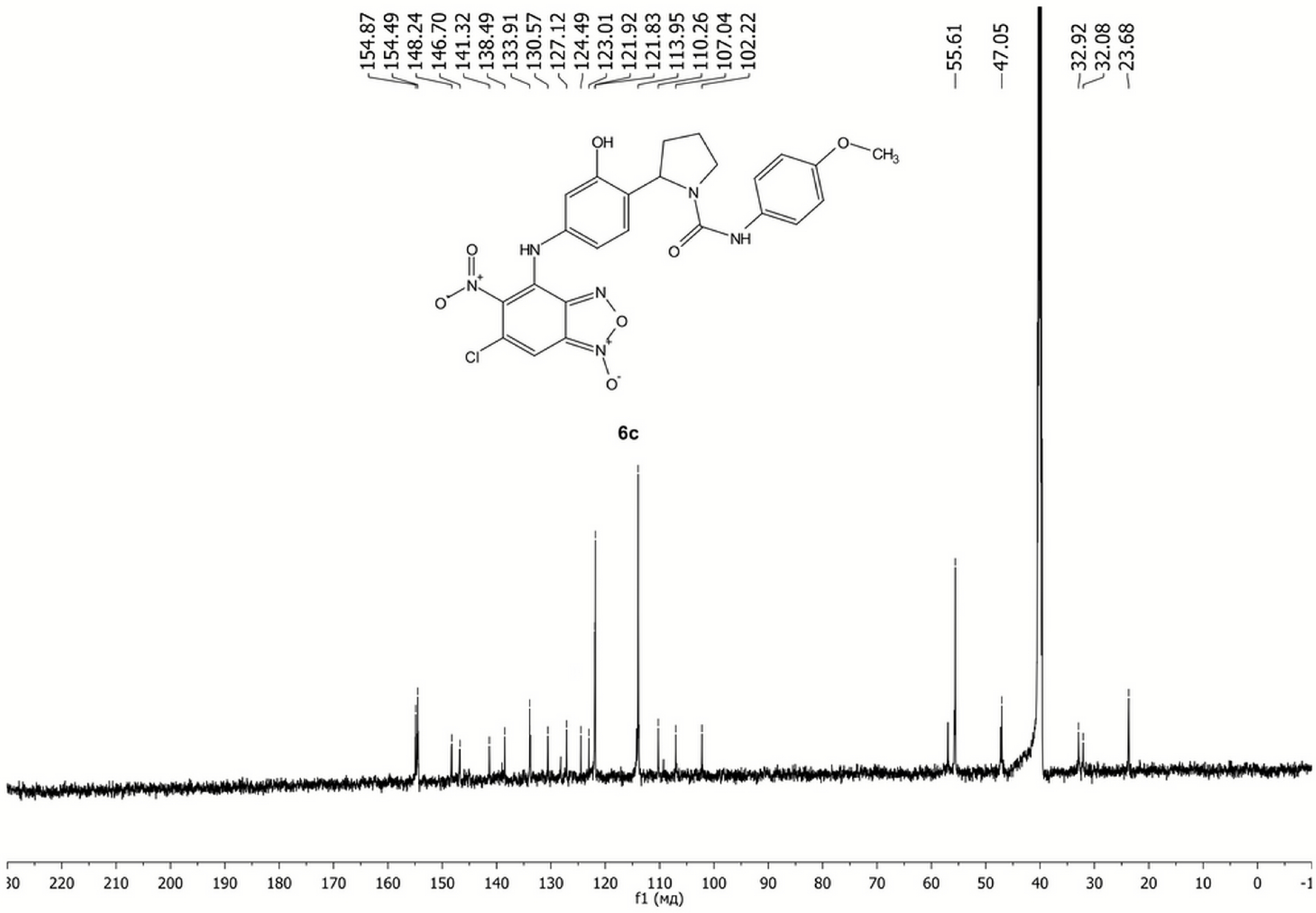
154.87
154.49
148.24
146.70
141.32
138.49
133.91
130.57
127.12
124.49
123.01
121.92
121.83
113.95
110.26
107.04
102.22
55.61
47.05
32.92
32.08
23.68
6c
f1 (мд)

Figure S 42.

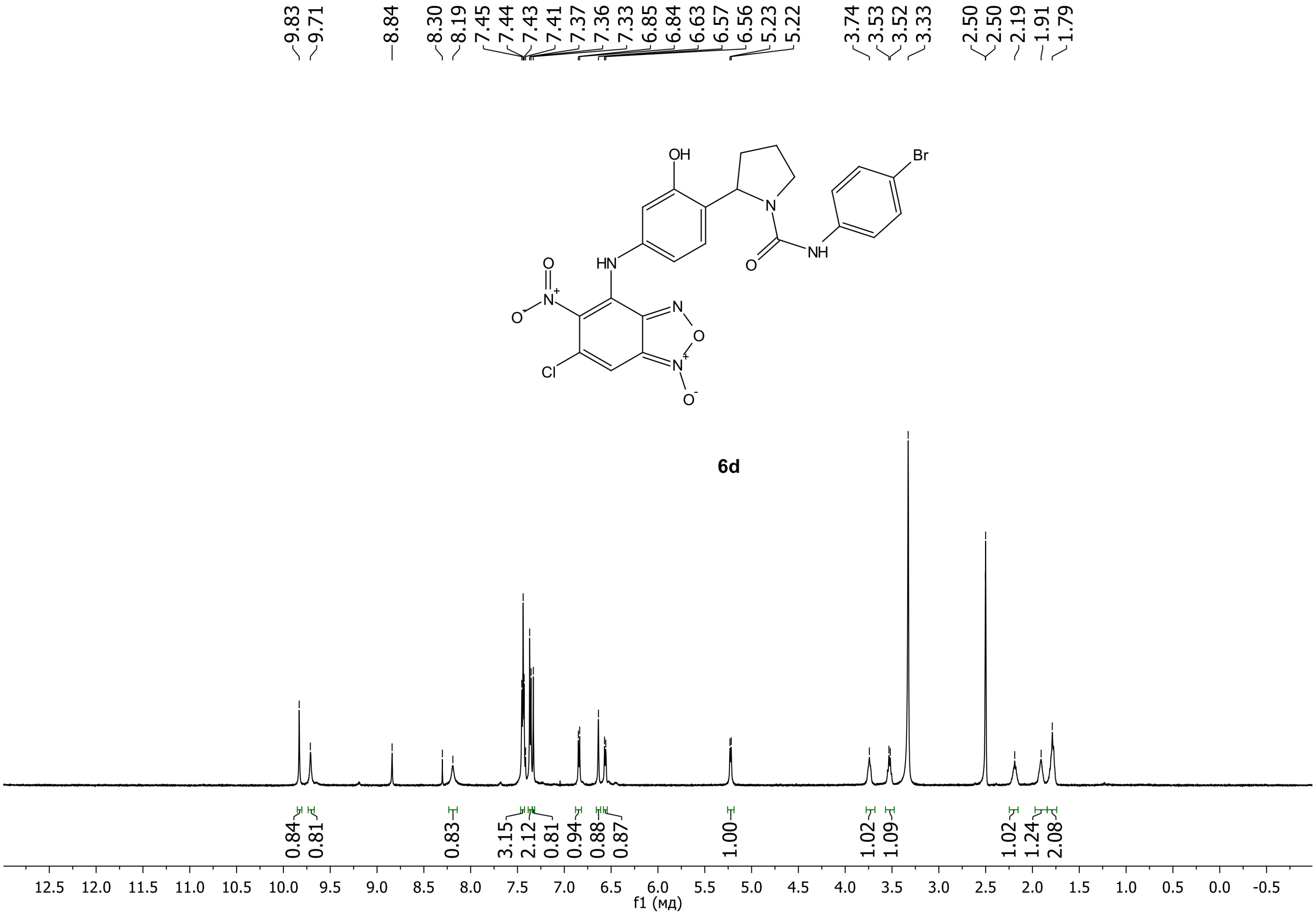
9.83
9.71
8.84
8.30
8.19
7.45
7.44
7.43
7.41
7.37
7.36
7.33
6.85
6.84
6.63
6.57
6.56
5.23
5.22
3.74
3.53
3.52
3.33
2.50
2.50
2.19
1.91
1.79
OH
Br
N
NH
O
HN
O
N+
O-
N
O
N+
O-
Cl
6d
0.84
0.81
0.83
3.15
2.12
0.81
0.94
0.88
0.87
1.00
1.02
1.09
1.02
1.24
2.08
12.5 12.0 11.5 11.0 10.5 10.0 9.5 9.0 8.5 8.0 7.5 7.0 6.5 6.0 5.5 5.0 4.5 4.0 3.5 3.0 2.5 2.0 1.5 1.0 0.5 0.0 -0.5
f1 (мд)

Figure S 43.

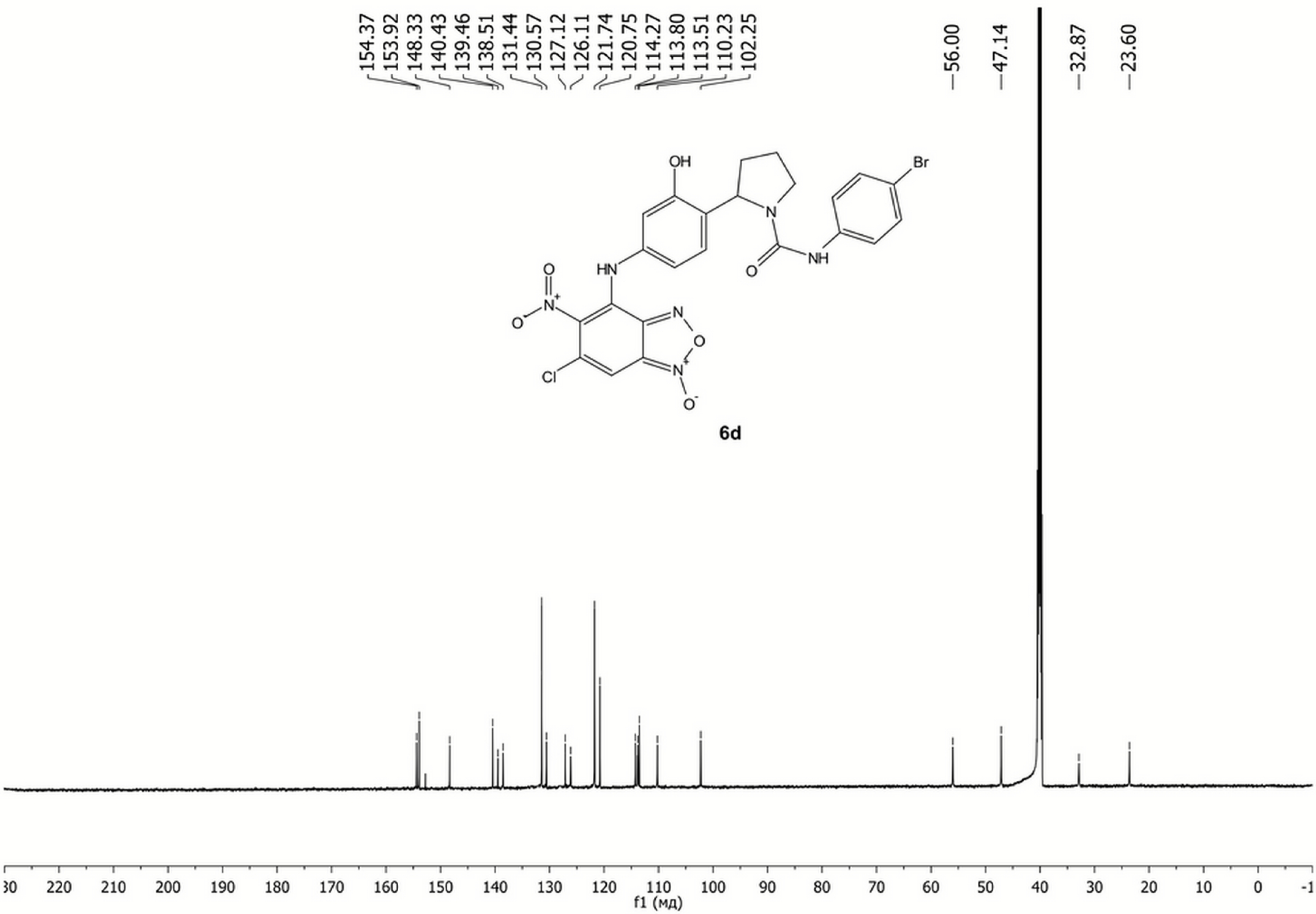
154.37
153.92
148.33
140.43
139.46
138.51
131.44
130.57
127.12
126.11
121.74
120.75
114.27
113.80
113.51
110.23
102.25
56.00
47.14
32.87
23.60
OH
Br
N
HN
NH
O
N
O
Cl
6d
f1 (мд)

Figure S 44.

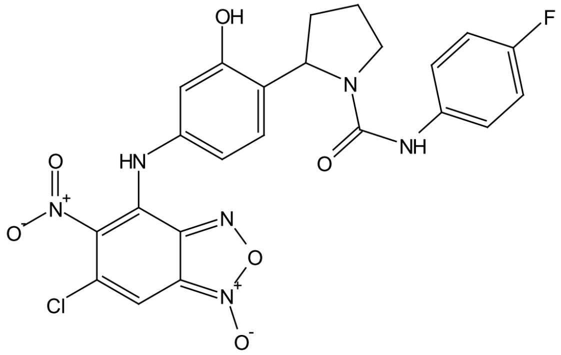

6e

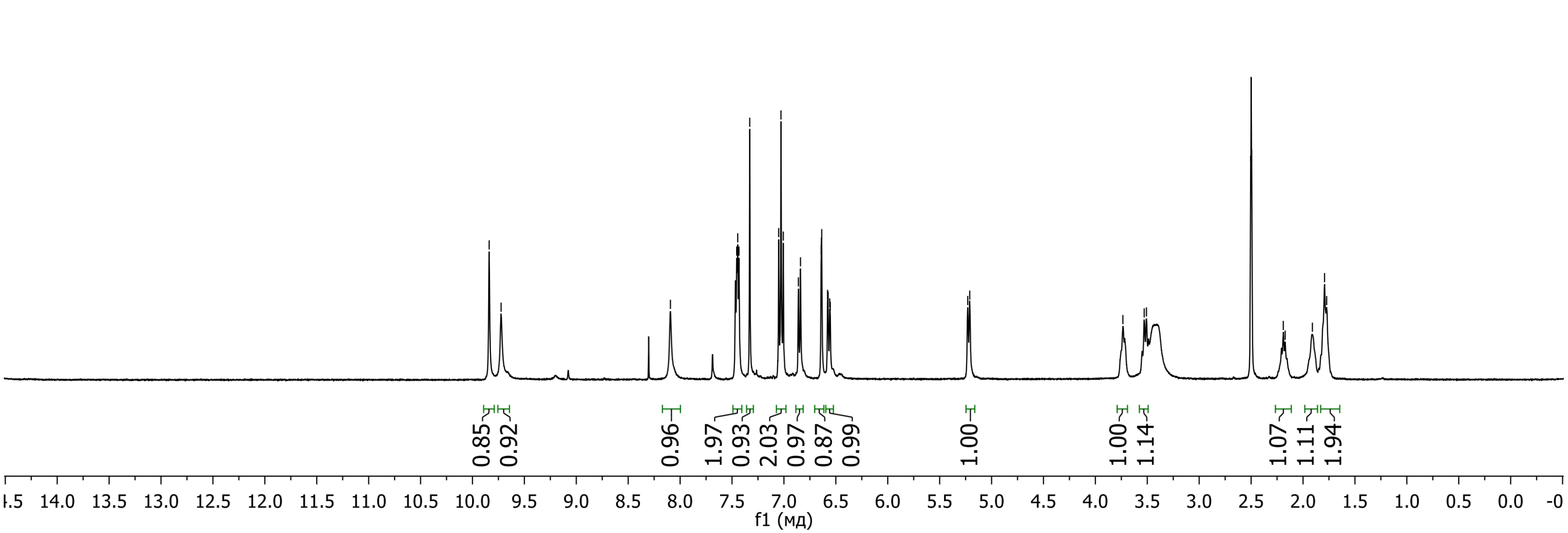

Figure S 45.

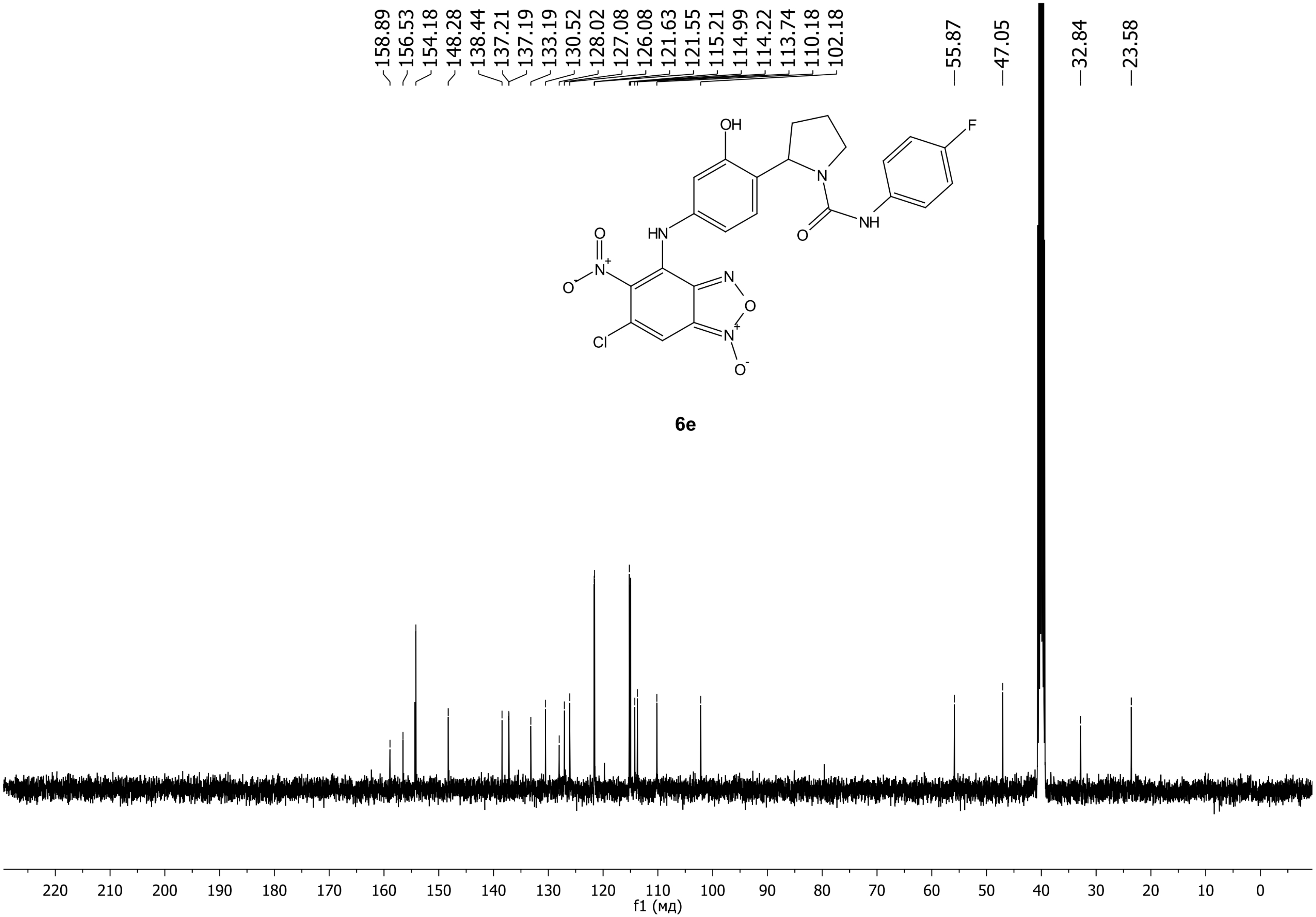
158.89
156.53
154.18
148.28
138.44
137.21
137.19
133.19
130.52
128.02
127.08
126.08
121.63
121.55
115.21
114.99
114.22
113.74
110.18
102.18
55.87
47.05
32.84
23.58
OH
F
N
HN
NH
O
N+
O-
N
O
N+
O-
Cl
6e
220 210 200 190 180 170 160 150 140 130 120 110 100 90 80 70 60 50 40 30 20 10 0
f1 (мд)

Figure S 46.

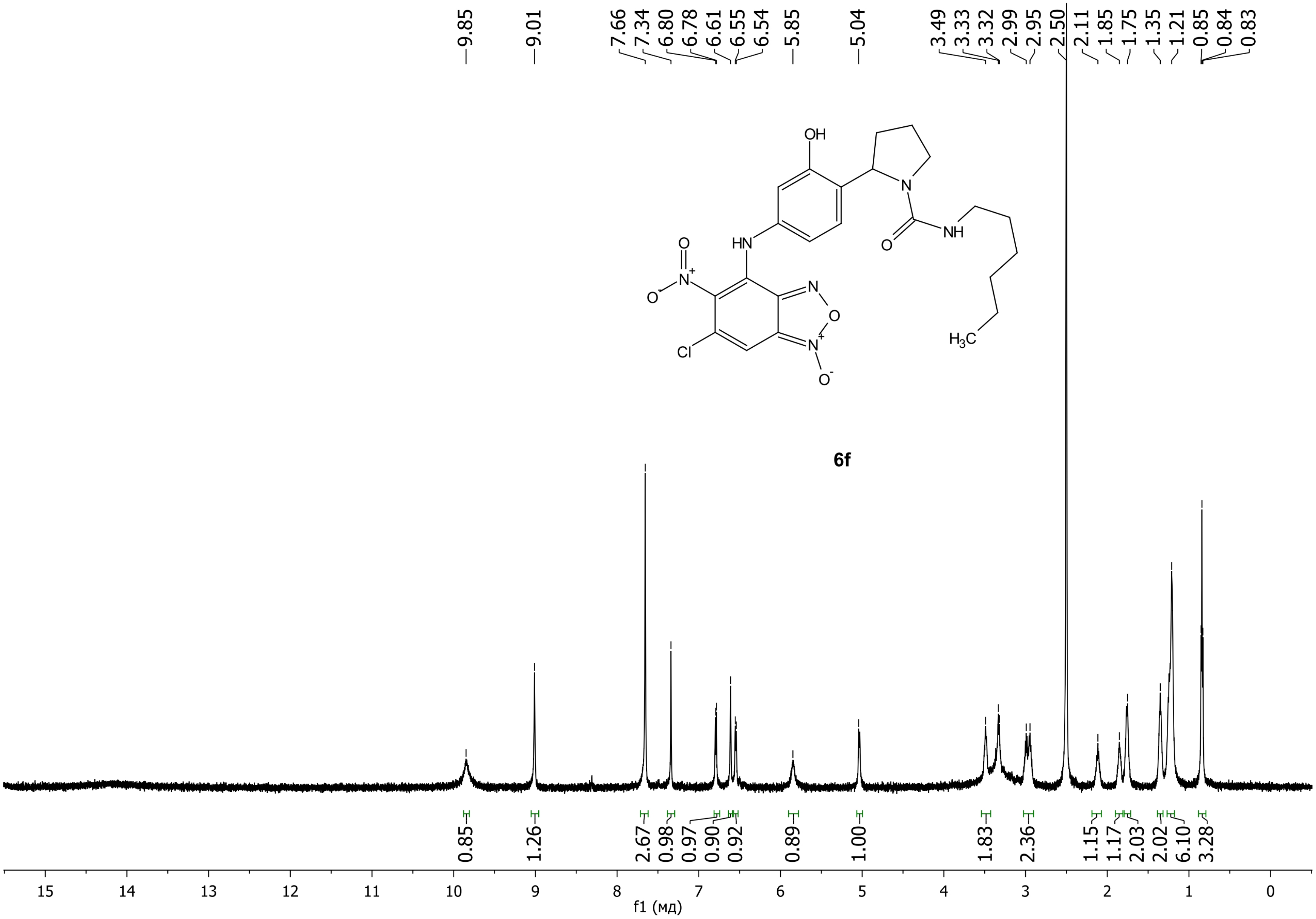
9.85
9.01
7.66
7.34
6.80
6.78
6.61
6.55
6.54
5.85
5.04
3.49
3.33
3.32
2.99
2.95
2.50
2.11
1.85
1.75
1.35
1.21
0.85
0.84
0.83
OH
HN
N
O
NH
O
N
O
O
N
O
N
O
Cl
H_3C
6f
0.85
1.26
2.67
0.98
0.97
0.90
0.92
0.89
1.00
1.83
2.36
1.15
1.17
2.03
2.02
6.10
3.28
15
14
13
12
11
10
9
8
7
6
5
4
3
2
1
0
f1 (мд)

Figure S 47.

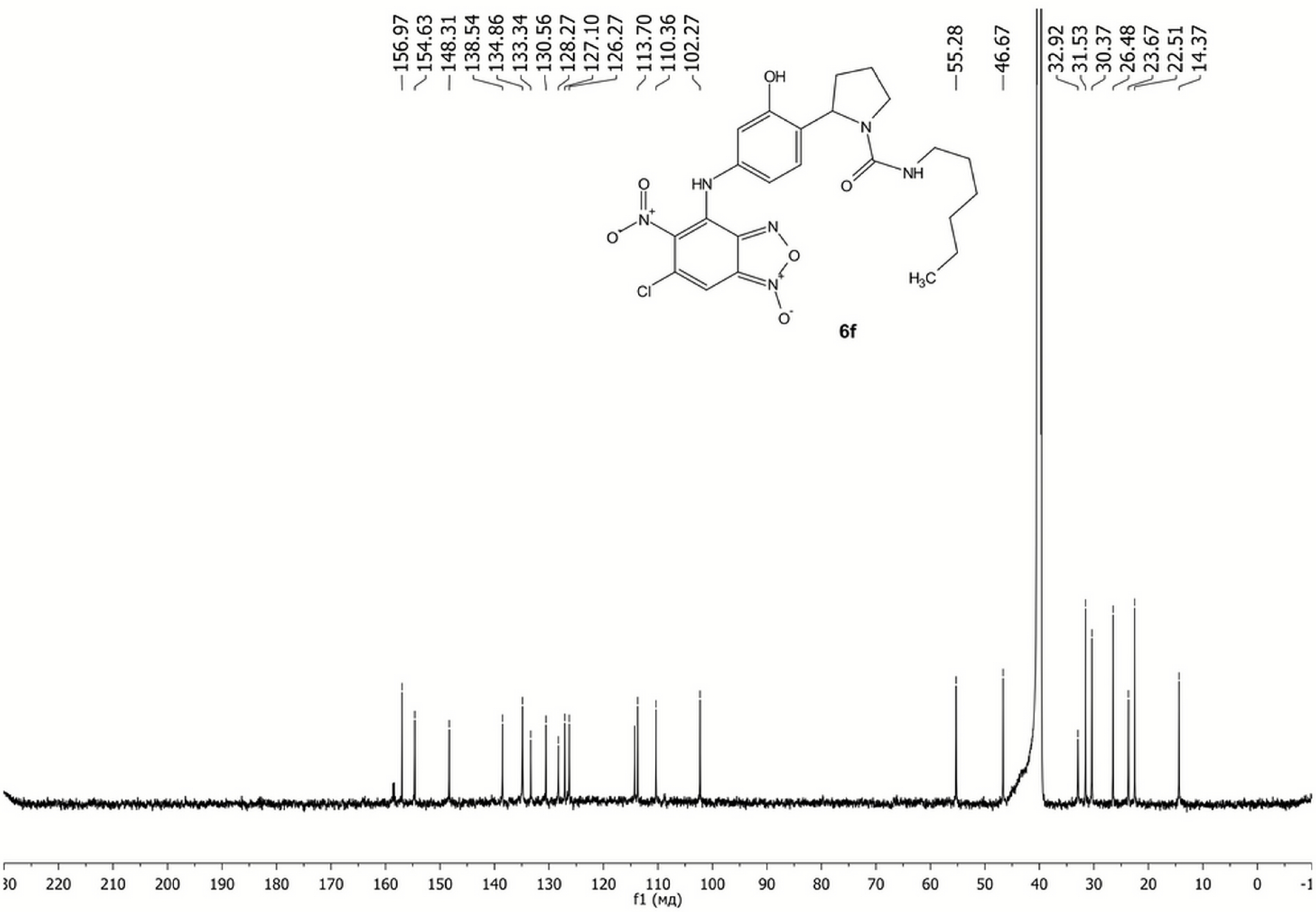
156.97
154.63
148.31
138.54
134.86
133.34
130.56
128.27
127.10
126.27
113.70
110.36
102.27
55.28
46.67
32.92
31.53
30.37
26.48
23.67
22.51
14.37
OH
N
O
NH
HN
O
N+
O-
N
O
N+
O-
Cl
H3C
6f
f1 (мд)

Figure S 48.

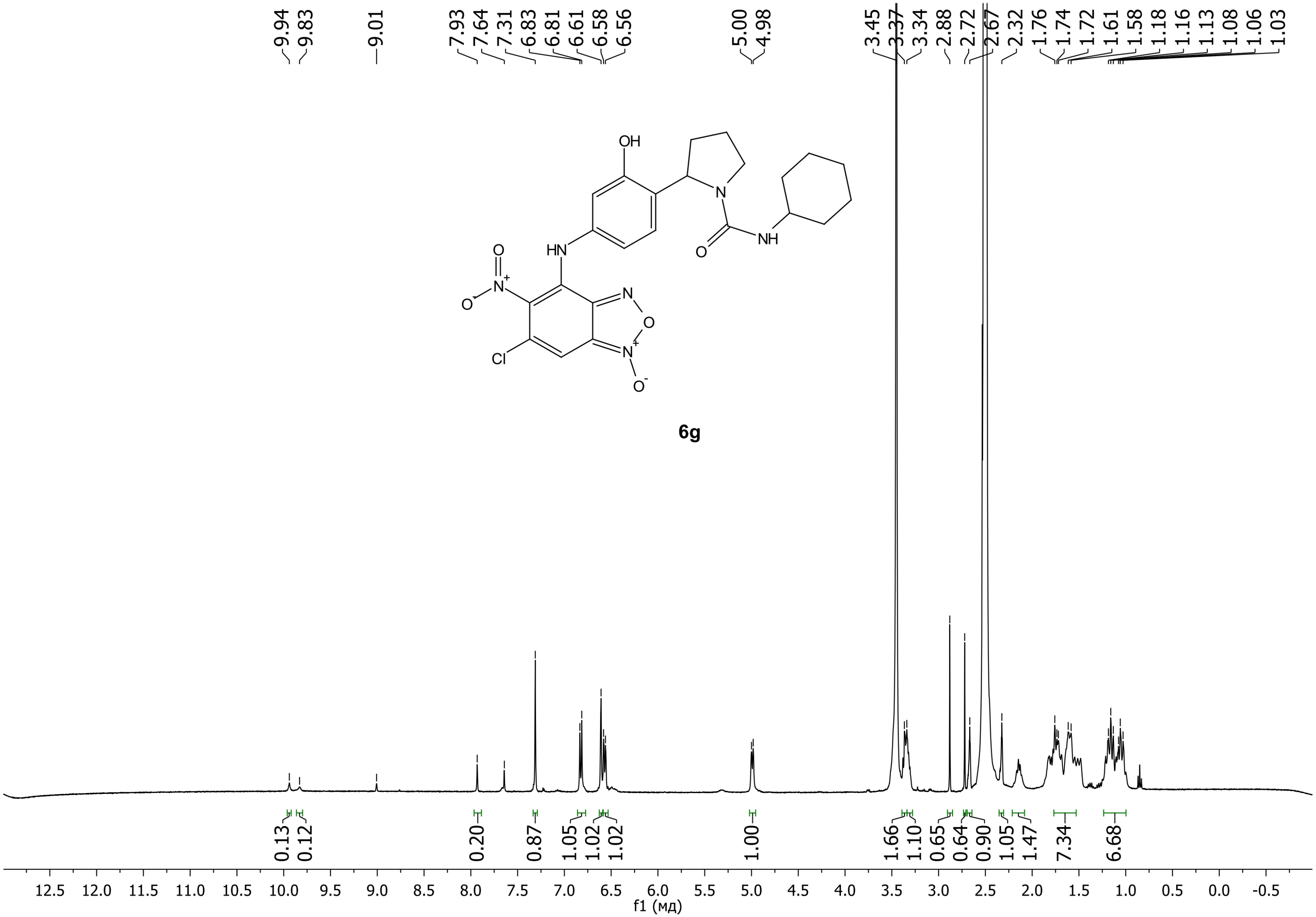
9.94 9.83 9.01 7.93 7.64 7.31 6.83 6.81 6.61 6.58 6.56 5.00 4.98 3.45 3.37 3.34 2.88 2.72 2.67 2.32 1.76 1.74 1.72 1.61 1.58 1.18 1.16 1.13 1.08 1.06 1.03
OH
HN
NH
N
O
Cl
6g
0.13 0.12 0.20 0.87 1.05 1.02 1.02 1.00 1.66 1.10 0.65 0.64 0.90 1.05 1.47 7.34 6.68
12.5 12.0 11.5 11.0 10.5 10.0 9.5 9.0 8.5 8.0 7.5 7.0 6.5 6.0 5.5 5.0 4.5 4.0 3.5 3.0 2.5 2.0 1.5 1.0 0.5 0.0 -0.5
f1 (мд)

Figure S 49.

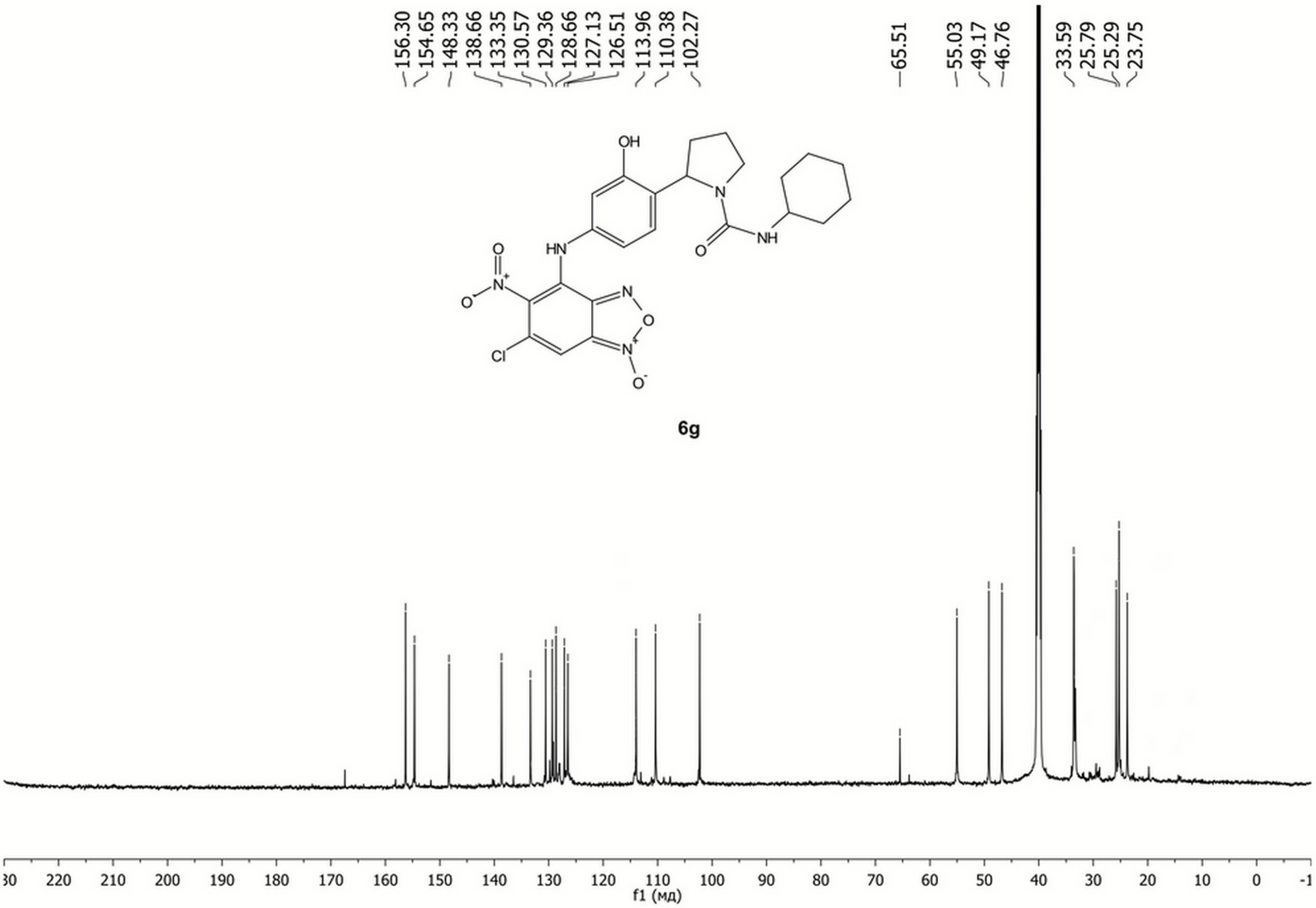
156.30
154.65
148.33
138.66
133.35
130.57
129.36
128.66
127.13
126.51
113.96
110.38
102.27
65.51
55.03
49.17
46.76
33.59
25.79
25.29
23.75
OH
HN
NH
N
O
Cl
6g
f1 (мд)

Figure S 50.

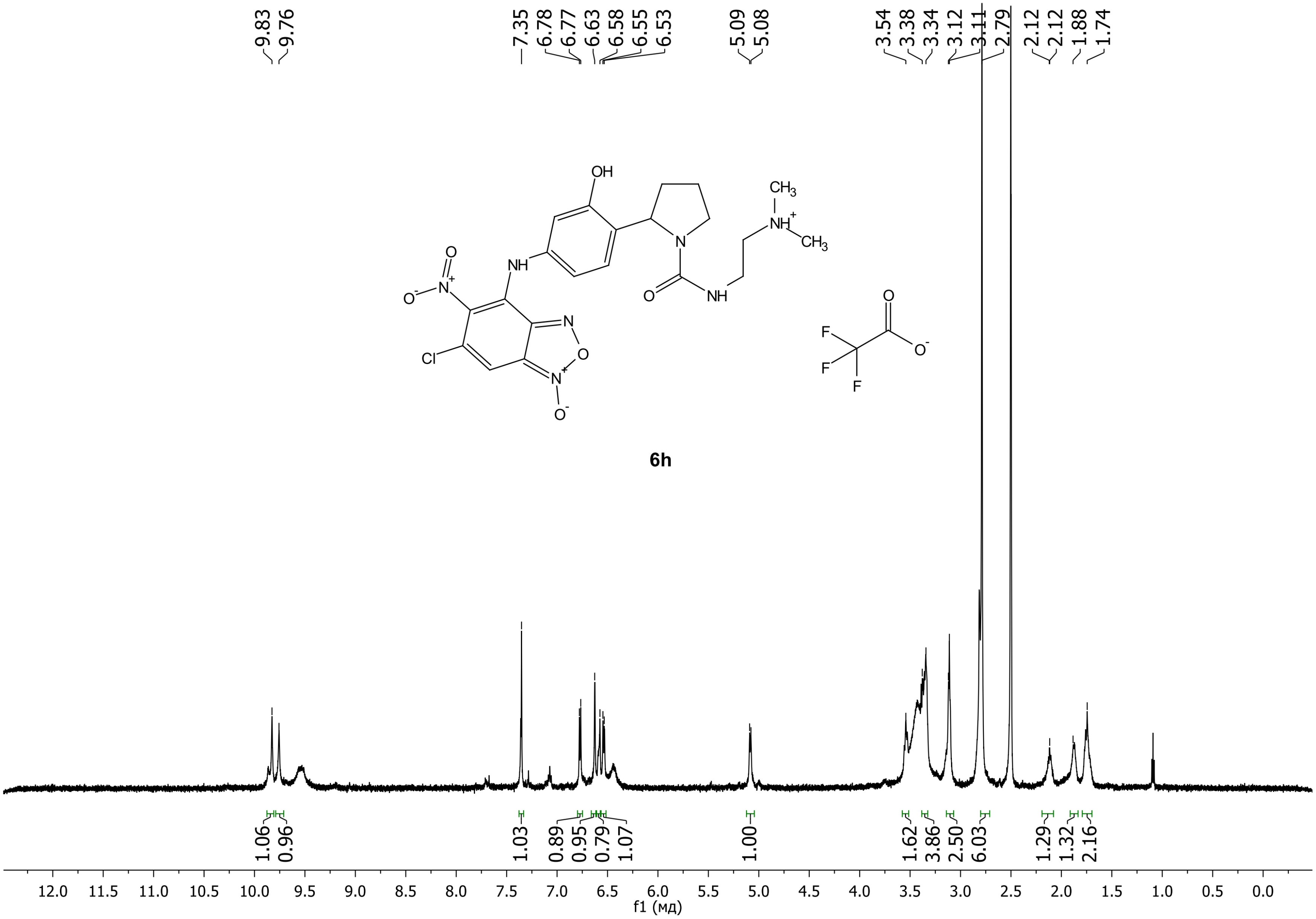
9.83
9.76
7.35
6.78
6.77
6.63
6.58
6.55
6.53
5.09
5.08
3.54
3.38
3.34
3.12
3.11
2.79
2.12
2.12
1.88
1.74
OH
NH
O
N+
O-
N
O
N+
O-
Cl
N
O
NH
NH+
CH3
CH3
O
F
F
F
O-
6h
1.06
0.96
1.03
0.89
0.95
0.79
1.07
1.00
1.62
3.86
2.50
6.03
1.29
1.32
2.16
12.0
11.5
11.0
10.5
10.0
9.5
9.0
8.5
8.0
7.5
7.0
6.5
6.0
5.5
5.0
4.5
4.0
3.5
3.0
2.5
2.0
1.5
1.0
0.5
0.0
f1 (мд)

Figure S 51.

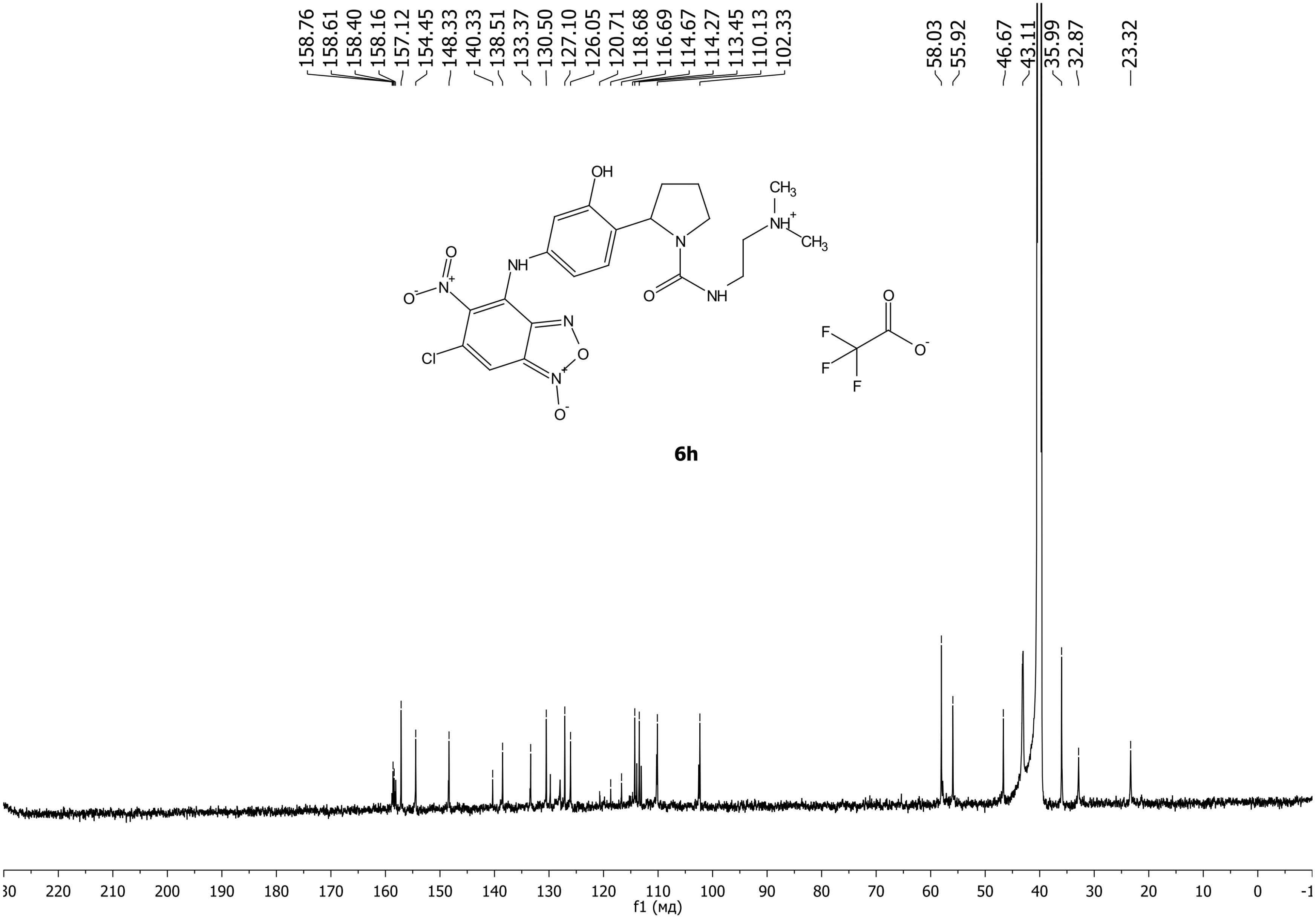

6h

Figure S 52.

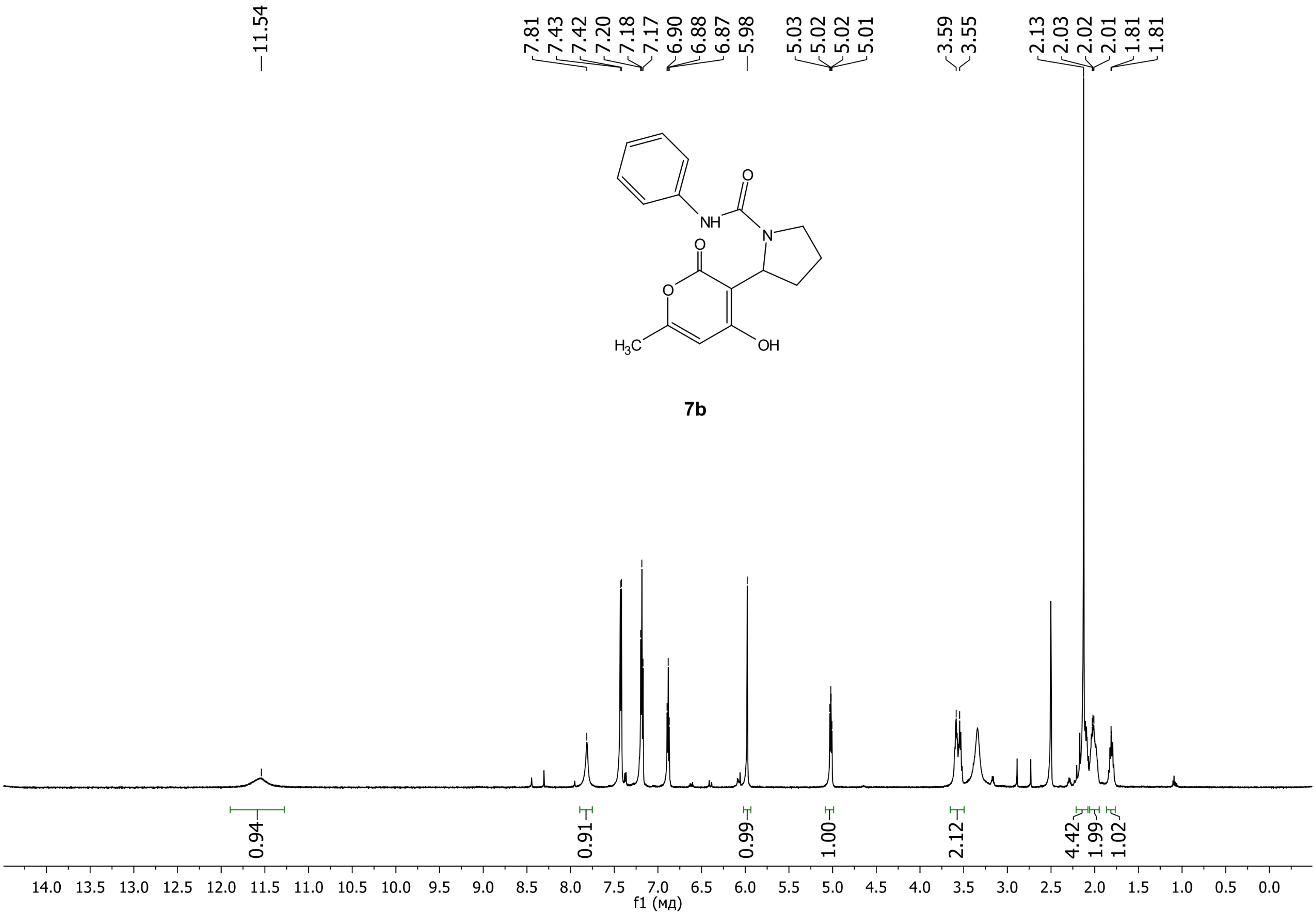

7b

Figure S 53.

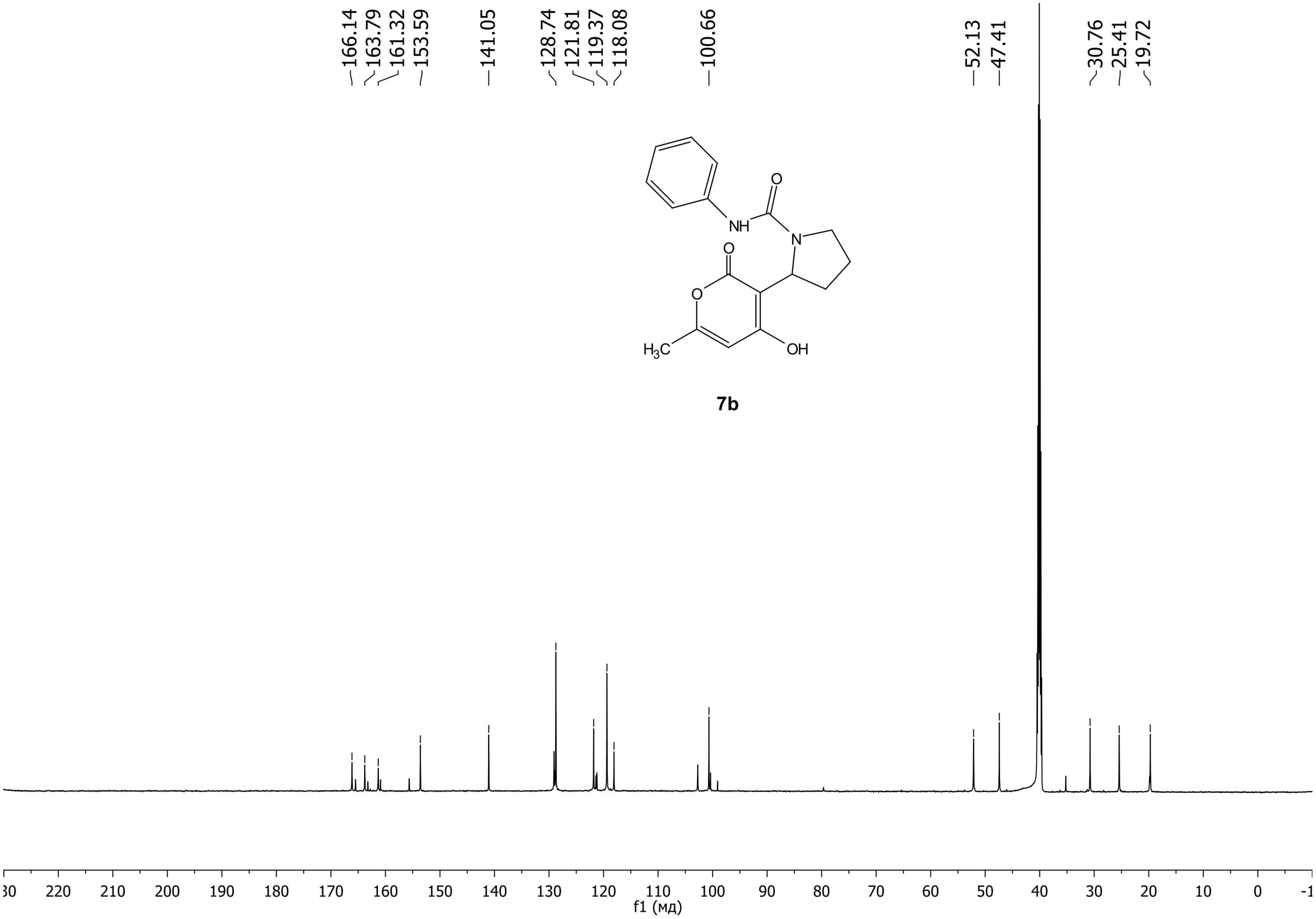
166.14
163.79
161.32
153.59
141.05
128.74
121.81
119.37
118.08
100.66
52.13
47.41
30.76
25.41
19.72
O
NH
N
O
O
H3C
OH
7b
220
210
200
190
180
170
160
150
140
130
120
110
100
90
80
70
60
50
40
30
20
10
0
f1 (мд)

Figure S 54.

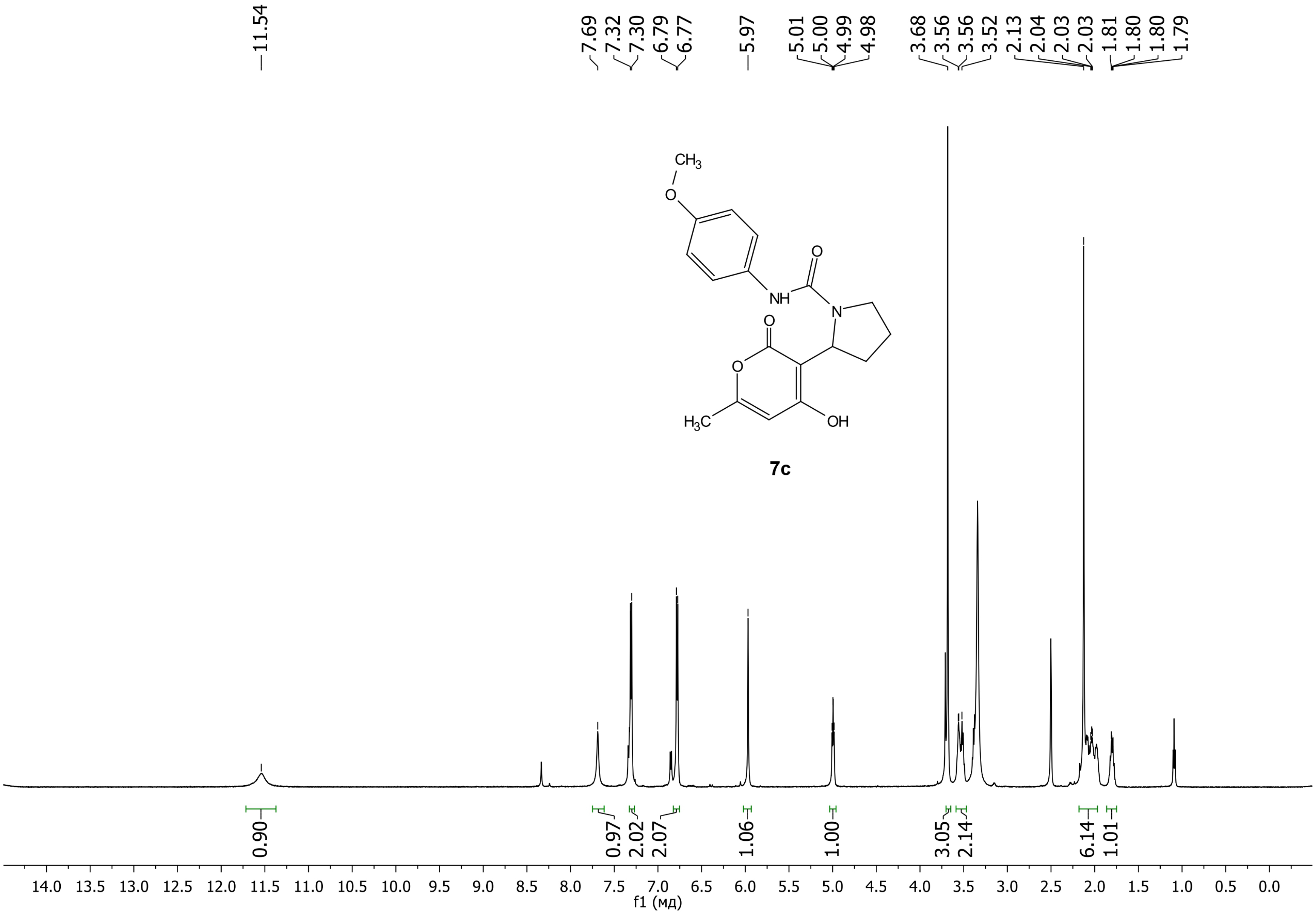

11.54
7.69
7.32
7.30
6.79
6.77
5.97
5.01
5.00
4.99
4.98
3.68
3.56
3.56
3.52
2.13
2.04
2.03
2.03
1.81
1.80
1.80
1.79
CH3
O
O
NH
N
O
O
H3C
OH
7c
0.90
0.97
2.02
2.07
1.06
1.00
3.05
2.14
6.14
1.01
14.0 13.5 13.0 12.5 12.0 11.5 11.0 10.5 10.0 9.5 9.0 8.5 8.0 7.5 7.0 6.5 6.0 5.5 5.0 4.5 4.0 3.5 3.0 2.5 2.0 1.5 1.0 0.5 0.0
f1 (мд)

Figure S 55.

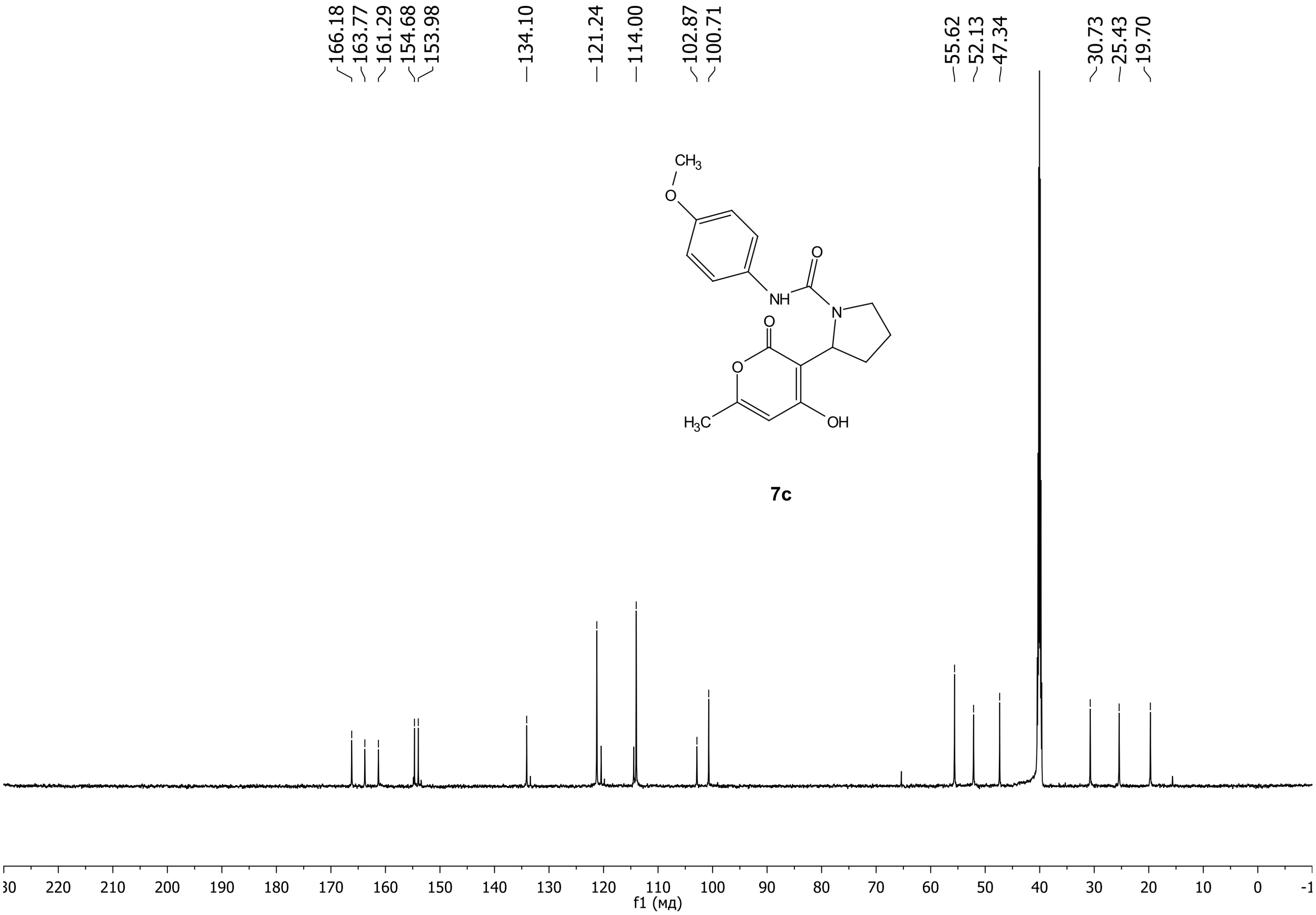
166.18
163.77
161.29
154.68
153.98
134.10
121.24
114.00
102.87
100.71
55.62
52.13
47.34
30.73
25.43
19.70
CH3
O
O
NH
N
O
O
H3C
OH
7c
f1 (мд)

Figure S 56.

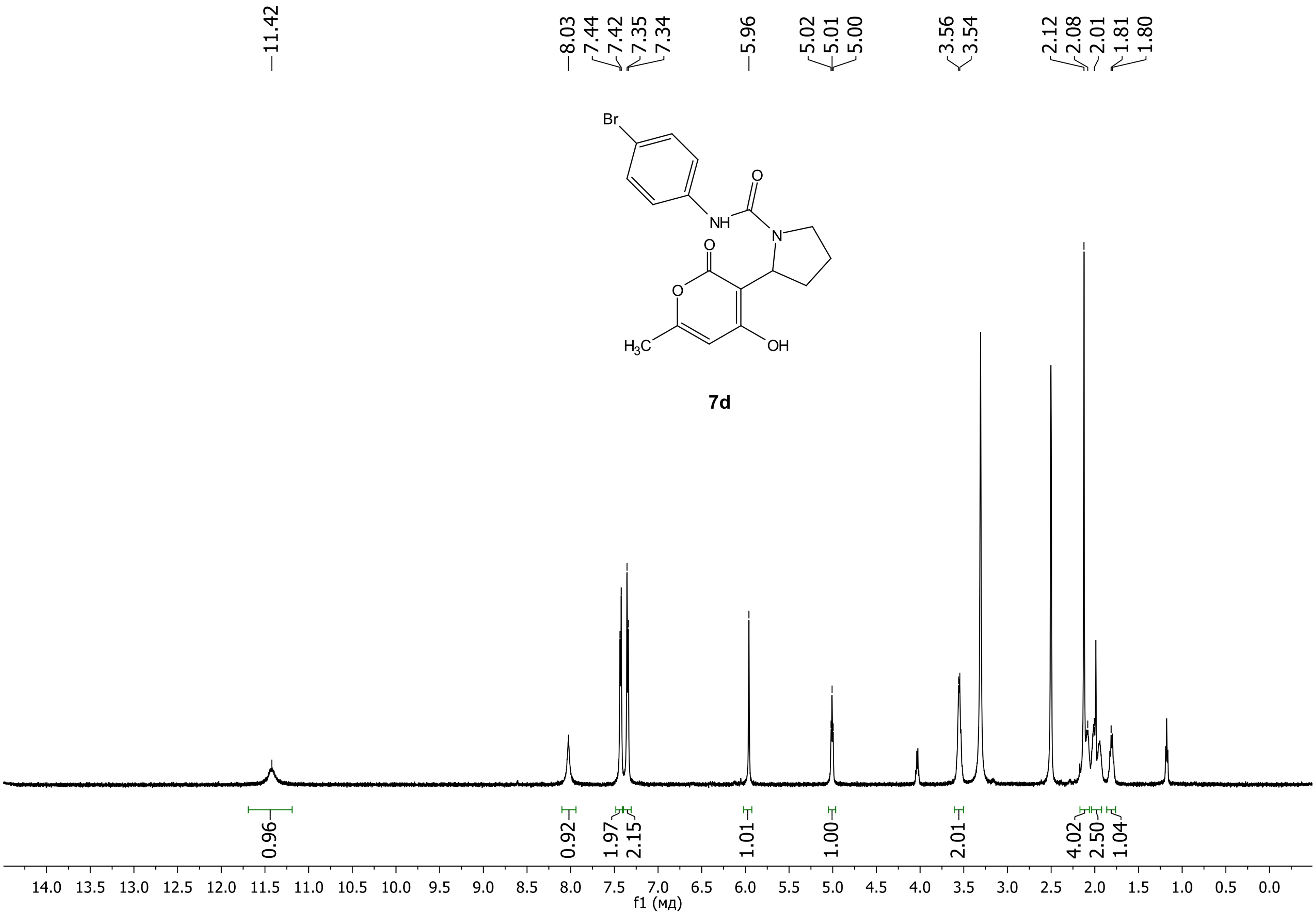

11.42
8.03
7.44
7.42
7.35
7.34
5.96
5.02
5.01
5.00
3.56
3.54
2.12
2.08
2.01
1.81
1.80
Br
O
NH
N
O
O
H3C
OH
7d
0.96
0.92
1.97
2.15
1.01
1.00
2.01
4.02
2.50
1.04
14.0
13.5
13.0
12.5
12.0
11.5
11.0
10.5
10.0
9.5
9.0
8.5
8.0
7.5
7.0
6.5
6.0
5.5
5.0
4.5
4.0
3.5
3.0
2.5
2.0
1.5
1.0
0.5
0.0
f1 (мд)

Figure S 57.

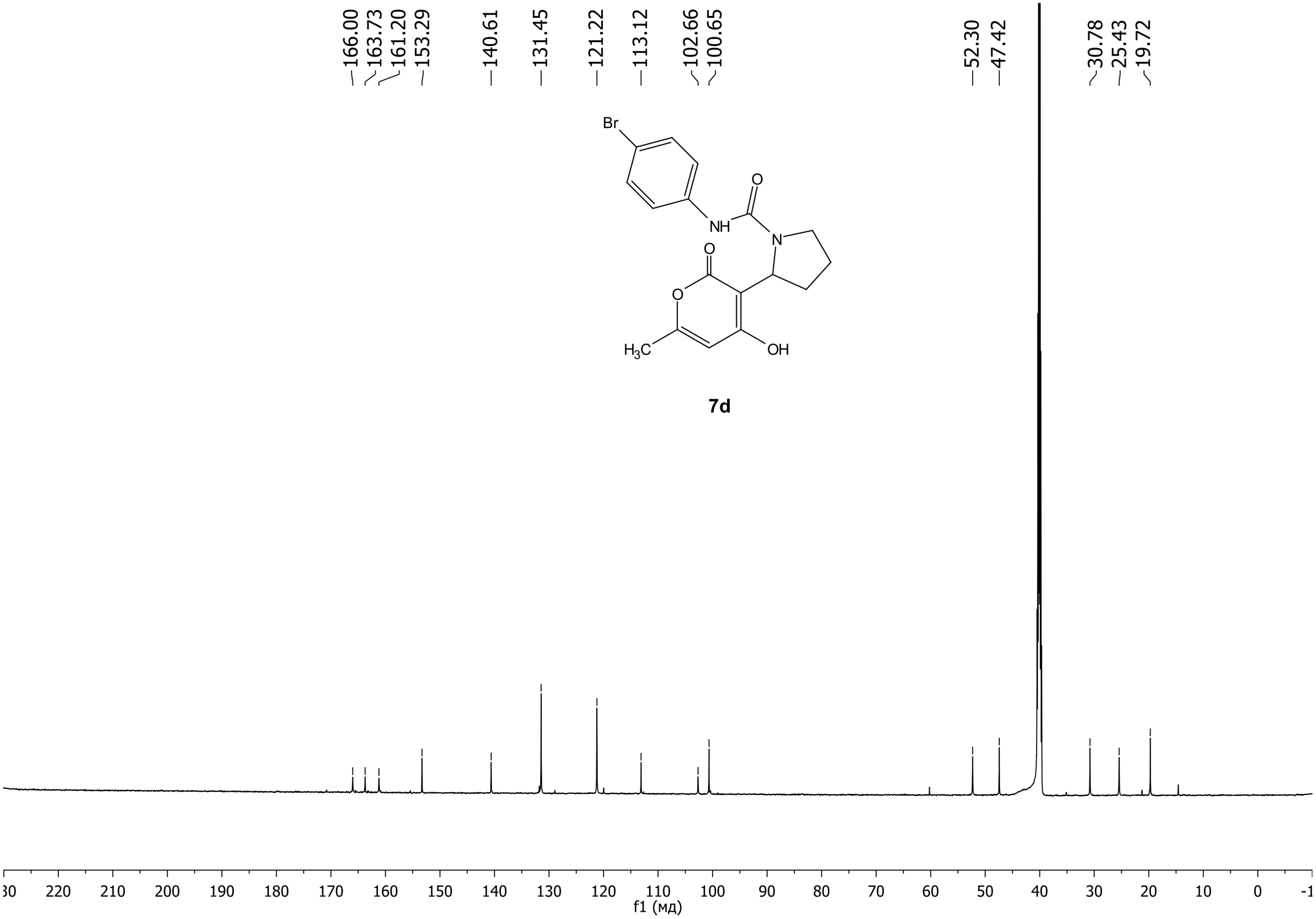
166.00
163.73
161.20
153.29
140.61
131.45
121.22
113.12
102.66
100.65
52.30
47.42
30.78
25.43
19.72
Br
O
NH
N
O
O
H3C
OH
7d
f1 (мд)

Figure S 58.

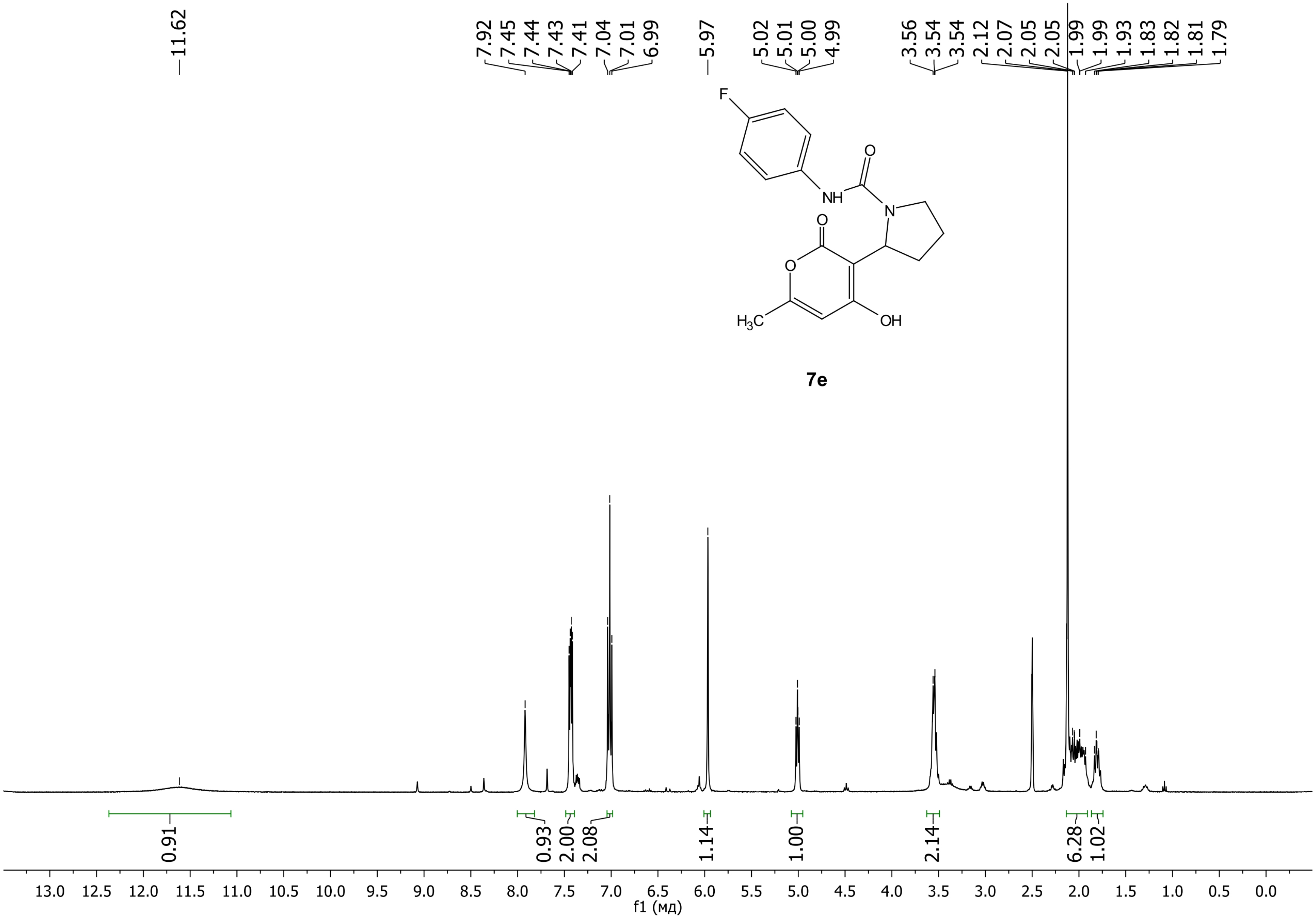

11.62
7.92
7.45
7.44
7.43
7.41
7.04
7.01
6.99
5.97
5.02
5.01
5.00
4.99
3.56
3.54
3.54
2.12
2.07
2.05
2.05
1.99
1.99
1.93
1.83
1.82
1.81
1.79
7e
0.91
0.93
2.00
2.08
1.14
1.00
2.14
6.28
1.02
f1 (мд)

Figure S 59.

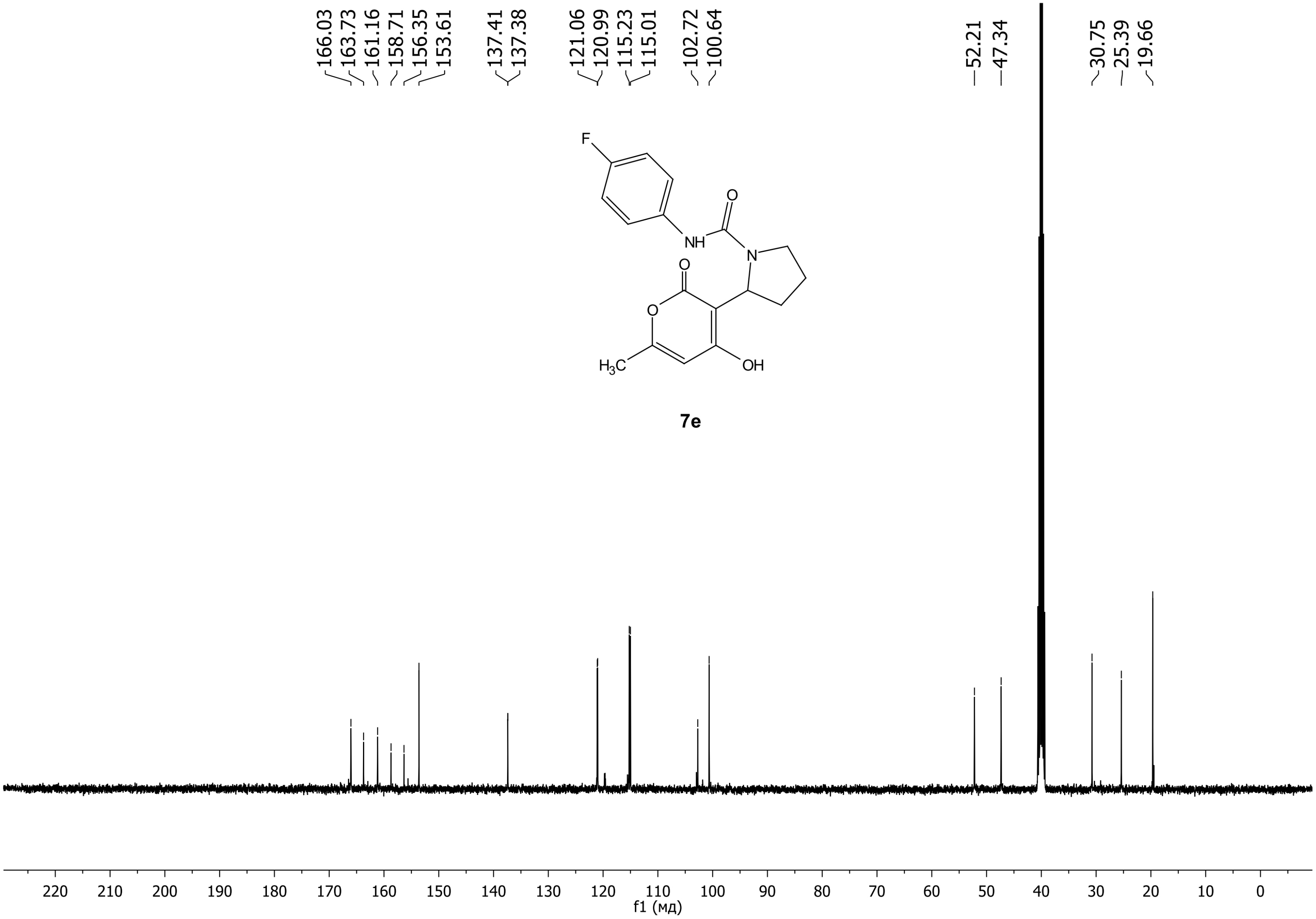
166.03
163.73
161.16
158.71
156.35
153.61
137.41
137.38
121.06
120.99
115.23
115.01
102.72
100.64
52.21
47.34
30.75
25.39
19.66
F
O
NH
N
O
O
H3C
OH
7e
220 210 200 190 180 170 160 150 140 130 120 110 100 90 80 70 60 50 40 30 20 10 0
f1 (мд)

Figure S 60.

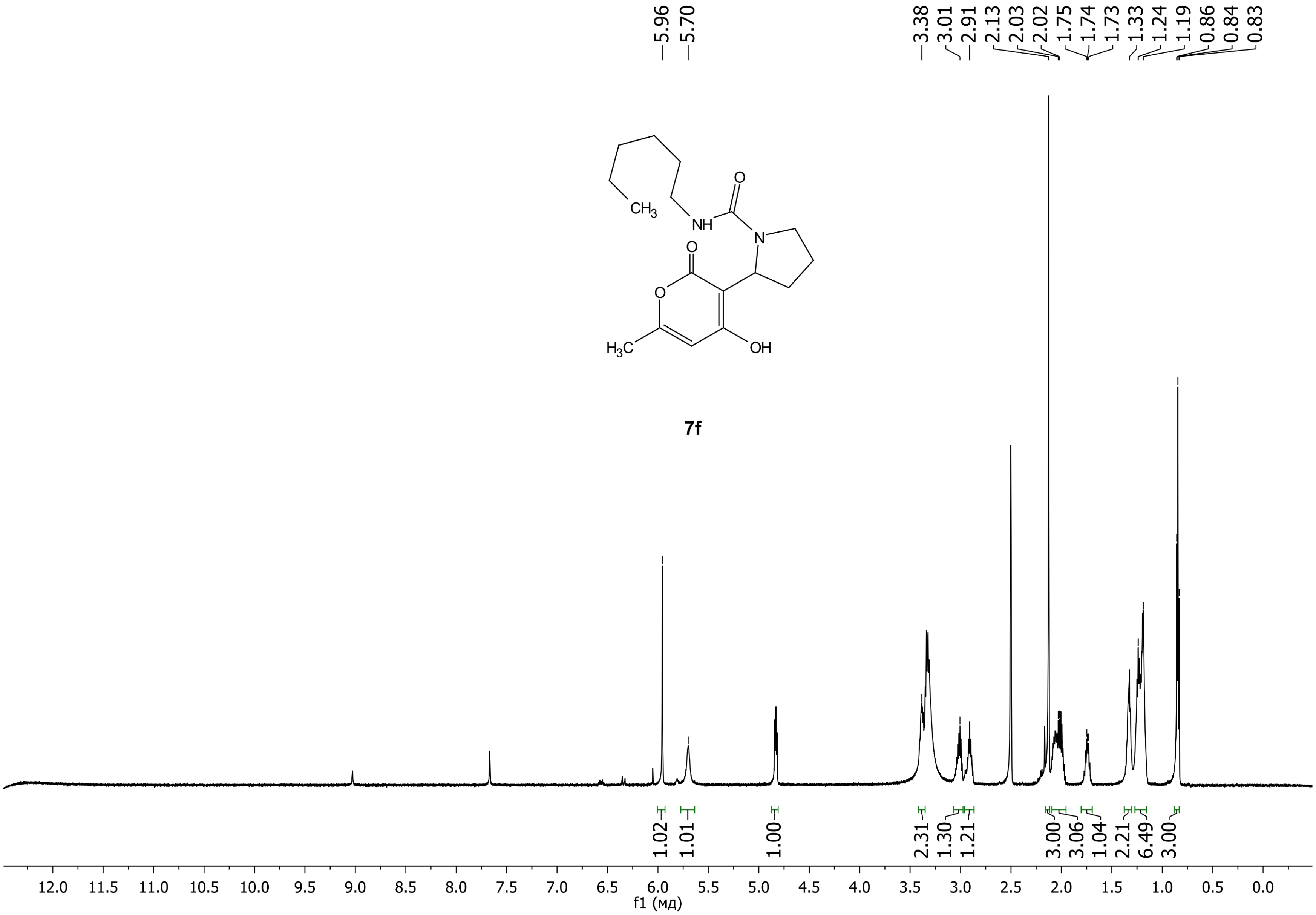
5.96
5.70
3.38
3.01
2.91
2.13
2.03
2.02
1.75
1.74
1.73
1.33
1.24
1.19
0.86
0.84
0.83
CH3
NH
O
N
O
O
H3C
OH
7f
1.02
1.01
1.00
2.31
1.30
1.21
3.00
3.06
1.04
2.21
6.49
3.00
f1 (мд)

Figure S 61.

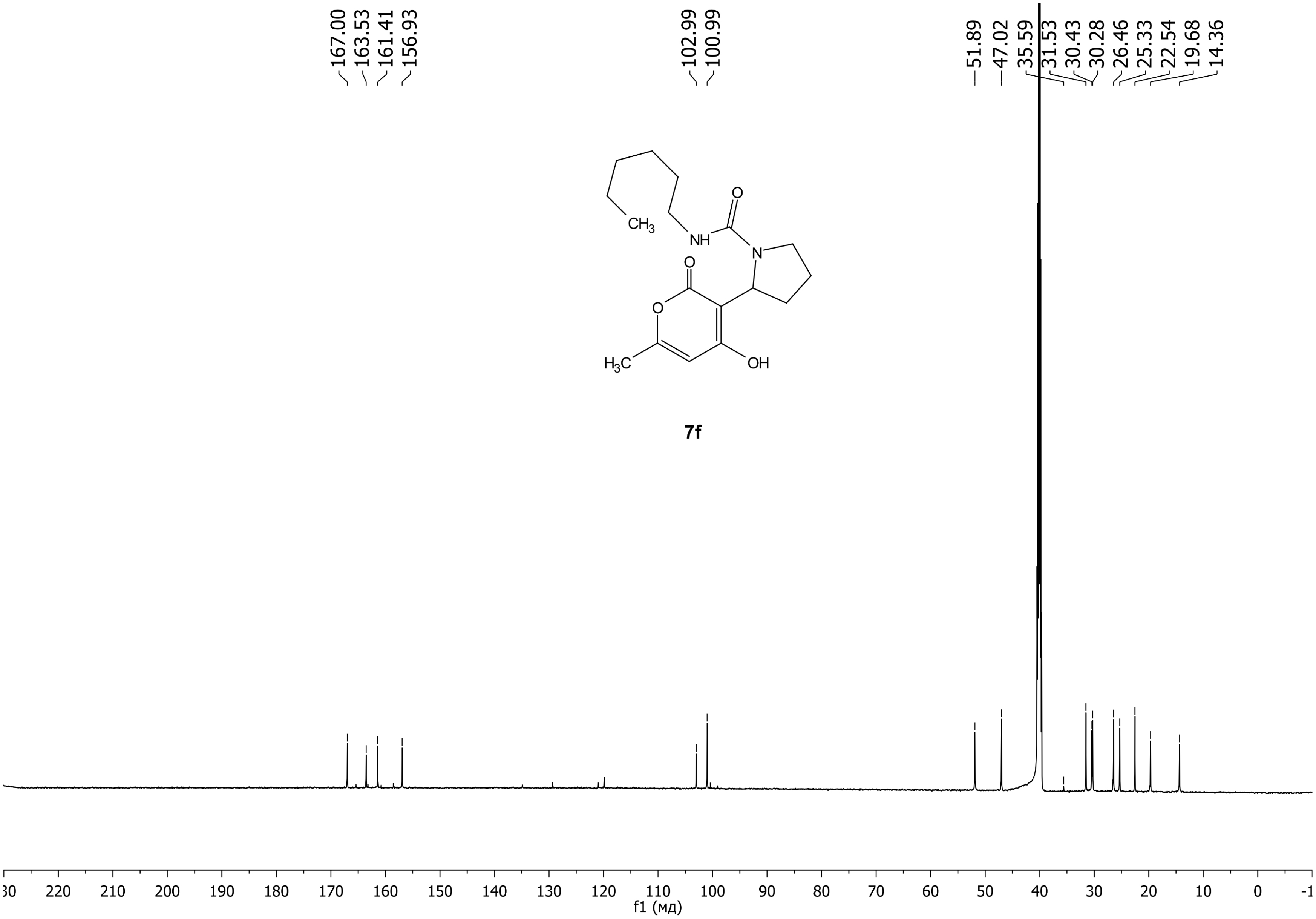
167.00
163.53
161.41
156.93
102.99
100.99
51.89
47.02
35.59
31.53
30.43
30.28
26.46
25.33
22.54
19.68
14.36
CH_3
NH
O
N
O
O
H_3C
OH
7f
f1 (мд)

Figure S 62.

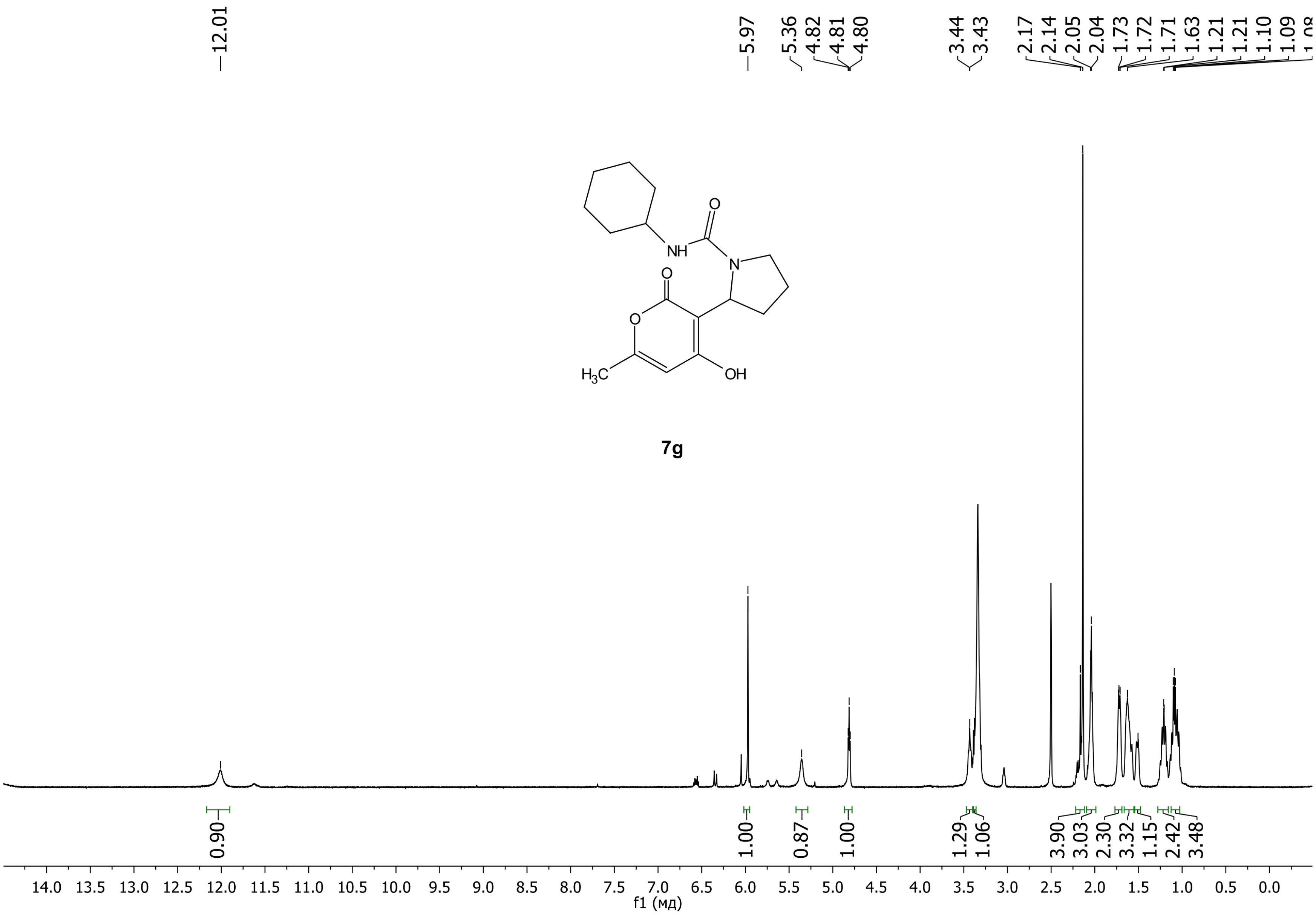
12.01
5.97
5.36
4.82
4.81
4.80
3.44
3.43
2.17
2.14
2.05
2.04
1.73
1.72
1.71
1.63
1.21
1.21
1.10
1.09
O
NH
N
O
O
H$_3$C
OH
7g
0.90
1.00
0.87
1.00
1.29
1.06
3.90
3.03
2.30
3.32
1.15
2.42
3.48
14.0 13.5 13.0 12.5 12.0 11.5 11.0 10.5 10.0 9.5 9.0 8.5 8.0 7.5 7.0 6.5 6.0 5.5 5.0 4.5 4.0 3.5 3.0 2.5 2.0 1.5 1.0 0.5 0.0
f1 (мд)

Figure S 63.

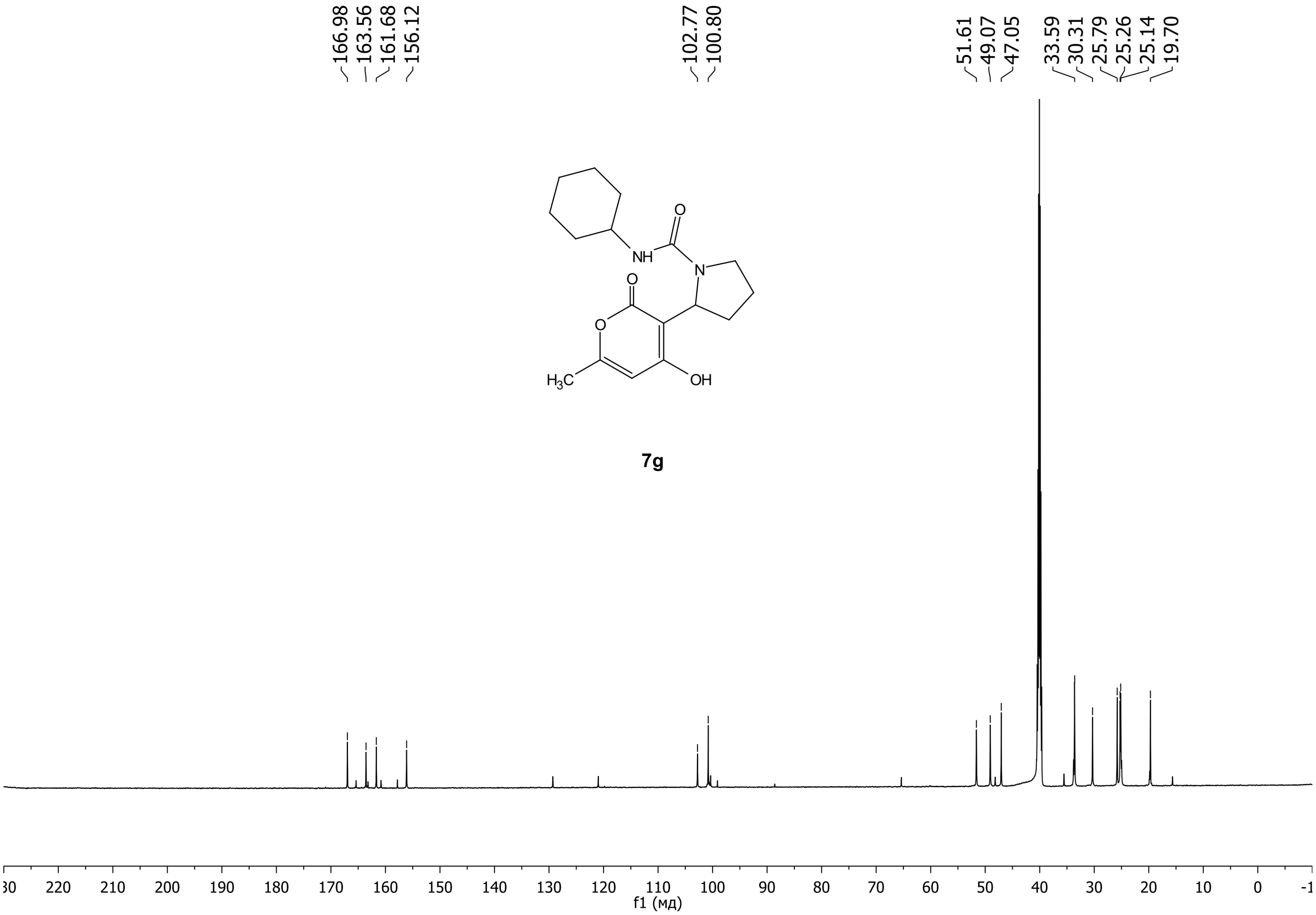
166.98
163.56
161.68
156.12
102.77
100.80
51.61
49.07
47.05
33.59
30.31
25.79
25.26
25.14
19.70
O
NH
N
O
O
H3C
OH
7g
f1 (мд)

Figure S 64.

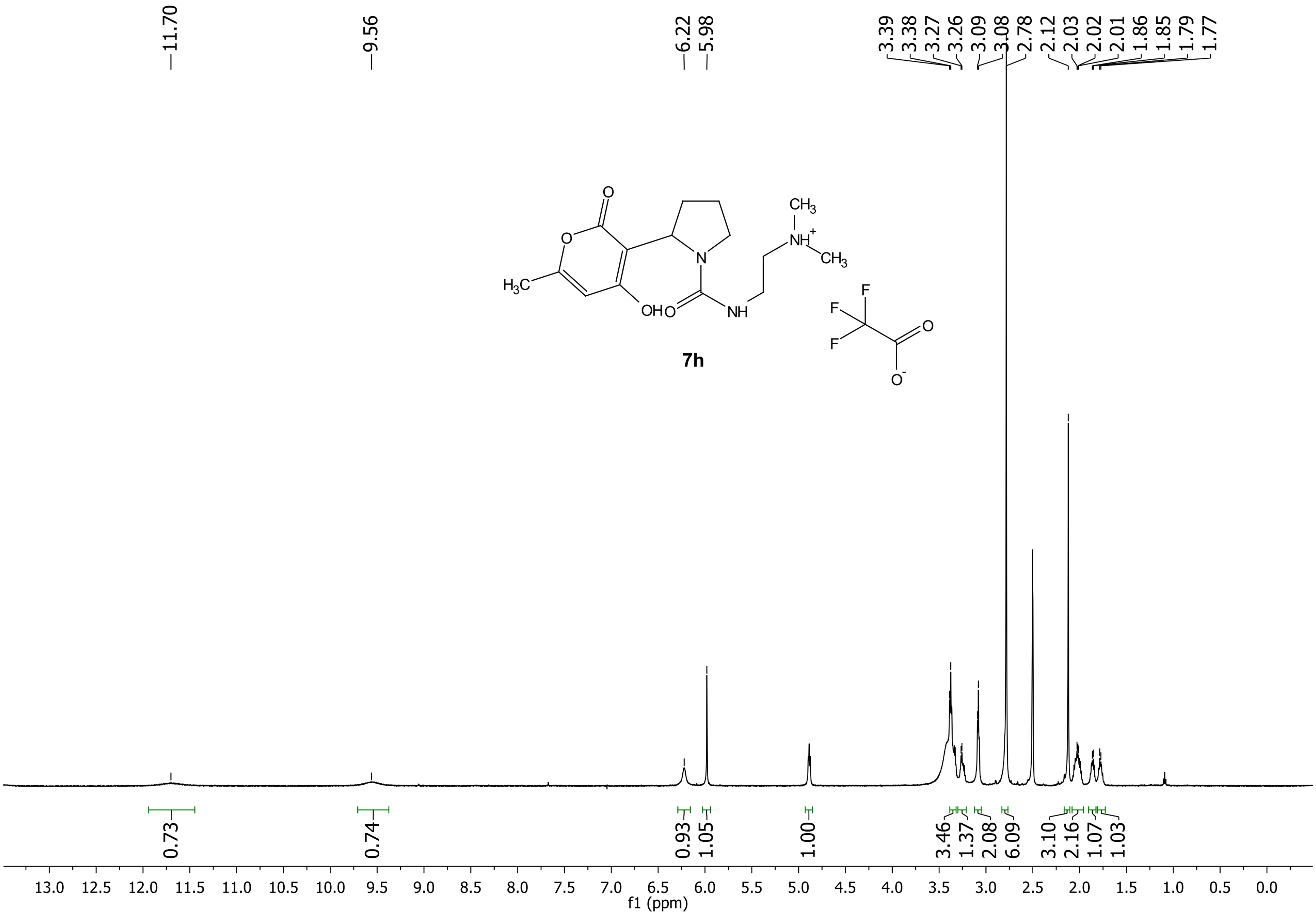

11.70
9.56
6.22
5.98
3.39
3.38
3.27
3.26
3.09
3.08
2.78
2.12
2.03
2.02
2.01
1.86
1.85
1.79
1.77
7h
0.73
0.74
0.93
1.05
1.00
3.46
1.37
2.08
6.09
3.10
2.16
1.07
1.03
f1 (ppm)

Figure S 65.

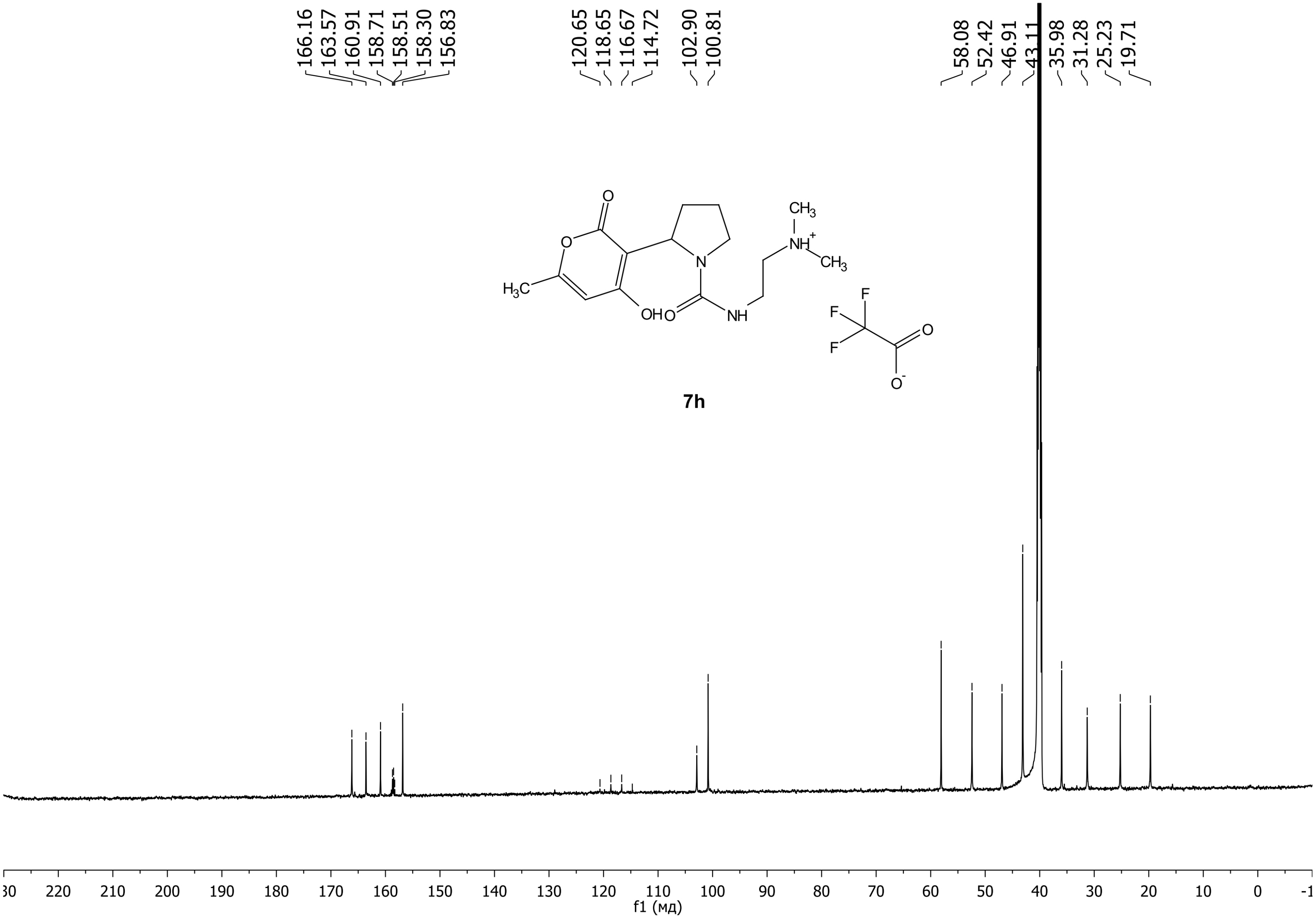

7h

Figure S 66.

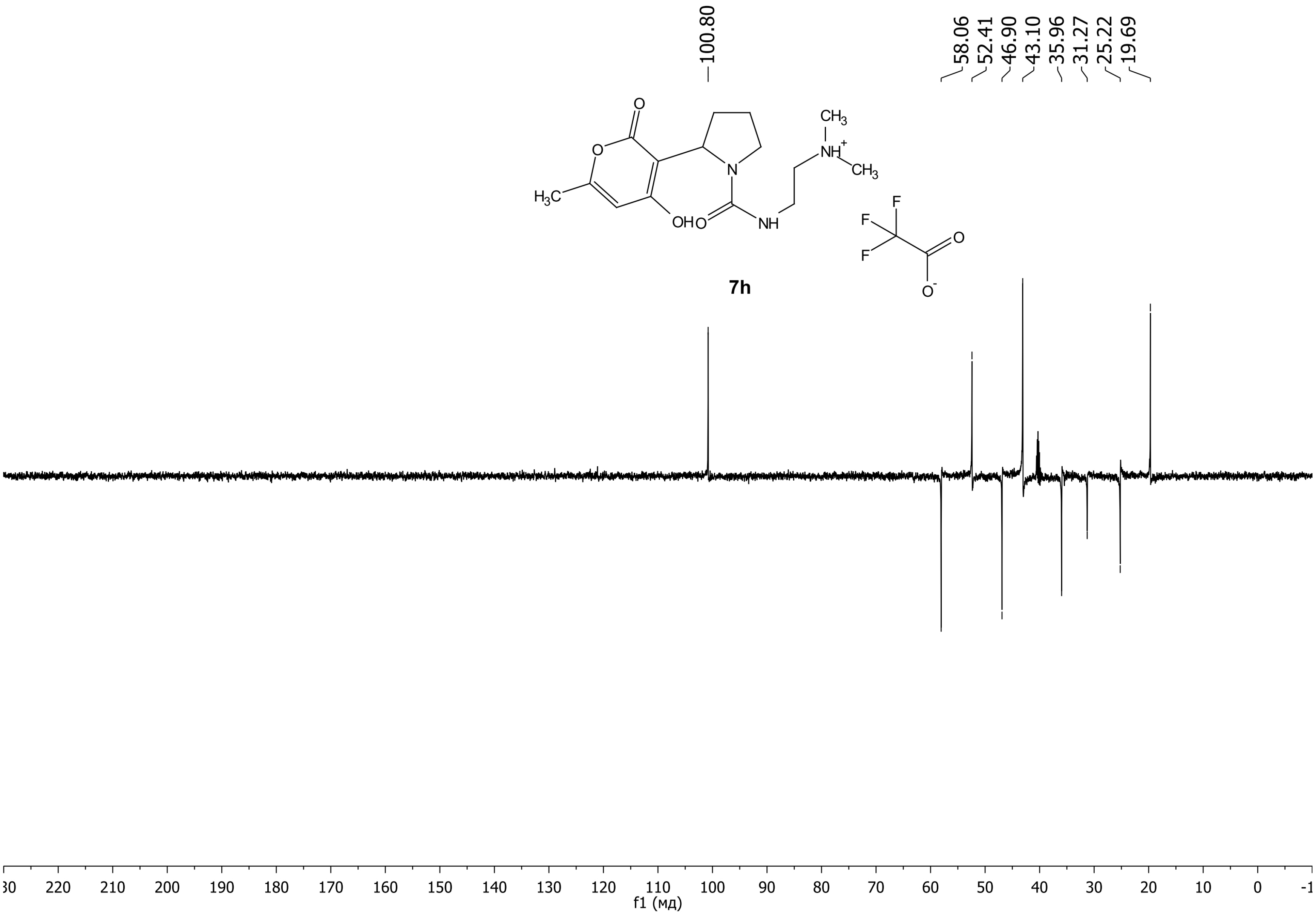

Figure S 67.

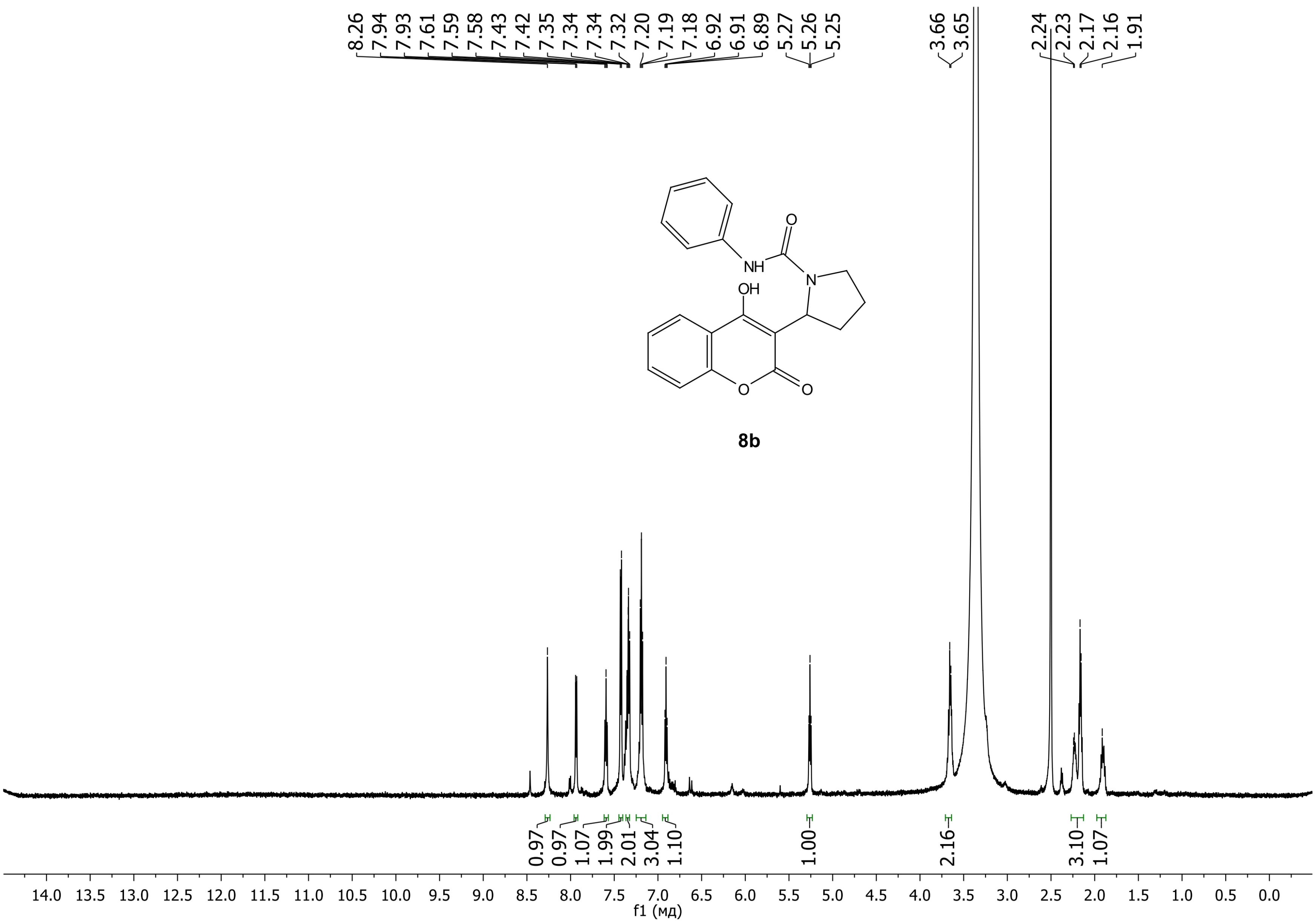
8.26
7.94
7.93
7.61
7.59
7.58
7.43
7.42
7.35
7.34
7.34
7.32
7.20
7.19
7.18
6.92
6.91
6.89
5.27
5.26
5.25
3.66
3.65
2.24
2.23
2.17
2.16
1.91
NH
O
N
OH
O
O
8b
0.97
0.97
1.07
1.99
2.01
3.04
1.10
1.00
2.16
3.10
1.07
14.0 13.5 13.0 12.5 12.0 11.5 11.0 10.5 10.0 9.5 9.0 8.5 8.0 7.5 7.0 6.5 6.0 5.5 5.0 4.5 4.0 3.5 3.0 2.5 2.0 1.5 1.0 0.5 0.0
f1 (мд)

Figure S 68.

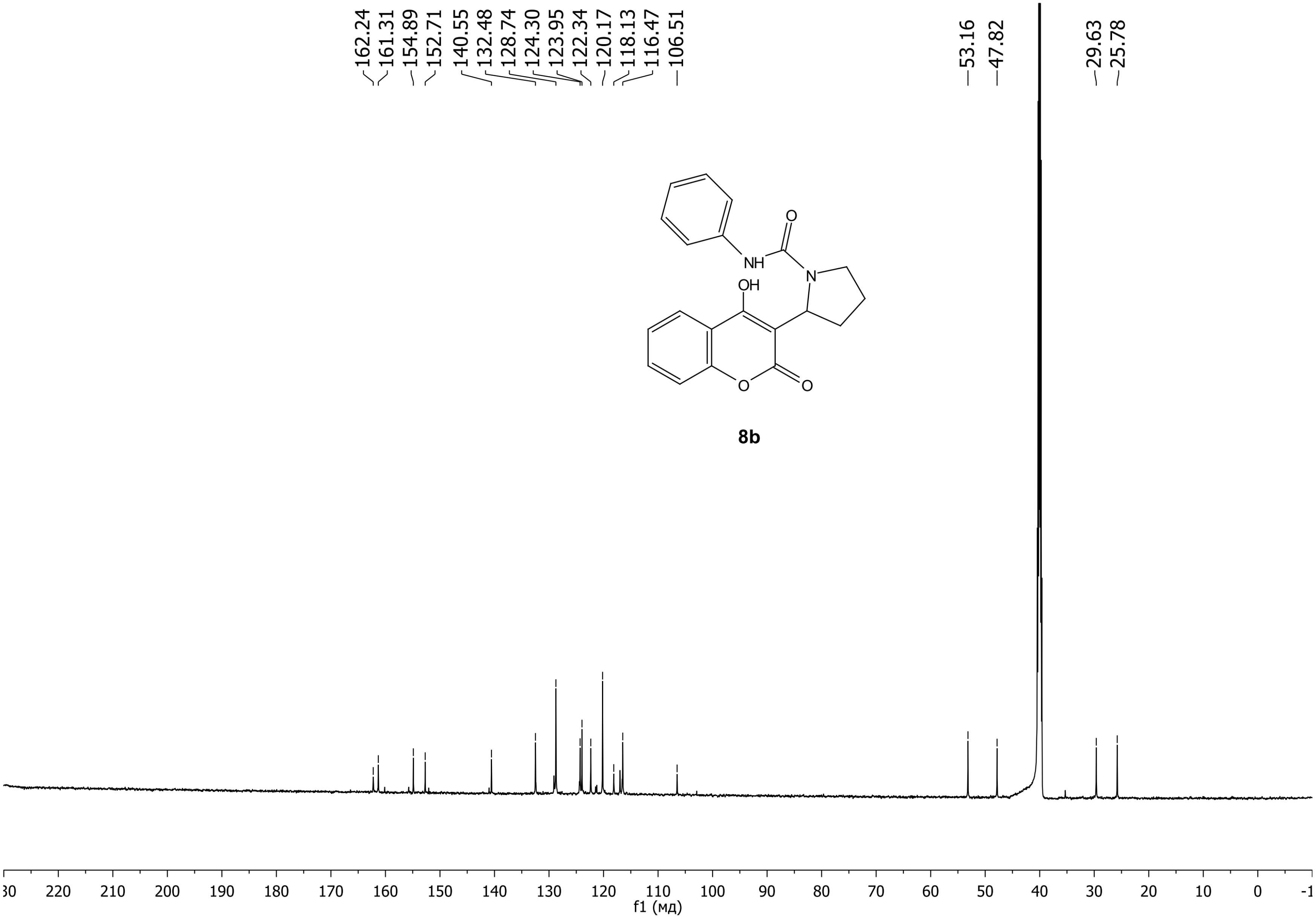
162.24
161.31
154.89
152.71
140.55
132.48
128.74
124.30
123.95
122.34
120.17
118.13
116.47
106.51
53.16
47.82
29.63
25.78
O
NH
N
OH
O
O
8b
30 220 210 200 190 180 170 160 150 140 130 120 110 100 90 80 70 60 50 40 30 20 10 0 -1
f1 (мд)

Figure S 69.

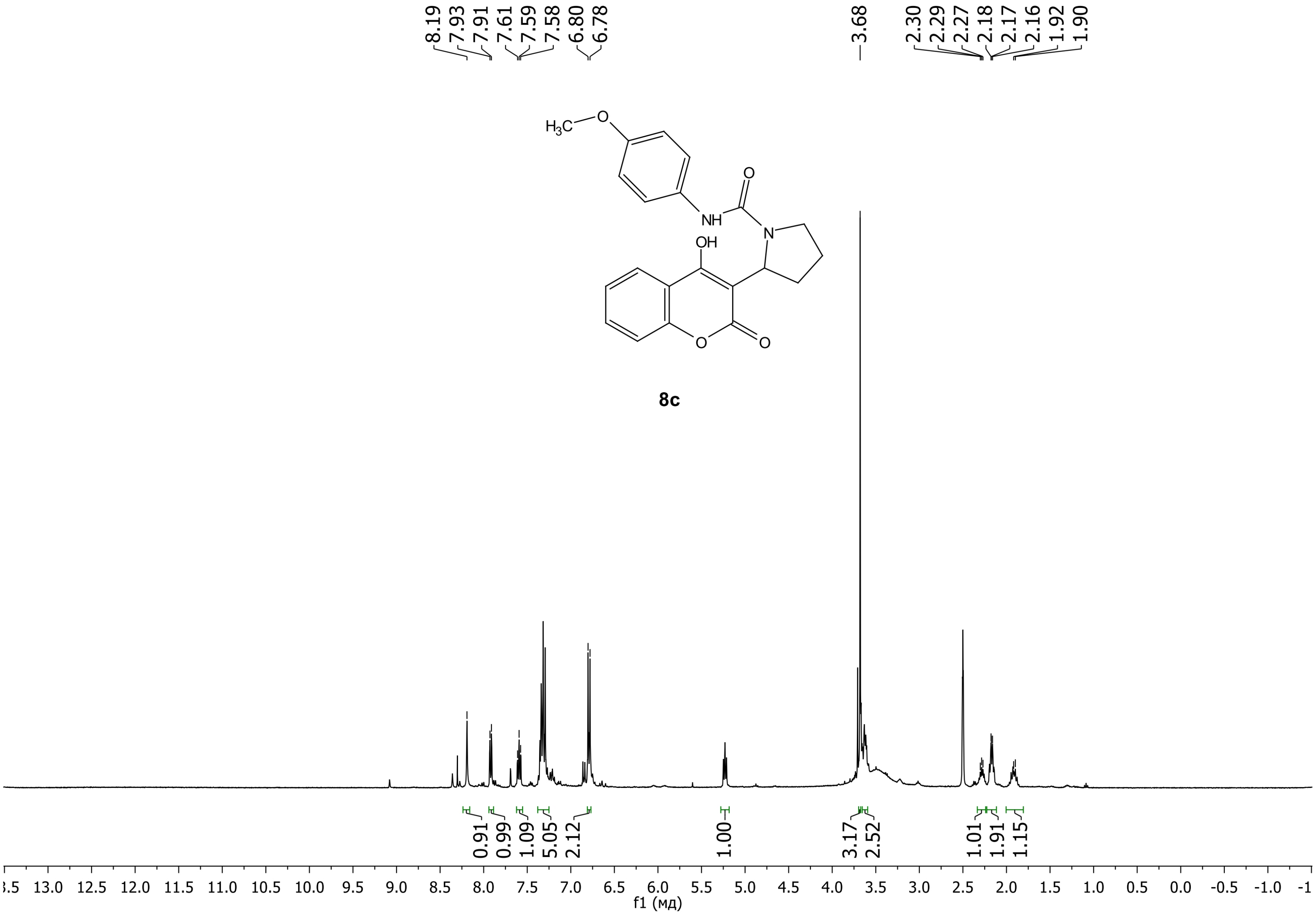
8.19
7.93
7.91
7.61
7.59
7.58
6.80
6.78
3.68
2.30
2.29
2.27
2.18
2.17
2.16
1.92
1.90
H3C
O
NH
O
N
OH
O
O
8c
0.91
0.99
1.09
5.05
2.12
1.00
3.17
2.52
1.01
1.91
1.15
13.0 12.5 12.0 11.5 11.0 10.5 10.0 9.5 9.0 8.5 8.0 7.5 7.0 6.5 6.0 5.5 5.0 4.5 4.0 3.5 3.0 2.5 2.0 1.5 1.0 0.5 0.0 -0.5 -1.0
f1 (мд)

Figure S 70.

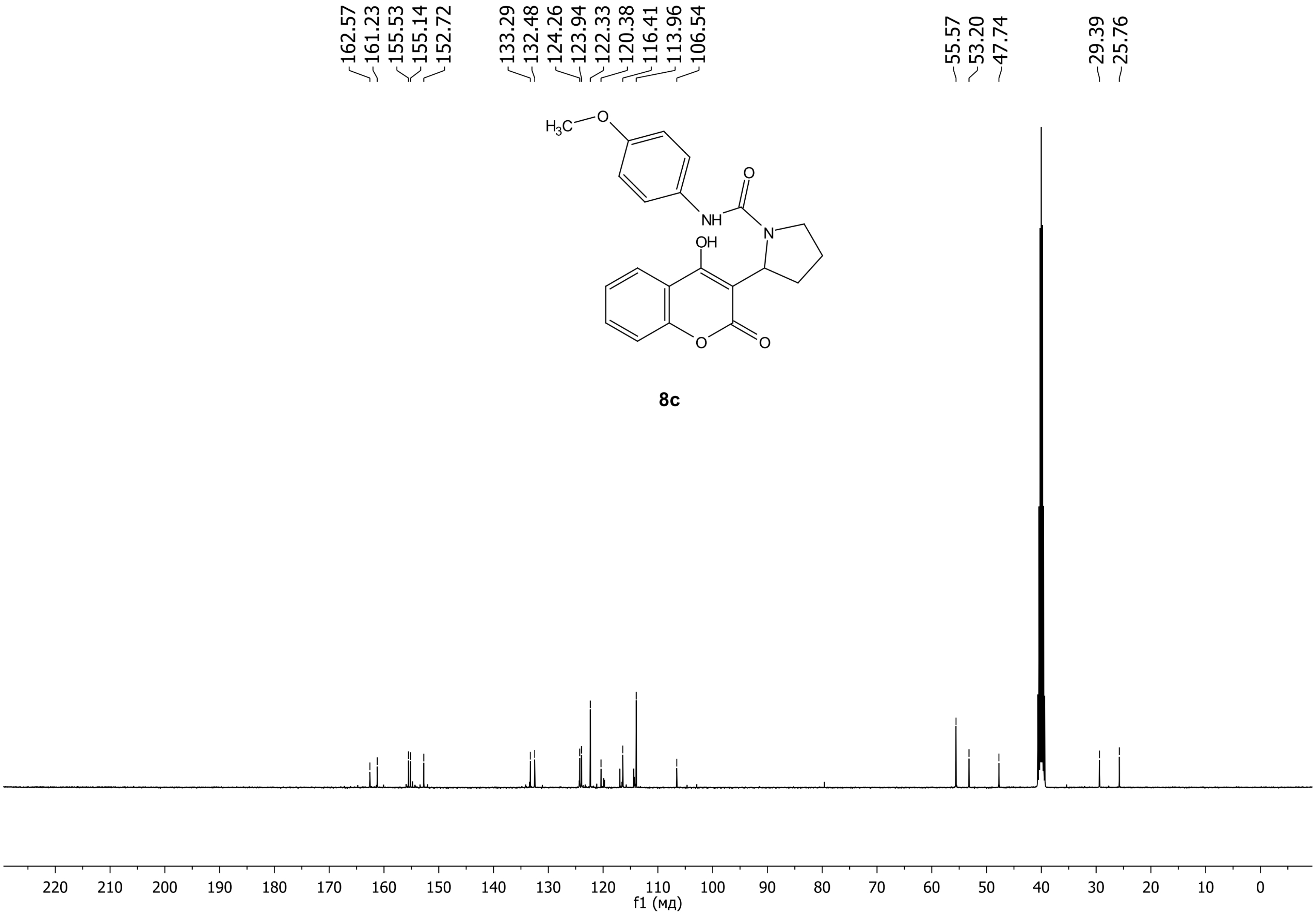
162.57
161.23
155.53
155.14
152.72
133.29
132.48
124.26
123.94
122.33
120.38
116.41
113.96
106.54
55.57
53.20
47.74
29.39
25.76
H3C
O
O
NH
N
OH
O
O
8c
220 210 200 190 180 170 160 150 140 130 120 110 100 90 80 70 60 50 40 30 20 10 0
f1 (мд)

Figure S 71.

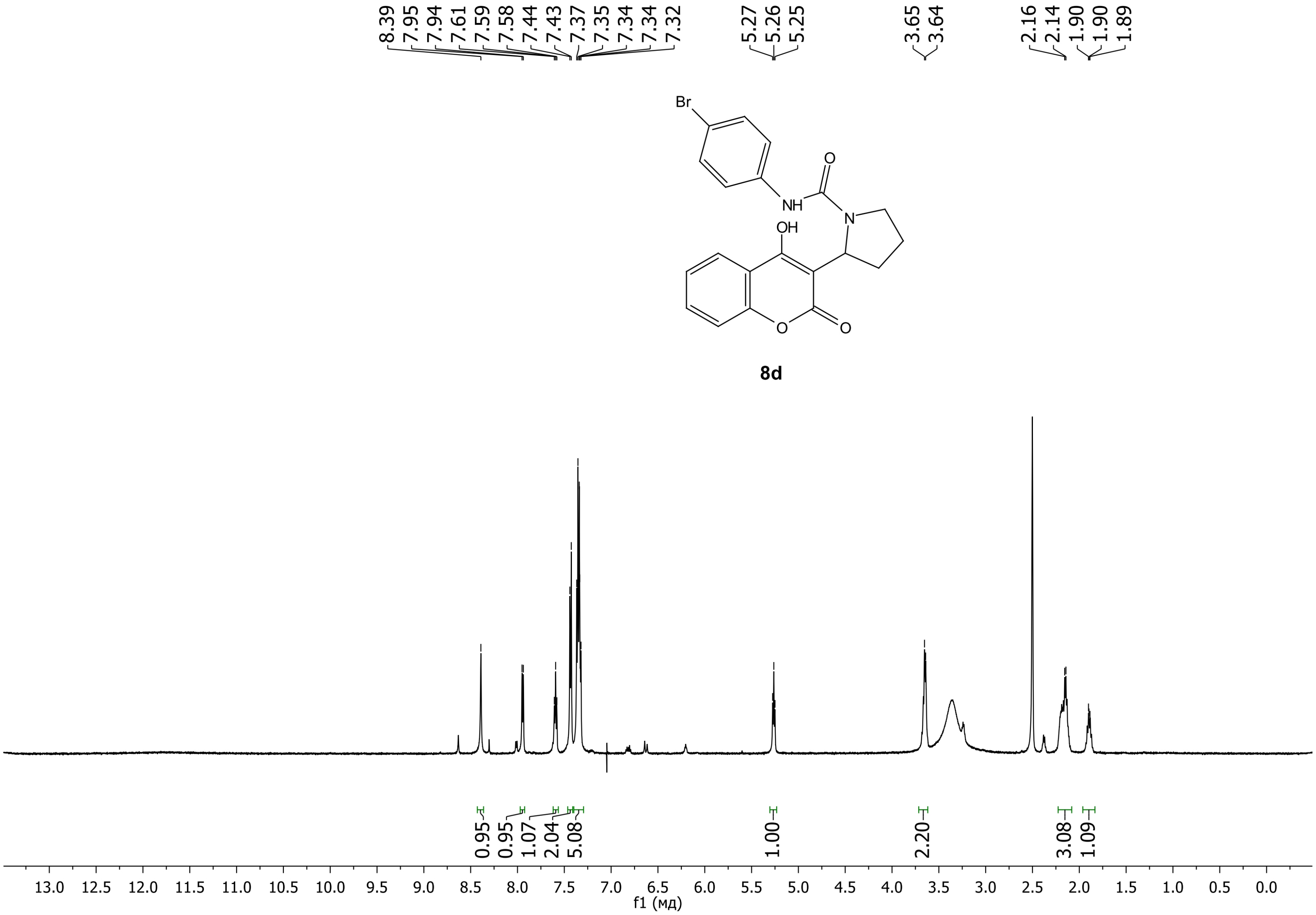
8.39
7.95
7.94
7.61
7.59
7.58
7.44
7.43
7.37
7.35
7.34
7.34
7.32
5.27
5.26
5.25
3.65
3.64
2.16
2.14
1.90
1.90
1.89
Br
O
NH
N
OH
O
O
8d
0.95
0.95
1.07
2.04
5.08
1.00
2.20
3.08
1.09
13.0 12.5 12.0 11.5 11.0 10.5 10.0 9.5 9.0 8.5 8.0 7.5 7.0 6.5 6.0 5.5 5.0 4.5 4.0 3.5 3.0 2.5 2.0 1.5 1.0 0.5 0.0
f1 (мд)

Figure S 72.

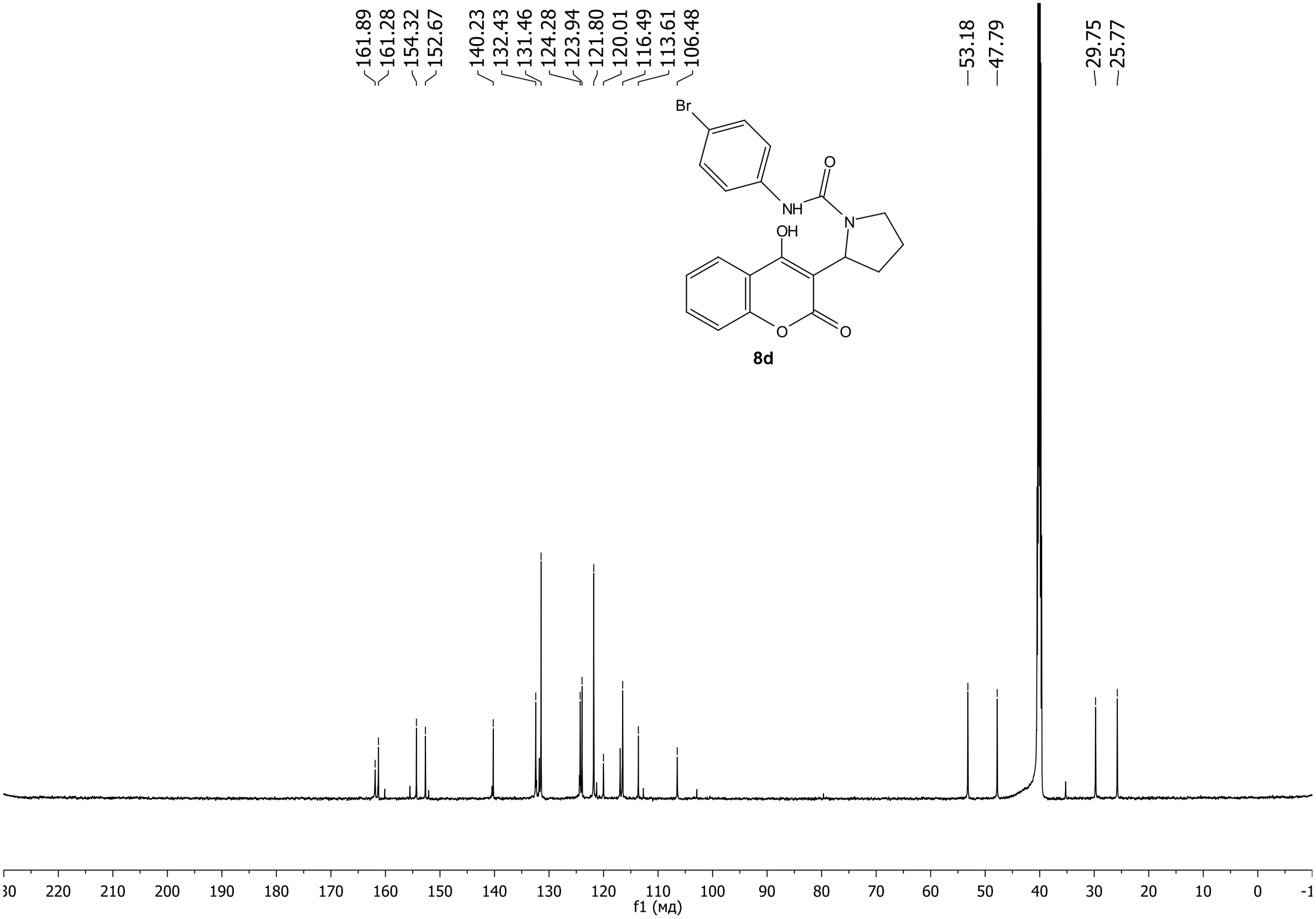
161.89
161.28
154.32
152.67
140.23
132.43
131.46
124.28
123.94
121.80
120.01
116.49
113.61
106.48
53.18
47.79
29.75
25.77
Br
O
NH
N
OH
O
O
8d
f1 (мд)

Figure S 73.

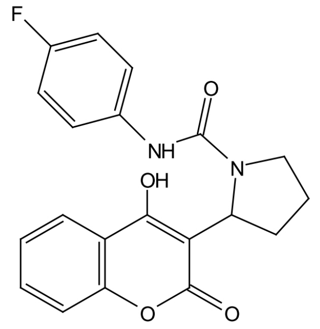

8e

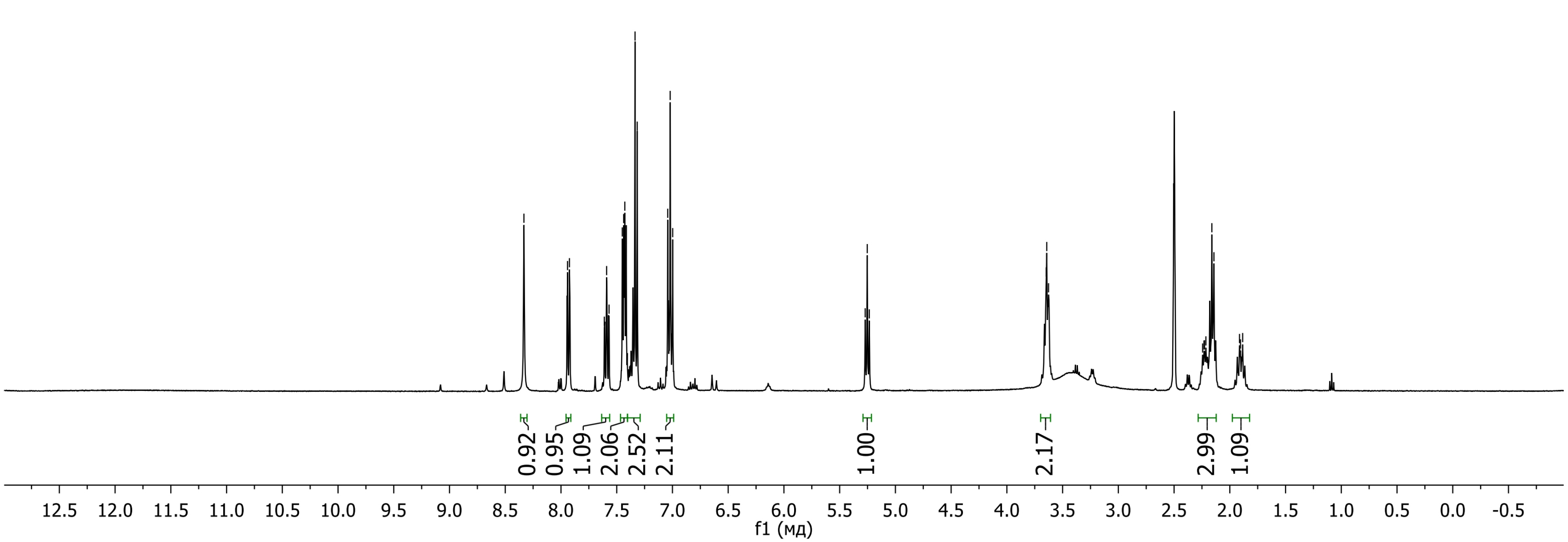

Figure S 74.

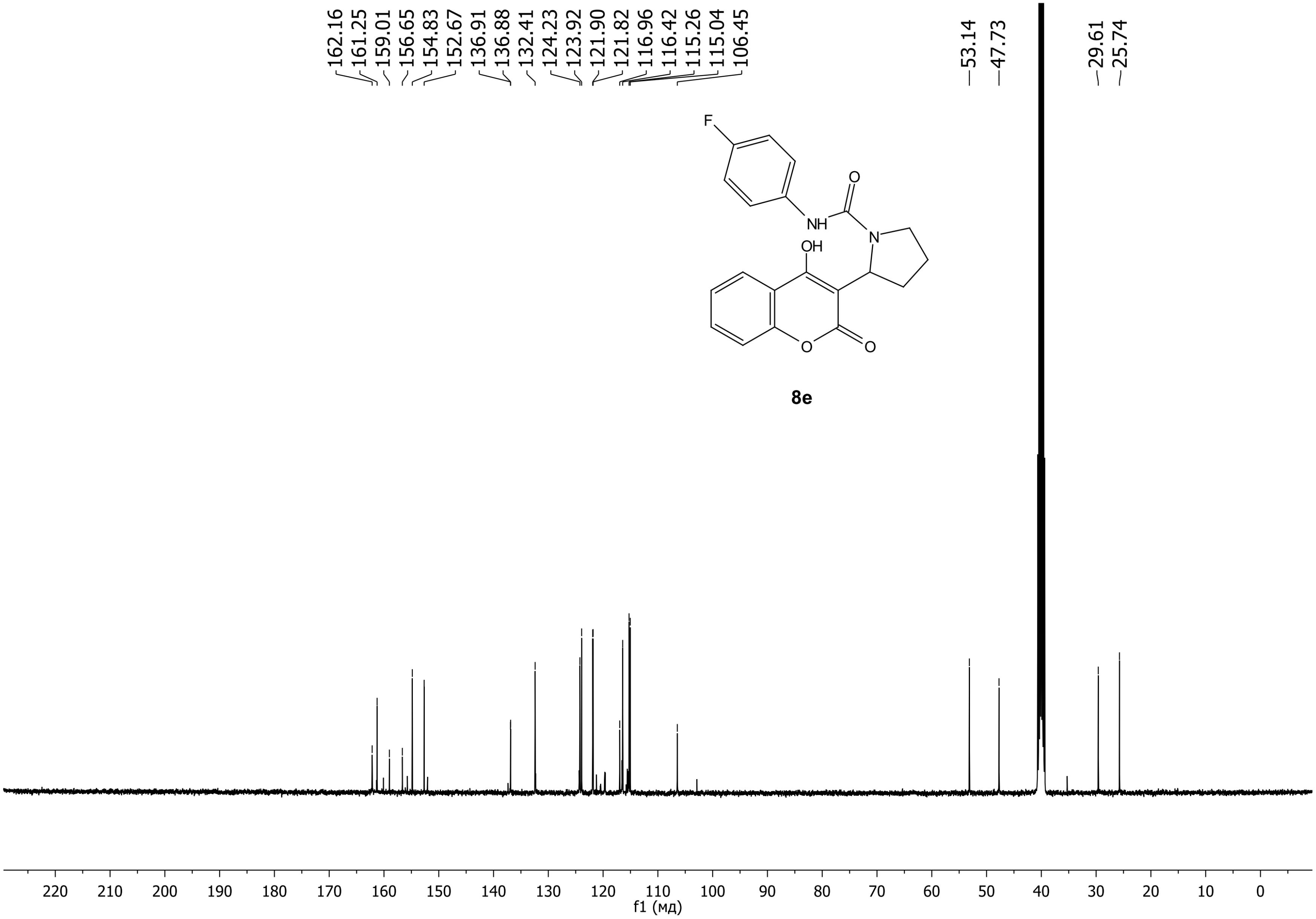
162.16
161.25
159.01
156.65
154.83
152.67
136.91
136.88
132.41
124.23
123.92
121.90
121.82
116.96
116.42
115.26
115.04
106.45
53.14
47.73
29.61
25.74
F
O
NH
N
OH
O
O
8e
220 210 200 190 180 170 160 150 140 130 120 110 100 90 80 70 60 50 40 30 20 10 0
f1 (мд)

Figure S 75.

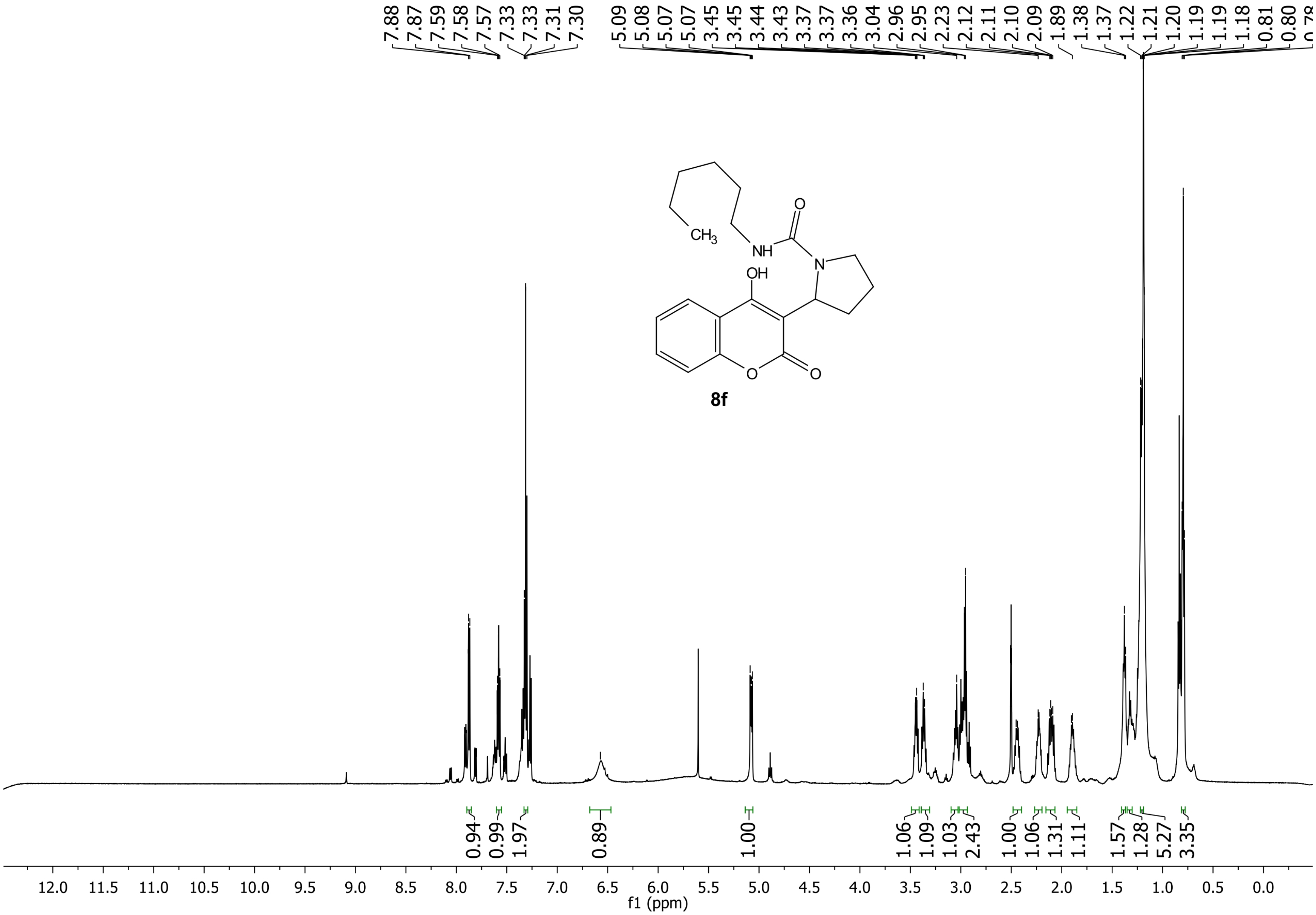

Figure S 76.

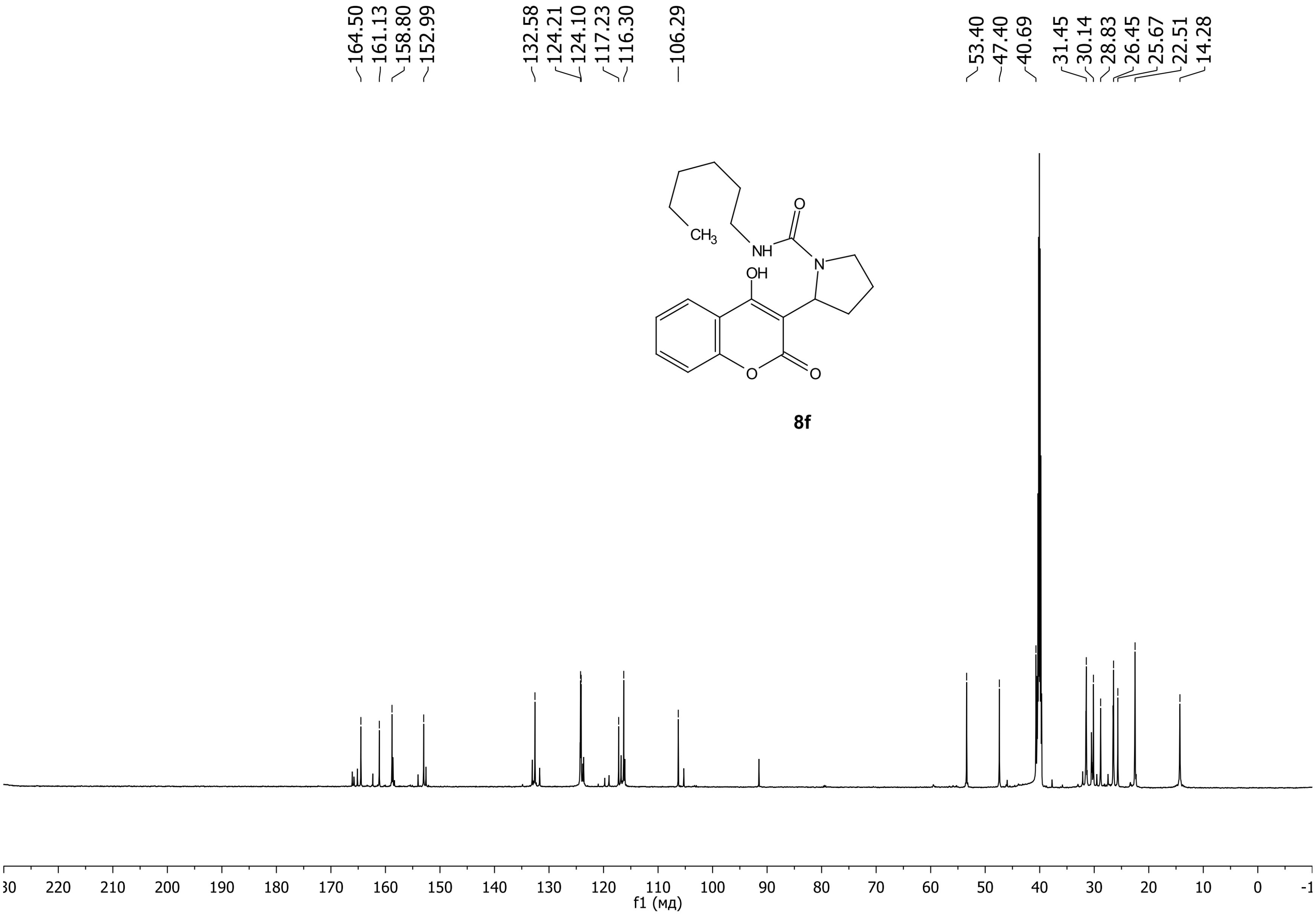
164.50
161.13
158.80
152.99
132.58
124.21
124.10
117.23
116.30
106.29
53.40
47.40
40.69
31.45
30.14
28.83
26.45
25.67
22.51
14.28
OH
O
NH
N
CH3
8f
f1 (мд)

Figure S 77.

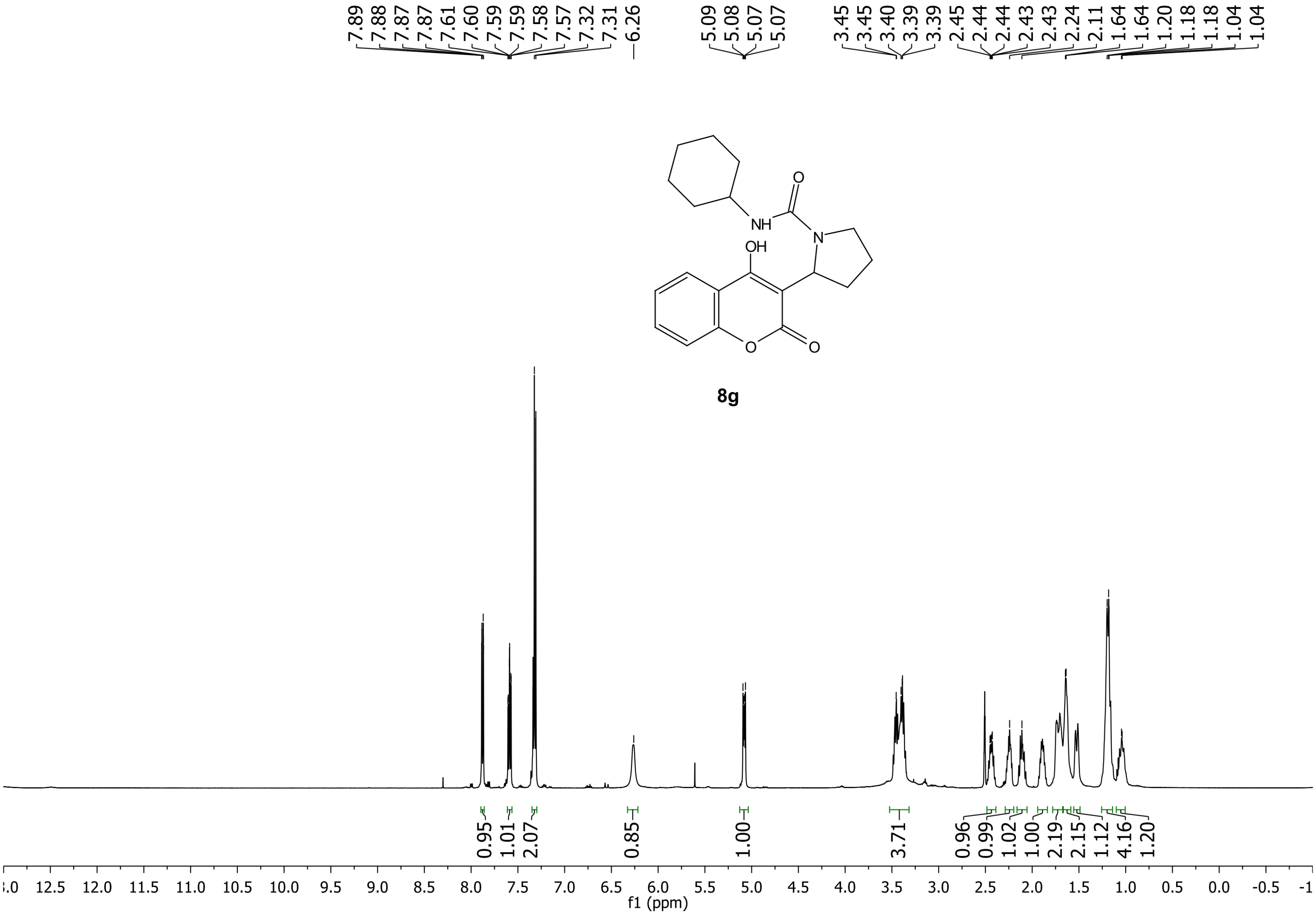
7.89 7.88 7.87 7.87 7.61 7.60 7.59 7.59 7.58 7.57 7.32 7.31 6.26
5.09 5.08 5.07 5.07
3.45 3.45 3.40 3.39 3.39 2.45 2.44 2.44 2.43 2.43 2.24 2.11 1.64 1.64 1.20 1.18 1.18 1.04 1.04
O
NH
N
OH
O
O
8g
0.95 1.01 2.07 0.85 1.00 3.71 0.96 0.99 1.02 1.00 2.19 2.15 1.12 4.16 1.20
12.5 12.0 11.5 11.0 10.5 10.0 9.5 9.0 8.5 8.0 7.5 7.0 6.5 6.0 5.5 5.0 4.5 4.0 3.5 3.0 2.5 2.0 1.5 1.0 0.5 0.0 -0.5
f1 (ppm)

Figure S 78.

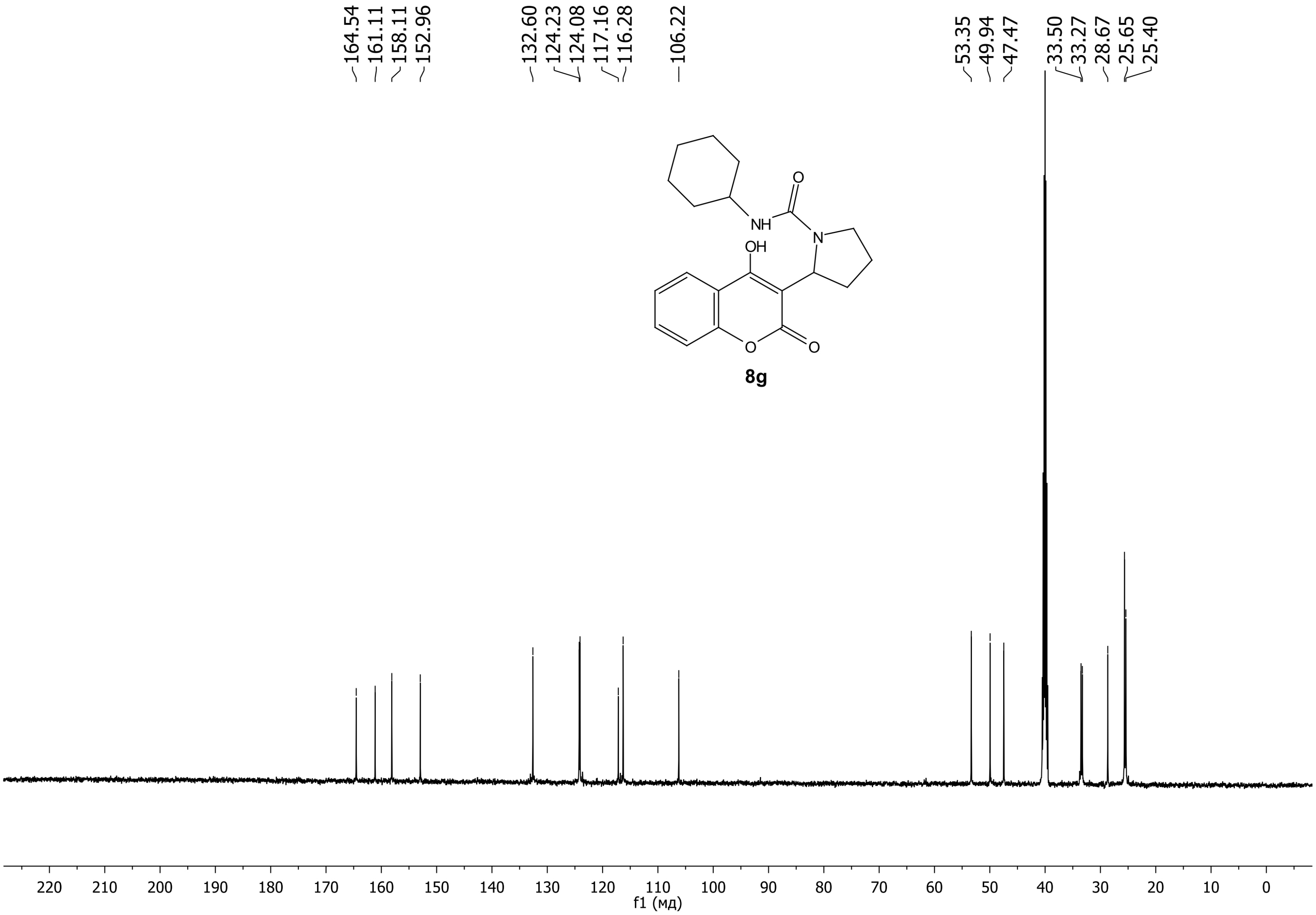
164.54
161.11
158.11
152.96
132.60
124.23
124.08
117.16
116.28
106.22
53.35
49.94
47.47
33.50
33.27
28.67
25.65
25.40
O
NH
N
OH
O
O
8g
220 210 200 190 180 170 160 150 140 130 120 110 100 90 80 70 60 50 40 30 20 10 0
f1 (мд)

Figure S 79.

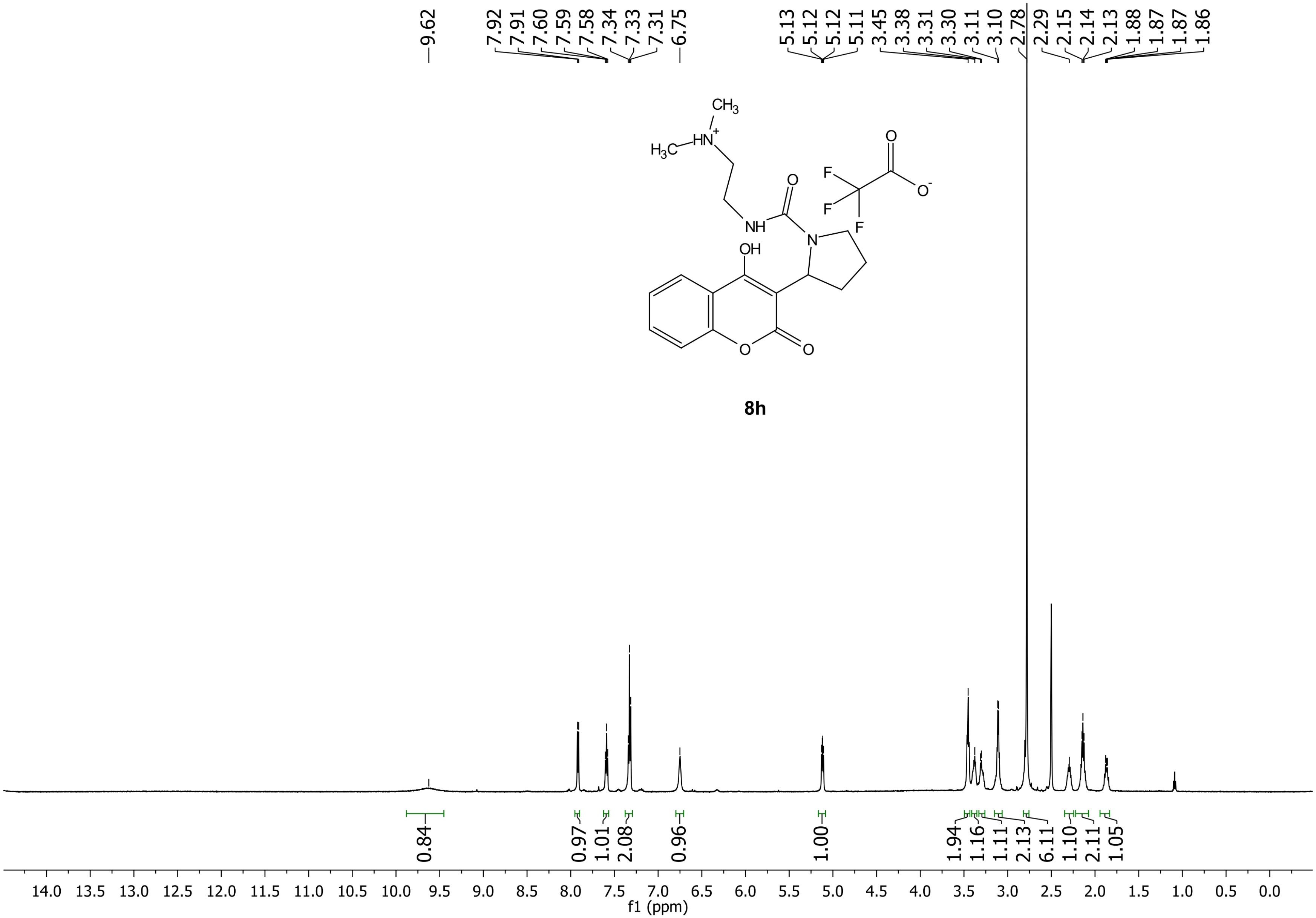
9.62
7.92 7.91 7.60 7.59 7.58 7.34 7.33 7.31 6.75
5.13 5.12 5.12 5.11 3.45 3.38 3.31 3.30 3.11 3.10 2.78 2.29 2.15 2.14 2.13 1.88 1.87 1.87 1.86
8h
0.84 0.97 1.01 2.08 0.96 1.00 1.94 1.16 1.11 2.13 6.11 1.10 2.11 1.05
14.0 13.5 13.0 12.5 12.0 11.5 11.0 10.5 10.0 9.5 9.0 8.5 8.0 7.5 7.0 6.5 6.0 5.5 5.0 4.5 4.0 3.5 3.0 2.5 2.0 1.5 1.0 0.5 0.0
f1 (ppm)

Figure S 80.

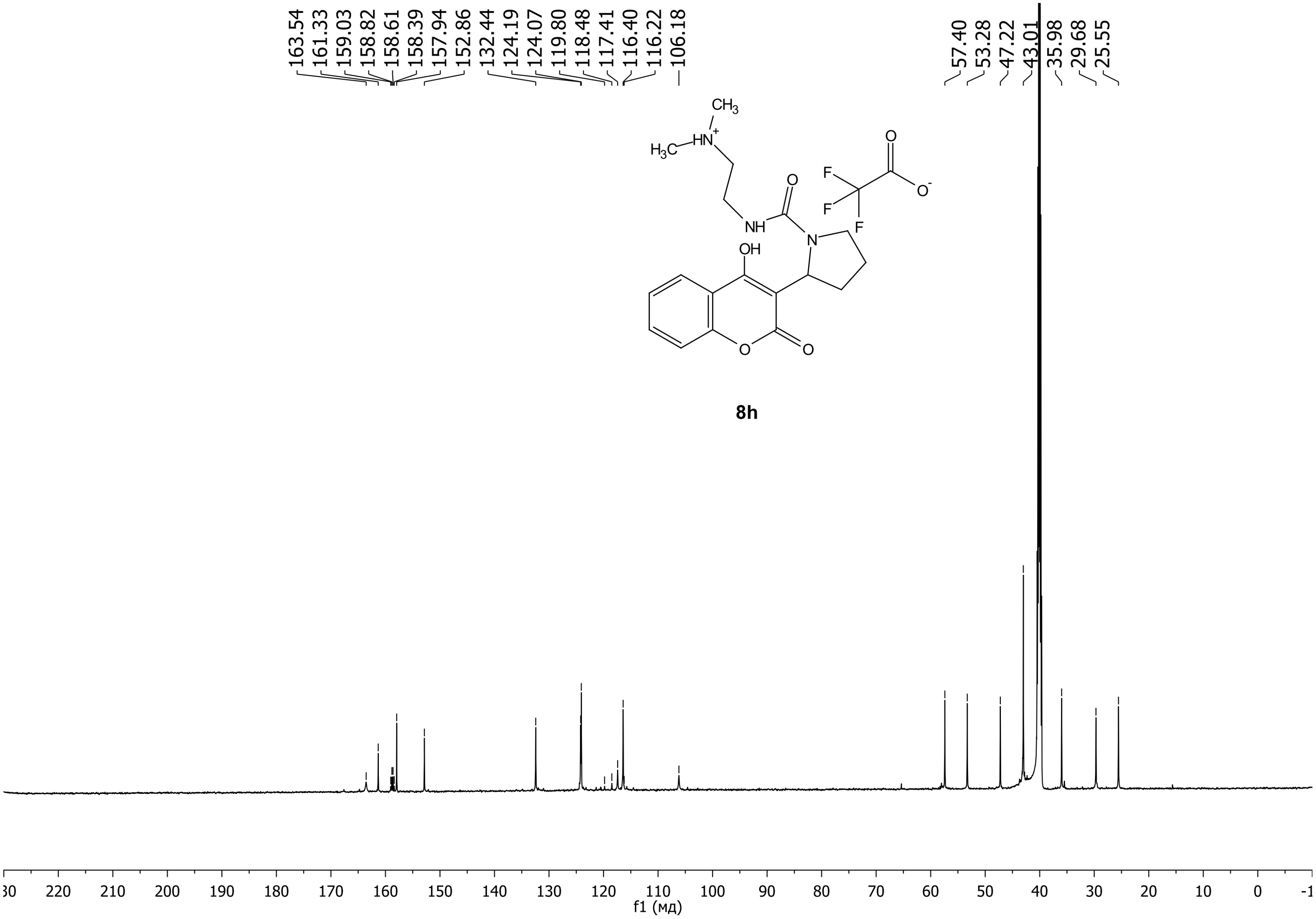
163.54
161.33
159.03
158.82
158.61
158.39
157.94
152.86
132.44
124.19
124.07
119.80
118.48
117.41
116.40
116.22
106.18
57.40
53.28
47.22
43.01
35.98
29.68
25.55
8h
f1 (мд)

Figure S 81.
